# Endosymbiosis in trypanosomatids: the genomic cooperation between bacterium and host in the synthesis of essential amino acids is heavily influenced by multiple horizontal gene transfers

**DOI:** 10.1186/1471-2148-13-190

**Published:** 2013-09-09

**Authors:** João MP Alves, Cecilia C Klein, Flávia Maia da Silva, André G Costa-Martins, Myrna G Serrano, Gregory A Buck, Ana Tereza R Vasconcelos, Marie-France Sagot, Marta MG Teixeira, Maria Cristina M Motta, Erney P Camargo

**Affiliations:** 1 Virginia Commonwealth University, Richmond, VA, USA; 2 BAMBOO Team, INRIA Grenoble-Rhône-Alpes, Villeurbanne, France; 3 Laboratoire Biométrie et Biologie Evolutive, Université de Lyon, Université Lyon 1, CNRS, UMR5558, Villeurbanne, France; 4 Laboratório Nacional de Computação Científica, Petrópolis, Rio de Janeiro, Brazil; 5 Department of Parasitology, Institute of Biomedical Sciences, University of São Paulo, São Paulo, Brazil; 6 Laboratório de Ultraestrutura Celular Hertha Meyer. Instituto de Biofísica Carlos Chagas Filho, Universidade Federal do Rio de Janeiro, Rio de Janeiro, Brazil

**Keywords:** Endosymbiosis, Trypanosomatids, Amino acid biosynthesis, Phylogeny, Genomic analyses, Metabolic pathway evolution, Proteobacteria

## Abstract

**Background:**

Trypanosomatids of the genera *Angomonas* and *Strigomonas* live in a mutualistic association characterized by extensive metabolic cooperation with obligate endosymbiotic Betaproteobacteria. However, the role played by the symbiont has been more guessed by indirect means than evidenced. Symbiont-harboring trypanosomatids, in contrast to their counterparts lacking symbionts, exhibit lower nutritional requirements and are autotrophic for essential amino acids. To evidence the symbiont’s contributions to this autotrophy, entire genomes of symbionts and trypanosomatids with and without symbionts were sequenced here.

**Results:**

Analyses of the essential amino acid pathways revealed that most biosynthetic routes are in the symbiont genome. By contrast, the host trypanosomatid genome contains fewer genes, about half of which originated from different bacterial groups, perhaps only one of which (ornithine cyclodeaminase, EC:4.3.1.12) derived from the symbiont. Nutritional, enzymatic, and genomic data were jointly analyzed to construct an integrated view of essential amino acid metabolism in symbiont-harboring trypanosomatids. This comprehensive analysis showed perfect concordance among all these data, and revealed that the symbiont contains genes for enzymes that complete essential biosynthetic routes for the host amino acid production, thus explaining the low requirement for these elements in symbiont-harboring trypanosomatids. Phylogenetic analyses show that the cooperation between symbionts and their hosts is complemented by multiple horizontal gene transfers, from bacterial lineages to trypanosomatids, that occurred several times in the course of their evolution. Transfers occur preferentially in parts of the pathways that are missing from other eukaryotes.

**Conclusion:**

We have herein uncovered the genetic and evolutionary bases of essential amino acid biosynthesis in several trypanosomatids with and without endosymbionts, explaining and complementing decades of experimental results. We uncovered the remarkable plasticity in essential amino acid biosynthesis pathway evolution in these protozoans, demonstrating heavy influence of horizontal gene transfer events, from Bacteria to trypanosomatid nuclei, in the evolution of these pathways.

## Background

Many protozoan and metazoan cells harbor vertically inherited endosymbionts in their cytoplasm. Prominent among them are the associations between Alphaproteobacteria and leguminous root cells, as well as Gammaproteobacteria and cells lining the digestive tube of insects. Comprehensive reviews have covered most aspects of these ancient mutualistic relationships, including metabolism, genetics, and evolutionary history of the endosymbiont/host cell associations [1-7]. Much less is known about the relationship between protists and their bacterial endosymbionts [8-10], including the symbiosis between trypanosomatids and Betaproteobacteria, herein examined [11-14].

The Trypanosomatidae (Euglenozoa, Kinetoplastea) are well studied mainly because species of the genera *Trypanosoma* and *Leishmania* are pathogenic in humans and domestic animals [15]. However, despite their importance, these pathogens are a minority within the family, and most species are non-pathogenic commensals in the digestive tube of insects [16-18]. Usually, trypanosomatids are nutritionally fastidious and require very rich and complex culture media, however a very small group of these protozoa can be cultivated in very simple and defined media [19-23]. This reduced group of insect trypanosomatids carries cytoplasmic endosymbionts and is known as symbiont-harboring trypanosomatids, to distinguish them from regular insect trypanosomatids naturally lacking symbionts. Symbiont-harboring trypanosomatids belong to the genera *Strigomonas* and *Angomonas*[24], and their lesser nutritional requirements indicate that they have enhanced biosynthetic capabilities. In a few cases, it has been shown that the symbiotic bacterium contains enzymes involved in host biosynthetic pathways, but in most cases the metabolic contribution of the endosymbiont has been inferred from nutritional data rather than genetically demonstrated [12,14].

Each symbiont-harboring trypanosomatid carries just one symbiont in its cytoplasm, which divides synchronously with other host cell structures and is vertically transmitted [14]. The endosymbionts’ original association with an ancestral trypanosomatid is thought to have occurred sometime in the Cretaceous [13]. This long partnership has led to considerable changes on the endosymbiont genomes including gene loss, with clear preferential retention of genes involved in metabolic collaboration with the host, and consequent genomic size reduction [25,26], as seen in other obligatory symbiotic associations [1,2,7,27-29].

Extensive comparative studies between symbiont-harboring trypanosomatids (wild and cured strains, obtained after antibiotic treatment) and regular trypanosomatids have permitted inferences about the symbiont dependence and contribution in the overall metabolism, in particular phospholipid [30-32] and amino acid [33-41] production of the host cell. Previous comparative studies on these organisms, often involving trace experiments using radioactive compounds, reported the requirement, substitution, and sparing of amino acids in culture media [22,33,42-55]. Nutritional data revealed that, as for most animals, including humans, the amino acids lysine, histidine, threonine, isoleucine, leucine, methionine, cysteine, tryptophan, valine, phenylalanine, tyrosine, and arginine/citrulline are essential for regular trypanosomatids. However, similar analyses showed that symbiont-harboring trypanosomatids require only methionine or tyrosine in culture media, suggesting that they possess the necessary enzymatic equipment to synthesize most amino acids [20,22,23,34]. Unfortunately, besides the symbiont-harboring trypanosomatids, most of these studies were performed only on *Crithidia fasciculata*, largely ignoring other trypanosomatids. Of the hundreds of enzymes known to be involved in the synthesis of essential amino acids in other organisms, only a few, i.e., diaminopimelic decarboxylase, threonine deaminase, ornithine carbamoyl transferase, argininosuccinate lyase, citrulline hydrolase, ornithine acetyl transferase, acetyl ornithinase, and arginase have been identified and characterized in trypanosomatids [21,33,35-41,46,56]. Thus, in contrast to the advanced state of knowledge of genes involved in amino acid biosynthesis in many microorganisms [57], the potential for amino acid synthesis in trypanosomatids remains largely unknown. In symbiont-harboring trypanosomatids, nutritional inferences provided little information about the effective participation of the symbiotic bacterium in the various metabolic pathways of the host protozoan. This contrasts with the advancement of knowledge about the presence/absence of genes for complete pathways for amino acid synthesis in many microorganisms.

Herein, we have identified the genes involved in the biosynthetic pathways of the essential amino acids in the genomes of symbiont-harboring and regular trypanosomatids of different genera (see Methods), through the characterization of each gene by similarity searches and protein domain analyses. We apply extensive phylogenetic inferences to determine the most likely origins of these genes, as it has been previously shown that other important metabolic enzymes in trypanosomatids have been transferred from bacteria, other than the present symbiont [58]. Although detection of a gene with a presumed function does not definitely prove its activity, the association of its presence with complementary nutritional and biochemical data supports the conclusion that it functions as predicted. In the present work, we establish the clear and defined contribution of endosymbionts to the amino acid metabolism of their trypanosomatid hosts, which is related to high amounts of lateral transfer of genes from diverse bacterial lineages to trypanosomatid genomes.

## Results and discussion

In this work, the presence or absence of a given gene for a particular enzyme was verified in the genomes of endosymbionts, symbiont-harboring and regular trypanosomatids and then compared with the available nutritional and enzymatic data on essential amino acid biosynthesis in insect trypanosomatids. Extensive phylogenetic analyses were also performed on most of the identified trypanosomatid genes, in addition to some symbiont genes of interest. Data are mostly limited to the regular and symbiont-harboring trypanosomatid and endosymbiont genomes that have been sequenced here. Although the genomes of all available symbiont-harboring trypanosomatids and endosymbionts have been examined, only a very limited sample of regular trypanosomatid genomes (*H. muscarum* and *C. acanthocephali*) was included in these analyses, precluding generalizations about trypanosomatids as a whole. Data on the genomes of leishmaniae and trypanosomes available in KEGG were also used for comparison, but a wider sampling of genomes from more diverse groups of Trypanosomatidae and other, more distant Kinetoplastida will be necessary to enable more generalizing conclusions on the evolution of essential amino acid synthesis pathways in these organisms.

Given the incomplete nature of the trypanosomatid genomes sequenced here and the possibility of contaminant sequences, we have taken extensive precautions before including each gene in our analyses (see Methods). Our genomic context analyses of the genes identified as horizontally transferred show (Additional file 1) that genes used in this work occurred, with one exception, in long contigs presenting the typical trypanosomatid architecture of long stretches of genes in the same orientation. Moreover, all these genes overwhelmingly matched those from previously sequenced trypanosomatids. The one exception is a gene (2.7.1.100, see below) that occurs only in the two regular trypanosomatids sequenced here, and whose sequences are isolated in short contigs. As described below, they form a monophyletic group in the phylogeny. GC percent (Additional file 1) and sequencing coverage (Additional file 2) analyses also show that all genes identified in this work present statistics typical of other genes from these organisms. In short, these data show that the trypanosomatid genes employed here are highly unlikely to be contaminants.

### Pathways of amino acid synthesis

#### Lysine

Lysine, as well as methionine and threonine, are essential amino acids generated from aspartate, a non-essential amino acid, which is synthesized from oxaloacetate that is produced in the Krebs cycle. There are two main routes for the biosynthesis of lysine: the diaminopimelate (DAP) and the aminoadipate (AA) pathways. The former is largely confined to bacteria, algae, some fungi, and plants, whereas the latter is described in fungi and euglenids [59-63].

**Figure 1:**
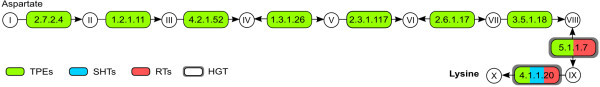
**DAP pathway for lysine biosynthesis.** Enzymes surrounded by a thick gray box were shown to be horizontally transferred from Bacteria (see main text). Metabolites – **I**: L-aspartate; **II**: 4-aspartyl-phosphate; **III**: aspartate 4-semialdehyde; **IV**: 2,3-dihydrodipicolinate; **V**: 2,3,4,5-tetrahydrodipicolinate; **VI**: N-succinyl-L-2-amino-6-oxopimelate; **VII**: N-succinyl-LL-2,6-diaminopimelate; **VIII**: LL-2,6-diaminopimelate; **IX**: meso-2,6-diaminopimelate; **X**: lysine. Enzymes – 2.7.2.4: aspartate kinase; 1.2.1.11: aspartate-semialdehyde dehydrogenase; 4.2.1.52: dihydrodipicolinate synthase; 1.3.1.26: dihydrodipicolinate reductase; 2.3.1.117: tetrahydrodipicolinate succinyltransferase; 2.6.1.17: succinyldiaminopimelate transaminase; 3.5.1.18: succinyldiaminopimelate desuccinylase; 5.1.1.7: diaminopimelate epimerase; 4.1.1.20: diaminopimelate decarboxylase. SHT: symbiont-harboring trypanosomatid; RT: regular trypanosomatid; TPE: trypanosomatid proteobacterial endosymbiont.

Early nutritional studies [46] showed that lysine is essential for the growth of regular trypanosomatids, but could be efficiently replaced by DAP. In accordance, radioactive tracer and enzymatic experiments revealed that DAP is readily incorporated as lysine into proteins. Moreover, DAP-decarboxylase (EC:4.1.1.20), the enzyme that converts DAP into lysine, was detected in cell homogenates of *C. fasciculata*[46]. Nevertheless, either lysine or DAP were always necessary for growth of these flagellates in defined medium, indicating that the lysine pathway was somehow incomplete. In contrast, symbiont-harboring trypanosomatids required neither lysine nor DAP to grow in defined media [19-23]. Interestingly, the genes encoding the nine enzymes of the bacterial-type DAP pathway, leading from aspartate to lysine, were identified in the genomes of all endosymbionts (Figure 1). In contrast, only the final gene of the DAP pathway was found in the genomes of the symbiont-harboring trypanosomatids, and the final two found in one regular trypanosomatid examined (*H. muscarum*), which explains why DAP could substitute for lysine in growth media of some regular trypanosomatids. There are no genes for lysine biosynthesis annotated in the leishmaniae and trypanosomes present in KEGG. It is worth mentioning that, with respect to the alternative AA pathway, we were unable to find any genes for the synthesis of lysine in any of the endosymbiont, symbiont-harboring or regular trypanosomatid genomes analyzed.

In summary, our findings using comparative genomics are in concordance with the data from previous nutritional and enzymatic studies, showing that only symbiont-harboring trypanosomatids, and not regular ones, are autotrophic for lysine and that this autonomy is provided by the DAP pathway present in their symbionts. The presence of DAP-decarboxylase in symbiont-harboring trypanosomatids may suggest that although the symbiont contains the great majority of genes for the lysine production, the host protozoan somehow controls the production of this essential amino acid.

### Methionine and cysteine

Methionine is included in all defined media designed for the growth of trypanosomatids with or without symbionts [20,22,43], suggesting that these protozoans are incapable of methionine synthesis. However, experimental evidence has shown that homocysteine and/or cystathionine could substitute for methionine in culture media for trypanosomatids [43,45,64].

**Figure 2:**
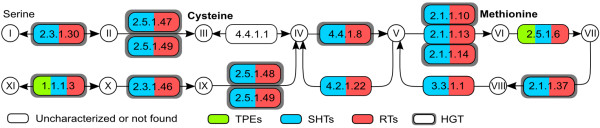
**Cysteine and methionine synthesis and interconversion pathway.** Enzymes surrounded by a thick gray box were shown to be horizontally transferred from Bacteria (see main text). Metabolites – **I**: L-serine; **II**: O-acetyl-serine; **III**: cysteine; **IV**: cystathionine; **V**: homocysteine; **VI**: methionine; **VII**: S-adenosyl-methionine; **VIII**: S-adenosyl-homocysteine; **IX**: succinyl-homoserine; **X**: homoserine; **XI**: aspartate 4-semialdehyde. Enzymes – 2.3.1.30: serine O-acetyltransferase; 2.5.1.47: cysteine synthase; 4.4.1.1: cystathionine gamma-lyase; 4.4.1.8: cystathionine beta-lyase; 2.1.1.x: 2.1.1.10, homocysteine S-methyltransferase, 2.1.1.13, 5-methyltetrahydrofolate–homocysteine methyltransferase, 2.1.1.14, 5-methyltetrahydropteroyltriglutamate–homocysteine methyltransferase; 2.5.1.6: S-adenosyl-methionine synthetase; 2.1.1.37: DNA (cytosine-5-)-methyltransferase; 3.3.1.1: adenosylhomocysteinase; 4.2.1.22: cystathionine beta-synthase; 2.5.1.48: cystathionine gamma-synthase; 2.3.1.46: homoserine O-succinyltransferase; 1.1.1.3: homoserine dehydrogenase. Abbreviations as for Figure 1.

Our analyses suggest that regular trypanosomatids and symbiont-harboring trypanosomatids have the necessary genes to produce cystathionine, homocysteine, and methionine from homoserine (Figure 2), whereas the endosymbiont genomes have no gene for the enzymes involved in the synthesis of methionine from homoserine. However, homoserine is produced from aspartate semialdehyde through the mediation of homocysteine methyltransferase (EC:1.1.1.3), which is universally present in the genomes of all the endosymbionts, symbiont-harboring and regular trypanosomatids examined.

With respect to cysteine synthesis, it has been shown that the incubation of cell homogenates of *C. fasciculata* with ^35^S-methionine produced radioactive adenosyl-methionine (SAM), adenosyl-homocysteine (SAH), homocysteine, cystathionine, and cysteine [45]. Thus, this trypanosomatid is fully equipped to methylate methionine to produce homocysteine and, thereon, to convert homocysteine into cysteine through the trans-sulfuration pathway. However, with respect to the cystathionine/cysteine interconversion, there is some ambiguity concerning the presence or absence of cystathionine gamma-lyase (EC:4.4.1.1) in regular trypanosomatids. Many sulfhydrolases have a domain composition very similar to that of EC:4.4.1.1, which makes a definitive *in silico* function assignment to any of them difficult. Specifically, the enzymes cystathionine gamma-synthase (EC:2.5.1.48) and O-acetylhomoserine aminocarboxypropyltransferase (EC:2.5.1.49), and the two versions of cystathionine beta-lyase (EC:4.4.1.8) are possible candidates to mediate the trans-sulfuration step attributed to EC:4.4.1.1, but further research is required to establish which of these enzymes, if any, performs that reaction. We also found that, in addition to the standard pathway for methionine/cysteine synthesis (Figure 2, compounds III-X), all symbiont-harboring and regular trypanosomatids examined had the genes to produce cysteine from serine in a simple two-step reaction, with acetylserine as an intermediate (Figure 2, I-III).

In summary, if regular and symbiont-harboring trypanosomatids are capable of interconverting methionine and cysteine, as shown for *C. fasciculata*[43], none of these two amino acids can be considered essential for trypanosomatids as the presence of one renders the other unnecessary. In that case, both can be synthesized by trypanosomatids, without any participation of their symbionts, except in the optional production of aspartate semialdehyde and homoserine. However, the expression of these genes remains to be confirmed.

### Threonine

In trypanosomatids, initial investigations about the nutritional requirements for threonine were controversial. Most results suggested that this amino acid is essential [43,45,48,64-66], but other studies considered the addition of threonine to the growth media of regular trypanosomatids unnecessary [33]. Our genomic analysis favors the latter observations.

**Figure 3:**
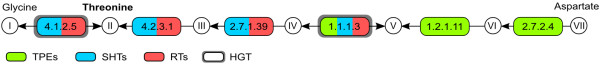
**Threonine synthesis pathway.** Enzymes surrounded by a thick gray box were shown to be horizontally transferred from Bacteria (see main text). Metabolites – **I**: glycine; **II**: threonine; **III**: phosphohomoserine; **IV**: homoserine; **V**: aspartate 4-semialdehyde; **VI**: 4-aspartyl-phosphate; **VII**: L-aspartate. Enzymes – 4.1.2.5: threonine aldolase; 2.7.2.4: aspartate kinase; 1.2.1.11: aspartate-semialdehyde dehydrogenase; 1.1.1.3: homoserine dehydrogenase; 2.7.1.39: homoserine kinase; 4.2.3.1: threonine synthase. Abbreviations as for Figure 1.

Threonine, one of the precursors of isoleucine, can be produced by different biosynthetic pathways. We have examined two of these possible routes, one starting from glycine and the other from aspartate, as presented in Figure 3. The conversion of glycine plus acetoaldehyde into threonine is mediated by threonine aldolase (EC:4.1.2.5). The gene for this enzyme is absent from endosymbionts but present in the genomes of symbiont-harboring trypanosomatids and *C. acanthocephali*, but not *Herpetomonas*. It is also absent from the genomes of trypanosomes but present in the genome of *Leishmania major* (KEGG data).

The pathway from aspartate utilizes the first two enzymes (EC:2.7.2.4 and EC:1.2.1.11) of the DAP pathway from lysine synthesis for the production of aspartate semialdehyde. These genes are present exclusively in the symbiont genomes. Aspartate semialdehyde is then sequentially converted into homoserine, phosphohomoserine, and threonine. The gene encoding homoserine dehydrogenase (EC:1.1.1.3) is universally present in the genomes of the endosymbionts, symbiont-harboring and regular trypanosomatids. It is also present in the genomes of *T. cruzi* and *Leishmania* spp. In contrast, the genes for the enzymes leading from homoserine to threonine via phosphohomoserine (EC:2.7.1.39 and EC:4.2.3.1) are present in the genomes of all insect trypanosomatids (including symbiont-harboring ones), of *Trypanosoma* spp., and *Leishmania* spp., but totally absent from the endosymbiont genomes.

Thus, the genetic constitution of regular trypanosomatids is consistent with earlier nutritional data showing the insect trypanosomatids, with or without symbionts, to be autotrophic for threonine. This observation suggests that endosymbionts are able to enhance the host cell threonine synthesis by producing the metabolic precursor aspartate semialdehyde that is also involved in other metabolic pathways.

The overall genomic and enzymatic picture is in apparent contradiction with early nutritional findings showing that threonine promoted the growth of trypanosomatids in culture [67]. This contradiction might find its basis in the fact that endogenously produced threonine is required by many metabolic processes, such that supplementation of the culture media could enhance the growth of the trypanosomatids.

### Isoleucine, valine, and leucine

**Figure 4:**
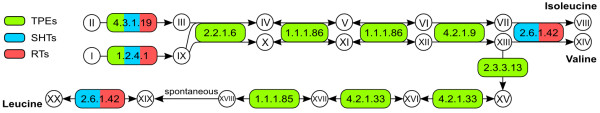
**Isoleucine, valine, and leucine synthesis pathway.** Metabolites – **I**: pyruvate; **II**: threonine; **III**: 2-oxobutanoate; **IV**: (S)-2-aceto-2-hydroxybutanoate; **V**: (R)-3-hydroxy-3-methyl-2-oxopentanoate; **VI**: (R)-2,3-dihydroxy-3-methylpentanoate; **VII**: (S)-3-methyl-2-oxopentanoate; **VIII**: isoleucine; **IX**: 2-(alpha-hydroxyethyl) thiamine diphosphate; **X**: (S)-2-acetolactate; **XI**: 3-hydroxy-3-methyl-2-oxobutanoate; **XII**: (R)-2,3-dihydroxy-3-methylbutanoate; **XIII**: 2-oxoisovalerate; **XIV**: valine; **XV**: (2S)-2-isopropylmalate; **XVI**: 2-isopropylmaleate; **XVII**: (2R,3S)-3-isopropylmalate; **XVIII**: (2S)-2-isopropyl-3-oxosuccinate; **XIX**: 4-methyl-2-oxopentanoate; **XX**: leucine. Enzymes – 1.2.4.1: pyruvate dehydrogenase E1 component subunit alpha; 4.3.1.19: threonine ammonia-lyase; 2.2.1.6: acetolactate synthase small and large subunits; 1.1.1.86: ketol-acid reductoisomerase; 4.2.1.9: dihydroxy-acid dehydratase; 2.6.1.42: branched-chain amino acid transaminase; 2.3.3.13: 2-isopropylmalate synthase; 4.2.1.33: 3-isopropylmalate dehydratase small and large subunits; 1.1.1.85: 3-isopropylmalate dehydrogenase. Abbreviations as for Figure 1.

Isoleucine, valine, and leucine are considered essential nutrients for the growth of all trypanosomatids, except symbiont-harboring ones. The canonic pathway for the synthesis of isoleucine is depicted in Figure 4. Oxobutanoate (alpha-ketoglutaric acid) is the starting point of the pathway, and can be produced in two ways: from threonine (Figure 4, compounds II-III) or from pyruvate (Figure 4, compounds I, IX). The conversion of threonine into oxobutanoate is mediated by threonine deaminase (EC:4.3.1.19). The specific activity of this enzyme was higher in symbiont-enriched subcellular fractions of symbiont-harboring trypanosomatid homogenates than in any other cell fraction or in the cytosol, suggesting that this enzyme was located in the symbiont [33]. However, genes for EC:4.3.1.19 are present in the genomes of endosymbionts, as well as those of symbiont-harboring and regular trypanosomatids (except *Leishmania* and *Trypanosoma*), contrasting with enzymatic determinations showing the absence of enzyme activity in regular trypanosomatids [33]. Since the presence of the gene does not guarantee the functionality of the enzyme for that specific reaction, the issue remains to be experimentally verified. The next enzymatic step, the transference of the acetaldehyde from pyruvate to oxobutanoate, is mediated by the enzyme acetolactate synthase (EC:2.2.1.6), which is present exclusively in the genomes of endosymbionts. Also present only in symbionts are the genes for the next four enzymes of the pathway, which are common for valine and isoleucine synthesis. However, the gene for a branched-chain amino acid transaminase (EC:2.6.1.42), mediating the last step in the synthesis of isoleucine, valine, and leucine, is present in the genomes of symbiont-harboring and regular trypanosomatids, but not endosymbionts.

The first step of the valine pathway is the conversion of pyruvate into hydroxymethyl ThPP, mediated by an enzyme of the pyruvate dehydrogenase complex (EC:1.2.4.1) whose gene is present in the genomes of endosymbionts and symbiont-harboring and regular trypanosomatids. The next reaction, leading to acetolactate, is mediated by acetolactate synthase (EC:2.2.1.6), whose gene is present exclusively in the genomes of the endosymbionts. The reactions that follow from acetoacetate into valine involve the same endosymbiont genes from isoleucine synthesis.

Synthesis of leucine uses oxoisovalerate, an intermediate metabolite of the valine pathway that is converted into isopropylmalate by 2-isopropylmalate synthase (EC:2.3.3.13), encoded by a gene present only in the endosymbionts – as are the genes for the enzymes catalyzing the next three steps for leucine biosynthesis. The presence of the gene for this branched-chain amino acid transaminase (EC:2.6.1.42) in the genomes of regular trypanosomatids explains the earlier finding that oxopentanoate and oxoisovalerate, the immediate precursors of isoleucine, valine, and leucine could substitute for these amino acids when added to regular trypanosomatid synthetic culture media [43]. Interestingly, this gene is present in all symbiont-harboring and regular trypanosomatid genomes examined, but absent from endosymbiont genomes (Figure 4). It is also present in the genomes of *T. brucei* and the leishmaniae available from KEGG. In addition to isoleucine, valine, and leucine biosynthesis, this enzyme also participates in the degradation of these amino acids for their use in other metabolic processes in the cell, which might explain the presence of this enzyme as the only representative of the pathway in all regular trypanosomatids examined.

A coupled biosynthetic pathway of the branched-chain amino acids was also described for the symbiotic bacterium *Buchnera* and its aphid host, where the symbiont has the capability to synthesize the carbon skeleton of these amino acids but lacks the genes for the terminal transaminase reactions [68,69]. The aphid possesses genes hypothesized to accomplish these missing steps, even if orthologs of those are found in other insects and carry out different functions [70]. The branched-chain amino acid transaminase (EC:2.6.1.42) encoded by an aphid gene was shown to be up-regulated in the bacteriocytes, supporting the cooperation of *Buchnera* and its host in the synthesis of essential amino acids [71]. Since this transamination involves the incorporation of amino-N and the aphid diet is low in nitrogen, the host mediation of this step would be a way of maintaining a balanced profile of amino acids through transamination between those that are over abundant and those that are rare [71,72].

In summary, the presence in endosymbionts of most genes involved in isoleucine, valine and leucine synthesis explains why symbiont-harboring trypanosomatids, but not regular ones, are autotroph for these essential amino acids. However, it is worth noting that the presence of the branched-chain amino acid transaminase in trypanosomatids indicates that the host might control amino acid production according to their necessity and the nutrient availability in the medium.

### Phenylalanine, tyrosine, and tryptophan

**Figure 5:**
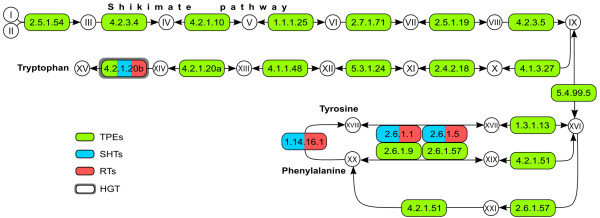
**Phenylalanine, tyrosine, and tryptophan synthesis pathway.** Enzymes surrounded by a thick gray box were shown to be horizontally transferred from Bacteria (see main text). Metabolites – **I**: D-erythrose 4-phosphate; **II**: phosphoenolpyruvate; **III**: 7-phosphate-2-dehydro-3-deoxy-D-arabinoheptonate; **IV**: 3-dehydroquinate; **V**: 3-dehydroshikimate; **VI**: shikimate; **VII**: shikimate 3-phosphate; **VIII**: 5-O-(1-carboxyvinyl)-3-phosphoshikimate; **IX**: chorismate; **X**: anthranilate; **XI**: N-(5-Phospho-D-ribosyl)anthranilate; XII: 1-(2-carboxyphenylamino)-1-deoxy-D-ribulose 5-phosphate; **XIII**: indoleglycerol phosphate; **XIV**: indole; **XV**: tryptophan; **XVI**: prephenate; **XVII**: arogenate; **XVIII**: phenylpyruvate; **XIX**: 4-hydroxyphenylpyruvate; **XX**: tyrosine; **XXI**: phenylalanine. Enzymes – 2.5.1.54: 3-deoxy-7-phosphoheptulonate synthase; 4.2.3.4: 3-dehydroquinate synthase; 4.2.1.10: 3-dehydroquinate dehydratase I; 1.1.1.25: shikimate dehydrogenase; 2.7.1.71: shikimate kinase; 2.5.1.19: 3-phosphoshikimate 1-carboxyvinyltransferase; 4.2.3.5: chorismate synthase; 4.1.3.27: anthranilate synthase; 2.4.2.18: anthranilate phosphoribosyltransferase; 5.3.1.24: phosphoribosylanthranilate isomerase; 4.1.1.48: indoleglycerol phosphate synthetase; 4.2.1.20a/b: tryptophan synthase alpha (a) and beta (b) subunits; 4.2.1.51/5.4.99.5: bifunctional prephenate dehydratase/chorismate mutase; 2.6.1.57: aromatic amino acid aminotransferase; 1.3.1.13: prephenate dehydrogenase (NADP+); 2.6.1.1: aspartate aminotransferase; 2.6.1.5: tyrosine aminotransferase; 2.6.1.9: histidinol-phosphate aminotransferase; 1.14.16.1: phenylalanine-4-hydroxylase. Abbreviations as for Figure 1.

There are no enzymatic data concerning the synthesis of phenylalanine, tryptophan, and tyrosine in trypanosomatids. However, it is well known that these amino acids are essential in defined culture media designed for regular trypanosomatids, but not for symbiont-harboring ones [20,22,43,44]. The biosynthetic routes for these three amino acids use chorismate, which is produced from phosphoenolpyruvate (PEP) via the shikimate pathway, as a common substrate. The genomes of all endosymbionts contain the genes for this route, while the genomes of symbiont-harboring and regular trypanosomatids do not (Figure 5).

The genes for the enzymes converting chorismate into prephenate and for transforming this compound into phenylalanine and tyrosine are present in all endosymbiont genomes. Symbiont-harboring and regular trypanosomatid genomes also have the genes for the last step in the synthesis of phenylalanine and tyrosine, but it is not known whether all of these enzymes are functional. The gene for phenylalanine-4-hydroxylase (EC:1.14.16.1), which converts phenylalanine into tyrosine, is present in symbiont-harboring and regular trypanosomatids, including the leishmaniae, but not in endosymbionts. Similarly, this enzyme is present only in the aphid concerning the metabolic partnership between *Buchnera* and its insect host. Furthermore, the gene encoding this enzyme is up-regulated in bacteriocytes, thus enhancing the production and interconversion of such amino acids [71]. On the other hand, endosymbionts have an additional route for the synthesis of phenylalanine from prephenate, involving enzymes aromatic-amino-acid aminotransferase (EC:2.6.1.57) and prephenate dehydratase (EC:4.2.1.51), whose genes are absent in symbiont-harboring and regular trypanosomatid genomes.

The case of the last enzyme of the tryptophan pathway is rather interesting. Tryptophan synthase (EC:4.2.1.20) possesses two subunits. This bi-enzyme complex (a tetramer of two alpha and two beta subunits) channels the product of the alpha subunit (indole) to the beta subunit, which condenses indole and serine into tryptophan [73]. Both subunits are present in the endosymbionts, whereas the genomes of symbiont-harboring trypanosomatids and *H. muscarum* have only the beta subunit. None of the other trypanosomatid genomes examined presented either subunit of tryptophan synthase.

In summary, the endosymbionts have all the genes for the different routes leading from chorismate to tryptophan, tyrosine, and phenylalanine, which are absent from symbiont-harboring and regular trypanosomatid genomes. This obviously prevents regular trypanosomatids from synthesizing any of these three amino acids and growing without supplementation. It is worth observing that the presence of phenylalanine hydroxylase, which converts phenylalanine into tyrosine, in trypanosomatids but not in endosymbionts indicates that the host might control tyrosine production.

### Histidine

**Figure 6:**
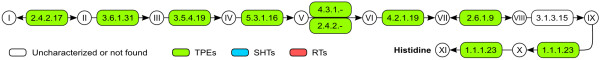
**Histidine synthesis pathway.** Enzymes surrounded by a thick gray box were shown to be horizontally transferred from Bacteria (see main text). Metabolites – **I**: 5-phosphoribosyl diphosphate (PRPP); **II**: phosphoribosyl-ATP; **III**: phosphoribosyl-AMP; **IV**: phosphoribosyl-formimino-AICAR phosphate; **V**: phosphoribulosyl-formimino-AICAR phosphate; **VI**: imidazole-glycerol 3-phosphate; **VII**: imidazole-acetol phosphate; **VIII**: histidinol phosphate; **IX**: histidinol; **X**: histidinal; **XI**: histidine. Enzymes – 2.4.2.17: ATP phosphoribosyltransferase; 3.6.1.31: phosphoribosyl-ATP pyrophosphohydrolase; 3.5.4.19: phosphoribosyl-AMP cyclohydrolase; 5.3.1.16: phosphoribosylformimino-5-aminoimidazole carboxamide ribotide isomerase; 4.1.3.-: cyclase HisF; 2.4.2.-: glutamine amidotransferase; 4.2.1.19: imidazole-glycerol phosphate dehydratase; 2.6.1.9: histidinol phosphate aminotransferase; 3.1.3.15: histidinol phosphatase; 1.1.1.23: histidinol dehydrogenase. Abbreviations as for Figure 1.

Histidine is derived from three precursors: the ATP purine ring furnishes a nitrogen and a carbon, the glutamine contributes with the second ring nitrogen, while PRPP donates five carbons. Histidine is a truly essential amino acid for most trypanosomatids, as corroborated by its obligatory presence in every synthetic media so far devised for regular trypanosomatid growth [22,43,44]. Accordingly, symbiont-harboring and regular trypanosomatid genomes do not seem to carry a single gene for histidine synthesis (Figure 6). All genes for the enzymes that participate in its biosynthesis, except the gene for histidinol-phosphate phosphatase (HPP, EC:3.1.3.15), which converts histidinol phosphate into histidinol, are present in the endosymbiont genomes. Since symbiont-harboring trypanosomatids do not require histidine, it is presumed that the absent EC:3.1.3.15 is replaced by an equivalent enzyme yet to be characterized (see Other observation on amino acid pathway peculiarities).

### Arginine and ornithine

**Figure 7:**
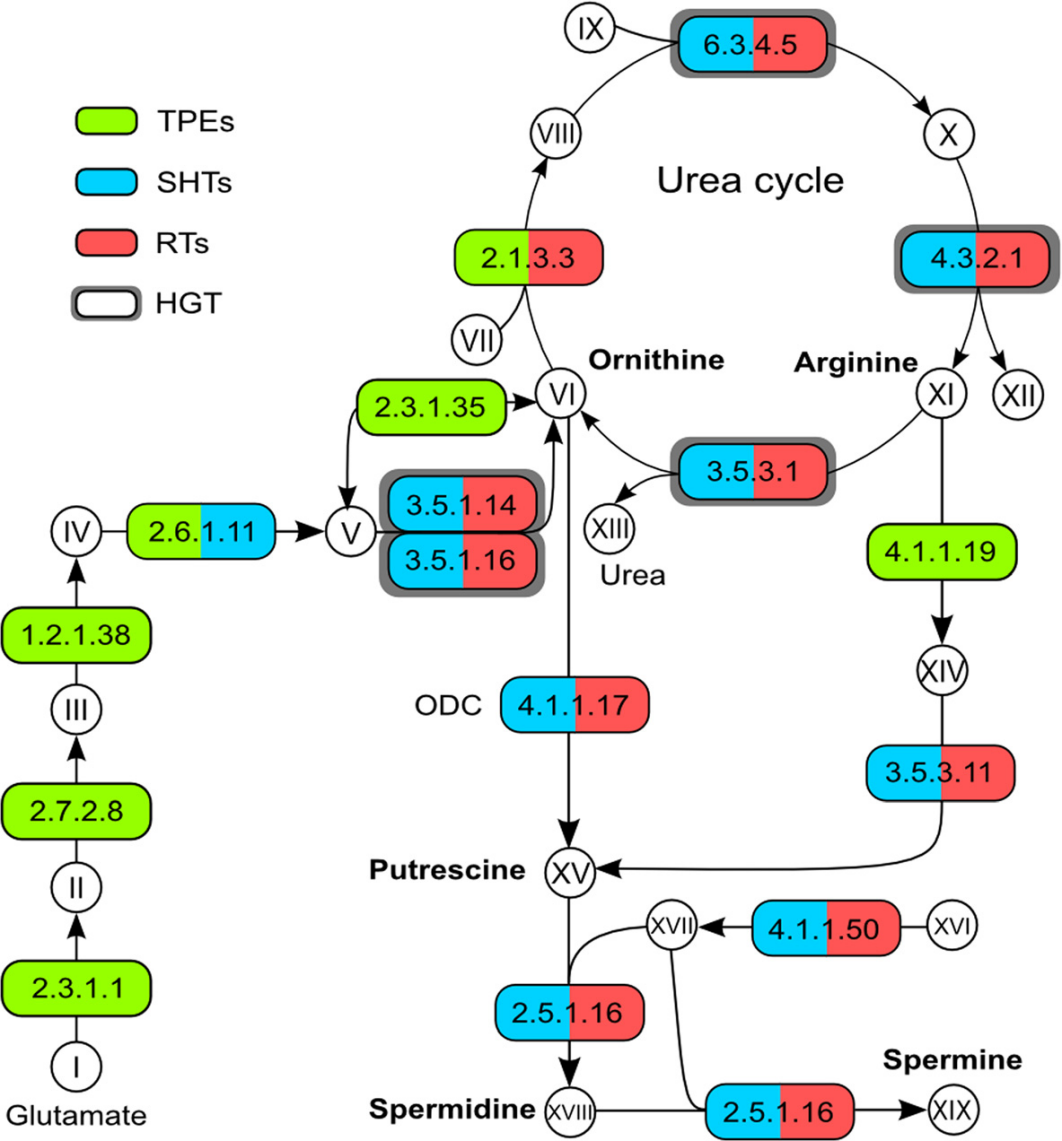
**Arginine, ornithine, and polyamine synthesis pathway.** Enzymes surrounded by a thick gray box were shown to be horizontally transferred from Bacteria (see main text). Metabolites – **I**: glutamate; **II**: N-acetylglutamate; **III**: N-acetylglutamyl-phosphate; **IV**: N-acetyl-glutamate semialdehyde; **V**: N-acetylornithine; **VI**: ornithine; **VII**: carbamoyl-phosphate; **VIII**: citruline; **IX**: aspartate; **X**: arginino succinate; **XI**: arginine; **XII**: fumarate; **XIII**: urea; **XIV**: agmatine; **XV**: putrescine; **XVI**: S-adenosylmethionine; **XVII**: S-adenosylmethioninamine; **XVIII**: spermidine; **XIX**: spermine. Enzymes – 2.1.3.3: ornithine carbamoyltransferase; 6.3.4.5: argininosuccinate synthase; 4.3.2.1: argininosuccinate lyase; 3.5.3.1: arginase; 4.1.1.17: ornithine decarboxylase; 3.5.1.14: aminoacylase; 3.5.1.16: acetylornithine deacetylase; 2.3.1.35: glutamate N-acetyltransferase; 2.6.1.11: acetylornithine aminotransferase; 1.2.1.38: N-acetyl-gamma-glutamyl-phosphate reductase; 2.7.2.8: acetylglutamate kinase; 2.3.1.1: amino-acid N-acetyltransferase; 3.5.3.11: agmatinase; 4.1.1.19: arginine decarboxylase; 4.1.1.50: adenosylmethionine decarboxylase; 2.5.1.16: spermidine synthase. Abbreviations as for Figure 1.

Organisms autotrophic for ornithine use the glutamate pathway [74] for its synthesis via acetylated compounds as represented in Figure 7 (I-VI). All genes for this pathway are present in the genomes of endosymbionts. The last step in the synthesis of ornithine can also be performed by the enzymes aminoacylase (EC:3.5.1.14) or acetylornithine deacetylase (EC:3.5.1.16), which convert acetylornithine into ornithine and are present in the genomes of symbiont-harboring and regular trypanosomatids, but not endosymbionts.

As represented in Figure 7, organisms lacking the glutamate pathway for the synthesis of ornithine can nevertheless produce it by different routes utilizing either citrulline or arginine [37,39,54]. Ornithine can be produced from the hydrolysis of citrulline mediated by citrulline hydrolase (EC:3.5.1.20). This activity is present in cell homogenates of all trypanosomatids, except the leishmaniae and trypanosomes, but the corresponding gene has not yet been identified to date in any organism, making it impossible to perform similarity searches. Ornithine can also be produced from arginine by means of arginase (EC:3.5.3.1), which splits arginine into ornithine and urea. The gene for arginase is present in the genomes of symbiont-harboring trypanosomatids and some regular trypanosomatids (*Leishmania* and *C. acanthocephali*), but not in the genomes of endosymbionts or *H. muscarum* – although a fragment was found in the later (see HGT and arginine and ornithine biosynthesis).

Arginine can be synthesized from ornithine through a recognized universal enzymatic pathway [74], the first step of which is the conversion of ornithine and carbamoyl phosphate into citrulline mediated by OCT (ornithine carbamoyl transferase, EC:2.1.3.3). The gene for OCT was found in the genomes of all endosymbionts and also in *Herpetomonas*, but was absent from other regular, as well as symbiont-harboring, trypanosomatid genomes examined. These findings confirm earlier immunocytochemical ultrastructural experiments showing the presence of OCT in the symbiont of *Angomonas deanei*[36]*.* The absence of the OCT gene renders most trypanosomatids unable to make citrulline from ornithine [75]. However, the genes for the remaining enzymes leading from citrulline into arginine are all present in the genomes of all regular and symbiont-harboring trypanosomatids, but absent from the endosymbiont genomes. These data are in full accordance with earlier enzymatic determinations for argininosuccinate synthase (EC:6.3.4.5), argininosuccinate lyase (EC:4.3.2.1), and arginase (EC:3.5.3.1) in cell homogenates of trypanosomatids [38,39,56].

Taking all these data together, we can conclude that regular trypanosomatids require exogenous sources of arginine or citrulline in their culture medium to produce ornithine. This is related to the fact that regular trypanosomatids lack the glutamate pathway for ornithine synthesis. Furthermore, ornithine cannot substitute for arginine or citrulline because most regular trypanosomatids lack OCT. Conversely, symbiont-harboring trypanosomatids are autotrophic for ornithine. This is due to the fact that, although the symbiont lacks most genes for ornithine production, it contains sequences for key enzymes such as those for the glutamate route and OCT, which converts ornithine into citrulline thus completing the urea cycle.

### Polyamines

As shown in Figure 7, putrescine, a polyamine associated with cell proliferation, can be produced from ornithine in a one-step reaction mediated by ODC (ornithine decarboxylase, EC:4.1.1.17), whose gene is present in the genomes from the genus *Angomonas* and regular trypanosomatids, but not in endosymbionts or *Strigomonas*. Interestingly, it was proposed that the symbiont can enhance the ODC activity of *A. deanei* by producing protein factors that increase the production of polyamines in the host trypanosomatid [76]. Such high ODC activity may be directly connected to the lowest generation time described for trypanosomatids that is equivalent to 6 hours [13]. Putrescine could also be produced from agmatine since the genomes of regular and symbiont-harboring trypanosomatids have the gene for agmatinase (EC:3.5.3.11), converting agmatine into putrescine. However, the gene for the enzyme arginine decarboxylase (EC:4.1.1.19), which synthesizes agmatine, is present solely in the genomes of endosymbionts, thus completing the biosynthetic route for this polyamine, via agmatinase, in symbiont-harboring trypanosomatids. Putrescine is then converted to spermidine and spermine by enzymes S-adenosylmethionine decarboxylase (EC:4.1.1.50) and spermidine synthase (EC:2.5.1.16). The genes for these enzymes are present in the regular and symbiont-harboring trypanosomatids, but not in endosymbionts (Figure 7). Enzyme EC:2.5.1.16, converting S-adenosylmethioninamine and putrescine into S-methyl-5’-thioadenosine and spermidine, also participates in a reaction from the methionine salvage pathway. This pathway is present, complete in all symbiont-harboring and regular trypanosomatids examined (Additional file 3), although there are questions regarding the step catalyzed by acireductone synthase (EC:3.1.3.77, see HGT and methionine and cysteine biosynthesis).

### Phylogenetic analyses

**Figure 8:**
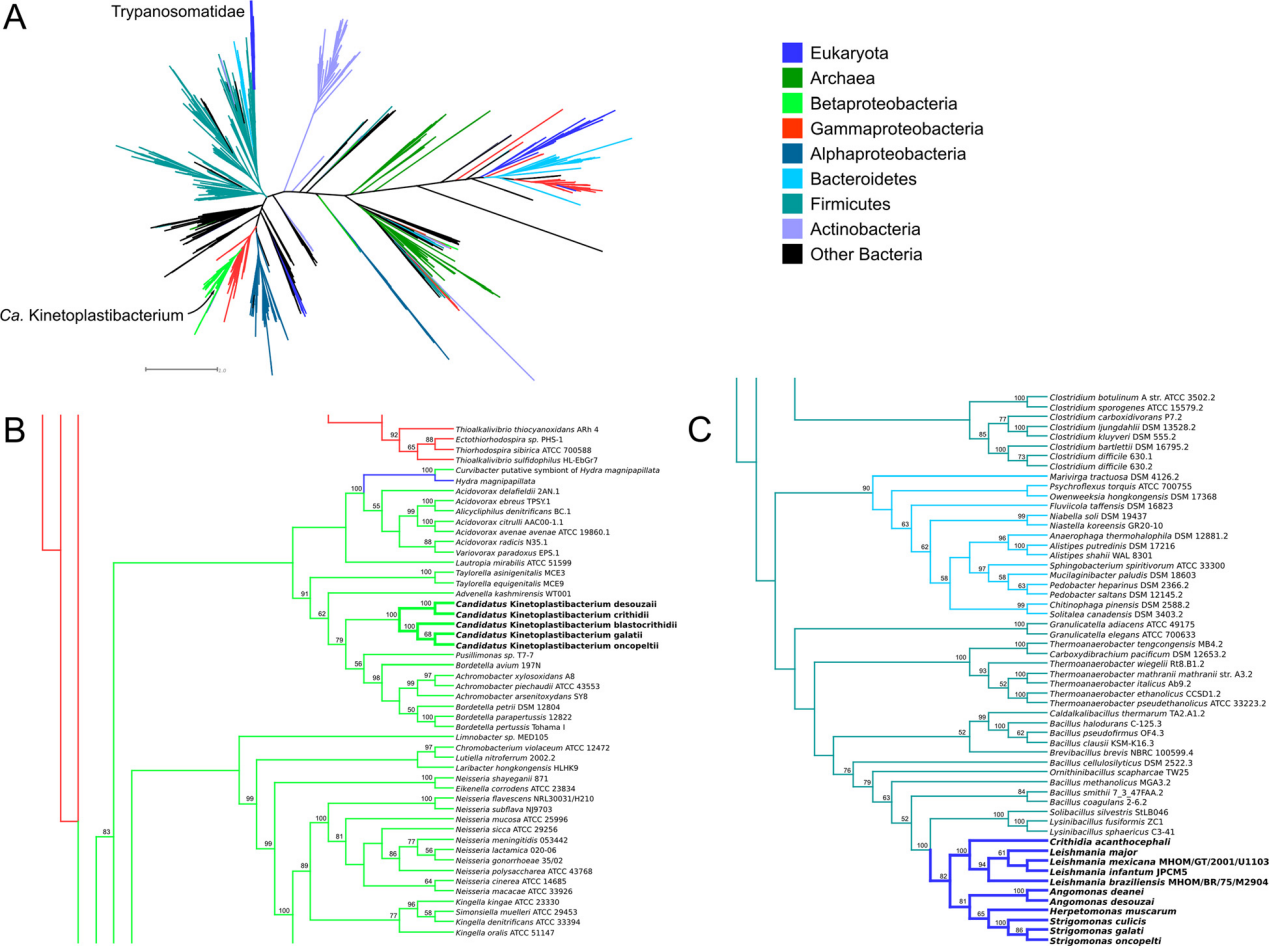
**Maximum likelihood phylogenetic tree of homoserine dehydrogenase (EC:1.1.1.3). A** – overall tree, colored according to taxonomic affiliation of each taxon, as per the legend on the right; distance bar only applies to panel **A**. **B** – details of the region of the tree where the *Ca.* Kinetoplastibacterium spp. are placed. **C** – details of the region of the tree where the Trypanosomatidae are placed. Values on nodes represent bootstrap support (only 50 or greater shown). Panels **B** and **C** are meant to only represent the branching patterns and do not portray estimated distances between sequences. Abbreviations as for Figure 1.

Our data on the phylogeny of the genes for essential amino acid biosynthesis have clearly shown that the genes present in the symbionts are of betaproteobacterial origin (for an illustrative example, see Figure 8), as shown before for the genes of heme synthesis [58] and many others across the endosymbiont genomes [25]. The symbiont-harboring and regular trypanosomatid genomes, on the other hand, present a rather different situation. Thus, 18 of the 39 genes required for the biosynthesis of essential amino acids exhibited at least some phylogenetic evidence of having been horizontally transferred from a bacterial group to a trypanosomatid group, with three other genes presenting undetermined affiliation (see Additional file 2 for a summary of the phylogenetic analyses results). As detailed below, horizontal gene transfer (HGT) events seem to have originated from a few different bacterial taxa, although in some cases the exact relationship was not completely clear. Also, while some transfers are common to all trypanosomatid groups examined, others were found to be specific to certain subgroups. This could be due to multiple HGT events from associated bacteria at different points of the family’s evolutionary history or, alternatively, to HGT events that occurred in the common ancestor of all trypanosomatids, whose corresponding genes were later differentially lost in certain taxa. Given the low number of genomes currently known in the family, it is difficult to assign greater probability to either scenario.

Regarding the taxonomic affiliation of the putative origin of these HGT events, it is possible to notice a preponderance of bacteria from a few phyla with three or more genes transferred, i.e. Firmicutes, Bacteroidetes, and Gammaproteobacteria, plus a few other phyla with two or less genes represented, like Actinobacteria, Betaproteobacteria, Acidobacteria, and Alphaproteobacteria. In a few other cases, the trypanosomatid genes grouped inside diverse bacterial phyla, in which case the assignment of a definite originating phylum was not possible. However, given the sometimes high rate of HGT in prokaryotic groups, it is difficult to assess with confidence the correct number of putative HGT events from Bacteria to Trypanosomatidae. It is possible that some of the genes that seem to have originated from different phyla could actually have come from one bacterial line that was itself the recipient of one or more previous HGT events from other bacteria.

Analysis of all generated phylogenetic inferences has uncovered a clear pattern for the HGT events, which were shown to be concentrated preferentially in pathways or enzymatic steps that are usually reported to be absent in eukaryotes, particularly animals and fungi. Thus, the HGT events identified in this study involve pathways for the synthesis of lysine, cysteine, methionine, threonine, tryptophan, ornithine, and arginine (Figures 1, 2, 3, 5, and 7) and also the synthesis of a few non-essential amino acids such as glycine, serine, and proline. The detailed analysis of these events in different genes and pathways follows.

### HGT of homoserine dehydrogenase

Some enzymes are common to a number of pathways involving key precursors to many compounds. Homoserine dehydrogenase (EC:1.1.1.3), for example, participates in the aspartate semialdehyde pathway for the synthesis of lysine, cysteine, methionine, and threonine (Figures 1, 2, and 3). The gene for EC:1.1.1.3 present in symbiont-harboring and regular trypanosomatid genomes seems to have been transferred from a member of the Firmicutes, clustering most closely with *Solibacillus silvestris*, *Lysinibacillus fusiformis*, and *L. sphaericus* with bootstrap support value (BSV) of 100 (Figure 8). On the other hand, the endosymbiont ortholog groups deep within the Betaproteobacteria, more specifically in the Alcaligenaceae family, as expected in the case of no HGT of this gene into the endosymbiont genomes.

### HGT and lysine biosynthesis

The two genes of the lysine pathway (Figure 1) that were found in trypanosomatid genomes presented evidence of HGT. *H. muscarum* was the only trypanosomatid analyzed containing the next to last gene, for diaminopimelate epimerase (EC:5.1.1.7), which clusters strongly with the phylum Bacteroidetes, with BSV of 99 (Additional file 4). The last gene, for diaminopimelate decarboxylase (EC:4.1.1.20), was present in the symbiont-harboring and regular trypanosomatids. In the phylogeny, this particular gene has Actinobacteria as sister group (BSV of 79), although also grouping with a few other eukaryotic genera, most closely *Dictyostelium*, *Polysphondylium*, and *Capsaspora*, with BSV of 65 (Additional file 5). There are, overall, very few Eukaryota in the tree for 4.1.1.20, making it hard to reach a definite conclusion on the direction of transfer for this gene, since other eukaryotes are also present basally to this substantially large group of Actinobacteria plus Trypanosomatidae, with the high BV of 98.

Using the *C. acanthocephali* gene for EC:4.1.1.20 in a manual search against the *L. major* genome has shown a small fragment with significant similarity (57% identity and 67% similarity, from amino acid 177 to 227), but containing stop codons. Search against predicted *L. major* proteins yielded no results. These sequence remains suggest that *Leishmania* could have lost DAP-decarboxylase in a relatively recent past.

### HGT and methionine and cysteine biosynthesis

The pathways for cysteine and methionine synthesis (Figure 2) present the highest number of HGT events identified among the pathways studied here. The gene for the enzyme EC:2.3.1.30, necessary for the conversion of serine to cysteine, seems to have been transferred from Bacteria to the genomes of host trypanosomatids. EC:2.3.1.30 of symbiont-harboring and regular trypanosomatids grouped inside a large cluster of diverse Bacteria (predominantly Bacteroidetes and Betaproteobacteria), with BSV of 80 (Additional file 6). An even deeper branch, which separates the subtree containing the trypanosomatids from the rest of the tree, has BSV of 97. The evolutionary history of the other enzyme with the same functionality, EC:2.5.1.47, is unclear and can not be considered a case of HGT given current results. Its gene is present in symbiont-harboring and regular trypanosomatids (including one sequence from *T. cruzi* CL Brener) and clusters as a sister group of Actinobacteria, although with low BSV (Additional file 7). Although there are many other eukaryotes in the tree, they are not particularly close to the subtree containing the Trypanosomatidae. Interestingly, one *Entamoeba dispar* sequence is a sister group to the Trypanosomatidae, although with low BSV, raising the possibility of eukaryote-to-eukaryote HGT, as previously reported (reviewed in [77]).

The gene for EC:2.3.1.46, the first in the pathway converting homoserine to cystathionine, is present in all symbiont-harboring trypanosomatids and *Herpetomonas*, but in no other regular trypanosomatid examined. This trypanosomatid gene groups within Bacteroidetes, with BSV of 53 and, in a deeper branch, BSV of 89, still clustering with Bacteroidetes only (Additional file 8).

The gene for EC:2.1.1.37, responsible for the first step in the conversion of S-adenosylmethionine into homocysteine, is present in all symbiont-harboring and regular trypanosomatids, although the sequence is still partial in the genome sequences of the *Angomonas* species. Almost all organisms in the tree are Bacteria of several different phyla (Additional file 9), with the few Eukaryota present forming a weakly supported clade. KEGG shows that many Eukaryota do possess a gene for enzyme EC:2.1.1.37, but their sequences are very different from that present in the trypanosomatids (and other eukaryotes) studied here. This therefore suggests a bacterial origin for the EC:2.1.1.37 from the Eukaryota in our tree, although the specific donor group cannot be currently determined with confidence. It is interesting to note that, besides the Trypanosomatidae, the clade of eukaryotes is composed of Stramenopiles and green algae (both groups that have, or once had, plastids), with a Cyanobacteria close to the base of the group. Although the BSV of 54 does not allow strong conclusions regarding this group, it is interesting to speculate about the possibility of eukaryote-to-eukaryote gene transfer, as previously seen (reviewed in [77]), after the acquisition of this gene from a so-far unidentified bacterium.

The genes for EC:2.5.1.48, EC:2.5.1.49, and EC:4.4.1.8 (two versions) are quite similar in sequence and domain composition. Therefore, similarity searches with any one of these genes also retrieves the other three. In spite of the similarities, these genes are found in rather different phyletic and phylogenetic patterns on the trypanosomatids (Additional file 10). EC:2.5.1.48 is present in all symbiont-harboring and regular trypanosomatids examined, plus *Trypanosoma* sp. and a few other Eukaryota (mostly Apicomplexa and Stramenopiles), all within a group of Acidobacteria (BSV of 94). The gene for EC:2.5.1.49 is present in the symbiont-harboring trypanosomatids and *Herpetomonas*, but in none of the other regular trypanosomatids examined. This trypanosomatid gene also clusters with diverse groups of Bacteria, although low BSV makes it hard to confidently identify its most likely nearest neighbor, and it is not possible to conclude with reasonable certainty that this gene is derived from HGT. The gene for EC:4.4.1.8 occurs, in symbiont-harboring and regular trypanosomatids, as two orthologs presenting very different evolutionary histories. One of the orthologs clusters with eukaryotes, with BSV of 95, while the other seems to be of bacterial descent, grouping mostly with Alphaproteobacteria of the Rhizobiales order, with BSV of 99.

The presence of two genes identified as EC:4.4.1.8 raises the possibility of them performing different enzymatic reactions. Given the overall domain composition similarities of several of the genes of the methionine and cysteine synthesis pathways, it is possible that one of the enzymes identified as EC:4.4.1.8 is actually the enzyme EC:4.4.1.1, for which no gene has been found in our searches of the Trypanosomatidae genomes, as detailed above (*Methionine and cysteine*).

Genes for two of the enzymes for the last step in the methionine synthesis, EC:2.1.1.10 and EC:2.1.1.14 (Additional files 11 and 12), are present in all regular and symbiont-harboring trypanosomatids (except for *Herpetomonas*, which lacks the latter). EC:2.1.1.14 appears to be of bacterial origin, grouping within the Gammaproteobacteria with moderate (74) bootstrap support. While EC:2.1.1.10 also groups near Gammaproteobacteria, BSV is low and this gene cannot be considered a case of HGT given current data.

As seen above, most genes in the *de novo* methionine synthesis pathway seem to have originated in one or more HGT events. Enzymes from the methionine salvage pathway (Additional file 3), on the other hand, are notably different. Of these, only S-methyl-5-thioribose kinase (EC:2.7.1.100), found in *C. acanthocephali* and *Herpetomonas* but not in the endosymbionts and symbiont-harboring trypanosomatids, seems to have originated in a bacterial group (Additional file 13). These two organisms’ enzymes group deep within the Gammaproteobacteria, with BSV of 97.

Enzyme acireductone synthase (EC:3.1.3.77) presents an intriguing case, being the only methionine salvage pathway enzyme absent from the symbiont-harboring trypanosomatid genomes. This enzyme is of eukaryotic origin (not shown), and present in both *H. muscarum* and *C. acanthocephali*, but was not found in any other of the regular trypanosomatids available from KEGG. Interestingly, KEGG data for *Trypanosoma brucei* also shows the two enzymes preceding EC:3.1.3.77 as missing, which raises the question of whether this important pathway is in the process of being lost in trypanosomatids. If that is not the case, and given that all other enzymes from the pathway are present, the Trypanosomatidae must have a different enzyme (or enzymes) to perform the required reactions.

### HGT and threonine biosynthesis

The gene for the enzyme that interconverts glycine and threonine (Figure 3), EC:4.1.2.5, was identified in all symbiont-harboring and regular trypanosomatids (except *Herpetomonas*), but the evolutionary histories of symbiont-harboring and regular trypanosomatid genes are very different (Additional file 14). The gene found in the regular trypanosomatids *Leishmania* sp. and *C. acanthocephali* groups deep within the Firmicutes, most closely *Clostridium*, with BSV of 63. The symbiont-harboring trypanosomatid genes, on the other hand, cluster as the most basal clade of one of the two large assemblages of eukaryotes present in this phylogeny, although all BSV are low and there is a large group of Bacteria from diverse phyla between the symbiont-harboring trypanosomatids plus a few other eukaryotic groups and the other eukaryotes in this part of the tree. It is therefore difficult to conclude whether the symbiont-harboring trypanosomatid gene is of bacterial or eukaryotic origin.

### HGT and tryptophan biosynthesis

Tryptophan synthase beta subunit (EC:4.2.1.20), present in the symbiont-harboring trypanosomatids and *Herpetomonas*, is the last enzyme of the tryptophan biosynthesis pathway, and the only one present in trypanosomatids for this pathway. Its gene groups robustly (BSV of 97) with the Bacteroidetes phylum (Additional file 15). It is also highly similar (around 80% identity and 90% similarity) to the corresponding genes of this phylum, suggesting either a very recent transfer or high sequence conservation. Given that the protein alignment of the orthologs (not shown) presents a maximum patristic distance value of 84.04% and a median of 47.22%, it is therefore likely that the transfer of EC:4.2.1.20 to the Trypanosomatidae is relatively recent.

### HGT and arginine and ornithine biosynthesis

The arginine and ornithine synthesis pathway has been influenced by HGT events in a few key steps. As discussed above, one of the entry points for the urea cycle is through ornithine synthesized from glutamate. The last step, converting N-acetylornithine to ornithine, can be performed by either EC:3.5.1.14 or EC:3.5.1.16 (Figure 7). We have found that the genes for both enzymes, present in all symbiont-harboring and regular trypanosomatid genomes, originated from HGT events. All gene copies for EC:3.5.1.14 group as one clade with a gammaproteobacterium (BSV of 98), and with Bacteria of different phyla (predominantly Firmicutes) as nearest sister group, although with low BSV (Additional file 16). The few other eukaryotic groups present in the tree are very distant from the trypanosomatid group. The multiple copies of the gene for EC:3.5.1.16 in symbiont-harboring and regular trypanosomatids group together in a monophyletic clade (Additional file 17), which clusters within a large group of mostly Betaproteobacteria with BSV of 80, including the Alcaligenaceae, the family to which the endosymbionts belong. However, it seems highly unlikely that this sequence has been transferred from the endosymbiont genomes to their hosts genomes because the nuclear sequences are firmly removed from the Alcaligenaceae, and many regular trypanosomatids (including *Trypanosoma* spp.) also present this gene in the same part of the tree.

The only trypanosomatid analyzed which presented ornithine carbamoyl transferase (OCT, EC:2.1.3.3) was *Herpetomonas muscarum*. Our phylogenetic analysis of this gene indicates that it is of eukaryotic origin (not shown). The symbiont-harboring trypanosomatids utilize the OCT provided by their endosymbionts, and their OCT genes group firmly inside the Alcaligenaceae family, next to *Taylorella* and *Advenella*, as expected.

The genes for EC:6.3.4.5 and EC:4.3.2.1 present similar evolutionary patterns: both are absent from endosymbiont genomes and present in all symbiont-harboring and regular trypanosomatid genomes – the only exception being the lack of the latter in *Leishmania* spp. The Trypanosomatid genes form monophyletic groups in their respective trees, grouping within Firmicutes in both cases (Additional files 18 and 19). BSV is higher (82) in the tree of EC:4.3.2.1 than in that of EC:6.3.4.5 (69). In both cases, support falls for deeper branches in the trees. Although the host genomic sequences are still incomplete and in varying degrees of contiguity, it is interesting to note that the genes for EC:6.3.4.5 and EC:4.3.2.1 are present in tandem in one contig in all symbiont-harboring trypanosomatids (Additional file 1). The flanking genes are eukaryotic: terbinafine resistance locus protein and a multidrug resistance ABC transporter. As seen in the genome browser at TriTrypDB (http://tritrypdb.org), *Leishmania* spp. have most of these same genes, although in a slightly different order (EC:6.3.4.5 occurring after the two eukaryotic genes instead of between them) and lacking EC:4.3.2.1. *L. braziliensis* seems to be in the process of additionally losing EC:6.3.4.5, which is annotated as a pseudogene. These phylogenetic and genomic data strongly suggest that EC:4.3.2.1 and EC:6.3.4.5 have been transferred together from a Firmicutes bacterium to the common ancestor of the symbiont-harboring and regular trypanosomatids studied, and that these transferred genes have been or are being lost from *Leishmania* at least.

The final enzyme in the urea cycle, arginase (EC:3.5.3.1), is present in all symbiont-harboring and regular trypanosomatids examined here. However, the sequence from *Herpetomonas* presents a partial arginase domain; while the protein sequence length is as expected, the domain match starts only after 70 amino acids. We speculate that this divergence could be responsible for the lack of arginase activity previously seen in *Herpetomonas*. Differently from most other enzymes in this work, there are different evolutionary histories for the arginase genes: all trypanosomatid genes but that from *Herpetomonas* cluster together with very high bootstrap support of 98, within Eukaryota (Additional file 20). The sequence from *Herpetomonas* on the other hand is the sister group (BSV of 79) of a large assemblage of Bacteria from several different phyla, but predominantly Deltaproteobacteria, Firmicutes, Actinobacteria, and Cyanobacteria. It is therefore clear that *Herpetomonas* must have acquired a different arginase than that present in the other trypanosomatids studied, which possess eukaryotic genes. Furthermore, this gene seems to be undergoing a process of decay, given its lack of significant similarity to the known arginase domain in a significant portion of the protein.

### HGT in other pathways: possible symbiont to host transfer

Ornithine cyclodeaminase (EC:4.3.1.12) converts ornithine directly into proline, a non-essential amino acid. In our analyses, we have found that the gene for EC:4.3.1.12 of symbiont-harboring trypanosomatid genomes is very similar to those from Betaproteobacteria of the Alcaligenaceae family, to which the endosymbionts belong. Regular trypanosomatid and endosymbiont genomes do not contain the gene for this enzyme. Accordingly, the phylogeny shows the symbiont-harboring trypanosomatid gene grouping close to several Alcaligenaceae, although the clade is not monophyletic and presents BSV of 63 (Additional file 21). This grouping, together with the gene presence in symbiont-harboring trypanosomatid genomes only, poses the possibility that EC:4.3.1.12 has been transferred from the ancestral endosymbiont to the corresponding host, before the radiation of symbiont-harboring trypanosomatids into the two genera and five species analyzed here.

### Other observation on amino acid pathway peculiarities

Some interesting peculiarities of specific genes from a few pathways deserve to be discussed. Interestingly, the gene for branched-chain-amino-acid transaminase (EC:2.6.1.42), the last step in the synthesis of isoleucine, valine, and leucine (Figure 4), was identified in all bacteria of the Alcaligenaceae family present in KEGG, except for the endosymbionts’ closest relatives, *Taylorella* spp. (parasitic) and *Advenella kashmirensis* (free-living), which also lack the gene. The question is raised then of whether the common ancestor of *Taylorella* and the endosymbionts, which are sister groups [25], had already lost the gene. Another possibility is that independent loses occurred in endosymbionts, *Taylorella*, and *Advenella*. Considering that the rest of the pathway is present in these organisms and that the free-living *Advenella* would need the last gene to complete synthesis of these amino acids, it is reasonable to speculate that their EC:2.6.1.42 is novel or at least very different and thus could not be identified by similarity searches.

As mentioned above, the histidine pathway biosynthesis is performed by the endosymbionts and all enzymes, with the exception of histidinol-phosphate phosphatase (HPP, EC:3.1.3.15), have been identified. This is also the only enzyme of this pathway missing in other Betaproteobacteria available in KEGG. Recently, it was reported that such a gap in the histidine biosynthesis pathway in other organisms was completed by novel HPP families [78,79]. Our searches for the novel *C. glutamicum* HPP (cg0910, an inositol monophosphatase-like gene) have identified two possible candidate genes in the endosymbionts (BCUE_0333 and BCUE_0385, in *C.* K. blastocrithidii). As in *Corynebacterium*, neither of these genes is in the same operon as the known histidine synthesis genes. Given the absence of any other inositol phosphate metabolism genes in the endosymbiont genomes, except for these two IMPases, it is reasonable to hypothesize that at least one of the two aforementioned candidates could be the HPP.

## Conclusions

**Figure 9:**
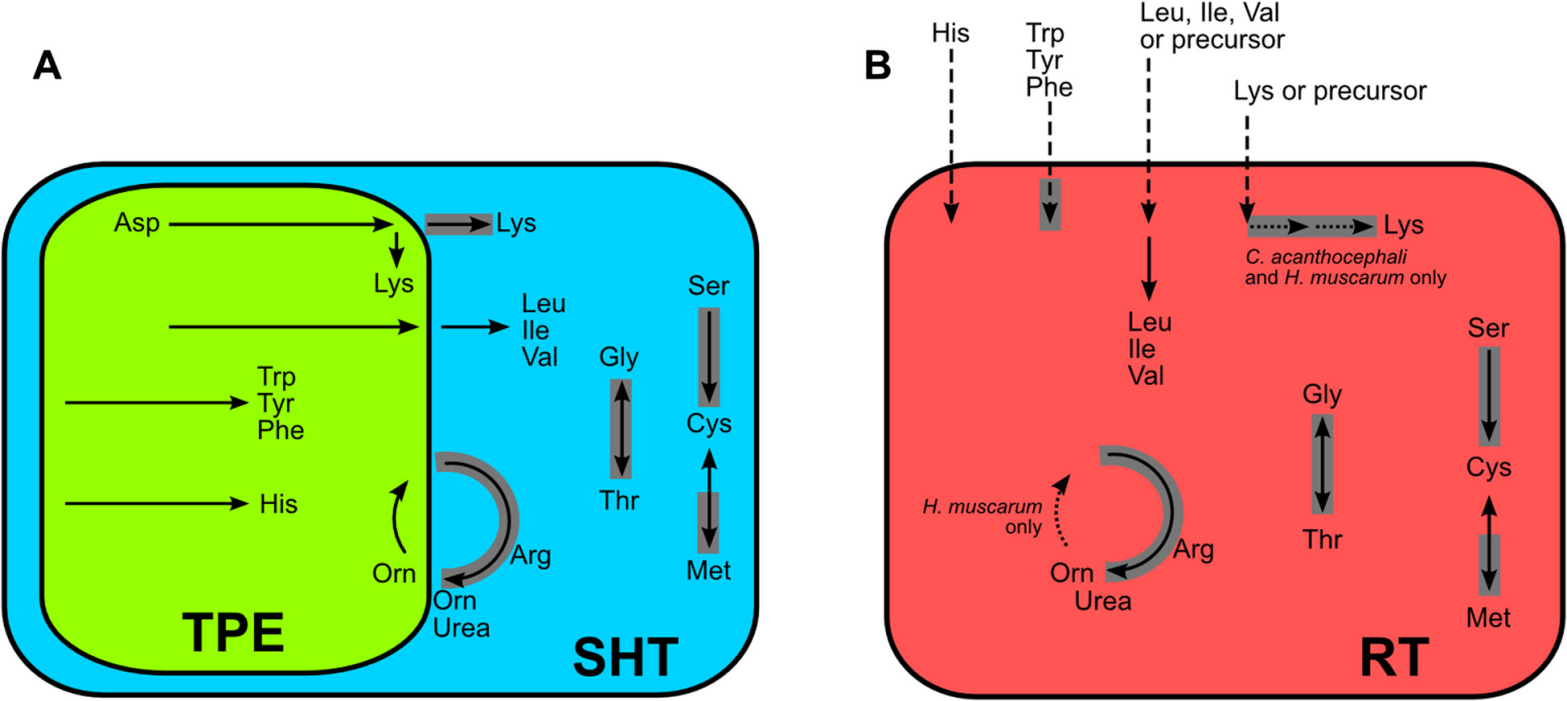
**Overview of the biosynthetic pathways of essential amino acids in trypanosomatids.** Dashed arrows: metabolite import; dotted arrows: reaction present in only some of the organisms analyzed; solid arrows: other reactions (a single arrow can summarize multiple steps); arrows surrounded by a gray box: enzymes possibly acquired through horizontal transfer from Bacteria to trypanosomatids (see main text). **A**. Contribution of symbiont-harboring trypanosomatids (SHT) and endosymbionts (TPE) based on the analysis of gene content in the genomes of *A. deanei*, *A. desouzai*, *S. culicis*, *S. oncopelti*, *S. galati*, and respective endosymbionts. **B**. Biochemical capability of trypanosomatids without symbionts (RT), based on the analysis of genomic data from *H. muscarum*, *C. acanthocephali*, and *L. major*.

In the present paper, we have put together nutritional, biochemical, and genomic data in order to describe how the metabolic co-evolution between the symbiont and the host trypanosomatid is reflected in amino acid production (Figure 9). In fact, amino acid biosynthetic pathways in symbiont-harboring trypanosomatids are frequently chimeras of host and endosymbiont encoded enzymes, with predominance of the latter in the synthesis of essential amino acids. After a careful analysis of different routes, it becomes clear that the symbiotic bacterium completes and/or potentiates most pathways of the host protozoa that are involved in amino acid production, as previously seen in other systems [7].

Sometimes, as in the lysine and histidine synthesis, the symbionts contain all genes for enzymes that compose the metabolic route. By contrast, in the cysteine and methionine pathways the bacterium lacks most genes involved in amino acid interconversion, which are present in host trypanosomatids. Interestingly, the last step of some metabolic routes such as those for lysine and tryptophan, contains two genes; one in the host genome, the other in the endosymbiont genome. This phenomenon has also been observed in the synthesis of heme [58,80], but the reasons for this peculiarity remain obscure. However, we have to consider the possibility that HGT events preceded the colonization of symbiont-harboring trypanosomatids by their endosymbionts, and that the genes present in the host genomes are just relics of previous HGT event(s). Alternatively, these genes could have been recruited to perform functions, as the control of amino acid production by the host trypanosomatid. This same strategy can be considered in isoleucine, valine, and leucine production, but in this case endosymbionts lack the enzyme for the last step, the branched-chain amino acid transaminase (EC:2.6.1.42).

A clear example of the integration of earlier nutritional and enzymatic data with the present gene screening is the synthesis of arginine and ornithine in trypanosomatids. Differently from other members of the family, the urea cycle is complete in symbiont-harboring trypanosomatids by the presence of the OCT gene (EC:2.1.3.3) in symbionts, making these protozoa entirely autotrophic for ornithine, citrulline, and arginine, as previously known from nutritional data [19,22,44,52]. Symbiont-bearing trypanosomatids contain genes for all enzymes leading from glutamate to arginine. The corresponding genes are located partly in the genomes of their endosymbionts and partly in the protozoan nucleus; in this last case, genes are of bacterial origin, resulting from HGT and including at least one transfer of two genes at once (EC:4.3.2.1 and EC:6.3.4.5), as demonstrated in our phylogenies. Furthermore, endosymbionts also contain most genes for the glutamate pathway, thus enhancing synthesis of ornithine, that once decarboxylated generates polyamine, which is related to cell proliferation and to the low generation time displayed by symbiont-harboring trypanosomatids. Results in this study confirm previous findings [25,58] showing the betaproteobacterial origin of the genes of endosymbionts. The nuclear genes, on the other hand, present a much more convoluted evolutionary picture, with probably numerous ancient HGT events shaping the amino acid metabolism in trypanosomatids. A few pathways in particular have been heavily affected, i.e. methionine/cysteine and arginine/ornithine synthesis. Transferred genes originated preferentially from three bacterial phyla, namely Firmicutes, Bacteroidetes, and Gammaproteobacteria, although possible transfers from other phyla of Bacteria have also been uncovered. Especially interesting was the finding of a gene, coding for ornithine cyclodeaminase (EC:4.3.1.12), which closely groups with the Alcaligenaceae family of the Betaprotebacteria and that is likely to have been transferred from the endosymbiont to the host genome. Accordingly, it is present only in symbiont-harboring trypanosomatid nuclear genomes and not in any of the currently sequenced regular trypanosomatid genomes. During review of this work, a very recent report of a similar situation of multiple lineages contributing to the metabolism in the symbiosis of mealybugs, involving the three interacting partners and genes acquired through HGT from other bacterial sources (mainly Alphaproteobacteria, but also Gammaproteobacteria and Bacteroidetes) to the insect host, has been published [81]. This suggests that this phenomenon could be widespread and of great importance in genomic and metabolic evolution.

Having been detected in about half of the genes analyzed in this work, HGT events seem to have been fundamental in the genomic evolution of the Trypanosomatidae analyzed, and further phylogenetic studies of the whole host genomes should show the complete extent of this process and which additional pathways could be affected. Synthesis of vitamins (Klein et al., personal communication), heme, and amino acids have already been shown to benefit from bacterial-to-trypanosomatid HGT; many other processes in Trypanosomatidae metabolism might also be subjected to this evolutionary process.

## Methods

### Organisms and growth conditions

The symbiont-harboring trypanosomatid species genomes sequenced here were: *Strigomonas oncopelti* TCC290E, *S. culicis* TCC012E, *S. galati* TCC219, *Angomonas deanei* TCC036E, and *A. desouzai* TCC079E. These symbiont-harboring trypanosomatids harbor, respectively, the symbionts: *Candidatus* Kinetoplastibacterium oncopeltii, *Ca*. K. blastocrithidii, *Ca*. K. galatii, *Ca*. K. crithidii and *Ca*. K. desouzaii [24], and were previously sequenced [25]. In addition, we have also sequenced the genomes of two regular trypanosomatids, i.e. *Herpetomonas muscarum* TCC001E and *Crithidia acanthocephali* TCC037E. These organisms are cryopreserved at the Trypanosomatid Culture Collection of the University of São Paulo, TCC-USP. Symbiont-harboring trypanosomatids were grown in Graces’ medium (Gibco). Regular trypanosomatids were grown in LIT media [82].

### DNA extraction and sequencing

Total genomic DNA was extracted by the phenol-chloroform method [83]. We applied kDNA depletion methods to minimize the presence of this type of molecule, as previously described [58], which result in less than about 5% of remaining kDNA in the sample. After kDNA depletion, about 5 μg of DNA were submitted to each Roche 454 shotgun sequencing run, according to the manufacturer’s protocols. Different genomes have so far been sequenced to different levels of draft quality, with estimated coverages of 15X to 23X (considering a genome of ~30 Mbp). Sequences were assembled using the Newbler assembler version 2.3, provided by Roche. Resulting assemblies are available from GenBank, as detailed in “Availability of Supporting Data” below. Endosymbiont genomes were finished to a closed circle as previously described [25].

### Gene discovery and annotation

Endosymbiont genes were used as previously published [25]. In an initial scan of the genome, trypanosomatid genes were discovered and mapped to metabolic pathways using ASGARD [84], employing as reference the UniRef100 [85] and the Kyoto Encyclopedia of Genes and Genomes, KEGG [86] databases. The identified segments of DNA were then extracted from the genome and manually curated for completion and proper location of start and stop codons by using the GBrowse genome browser [87]. Putative sequence functions were confirmed by domain searches against NCBI’s Conserved Domain Database [88]. Genes and annotations from other trypanosomatids were used when needed and as available at KEGG. All trypanosomatid genes characterized in this study have been submitted to NCBI’s GenBank and accession numbers are available from Additional file 22. All endosymbiont genes analyzed here have been previously sequenced [25]; gene identifiers are available from Additional file 23.

Due to the incomplete nature of our trypanosomatid assemblies, a set of criteria were used to avoid including contaminant sequences in our analyses. A gene was accepted as legitimate only when satisfying at least two of the following: genomic context compatible with a trypanosomatid gene (i.e. long stretches of genes in the same orientation in the contig, most neighboring genes similar to genes from other, previously sequenced trypanosomatids); sequencing coverage in the gene similar to, or higher than, that of the gene and genome averages (since contaminants that are difficult to detect will almost always be in small contigs of low coverage); GC percent content consistent with that of the neighboring genes, and of the overall genome; and phylogenetic congruence (i.e. whether genes from more than one trypanosomatid formed monophyletic assemblages). Genomic context and GC content graphs were drawn by GBrowse [87] and graphically edited for better use of space.

### Phylogenetic analyses

For phylogenetic analysis of each enzyme characterized in this work, corresponding putative orthologous genes from all domains of life were collected from the public databases by BLAST search (E-value cutoff of 1e-10, maximum of 10,000 matches accepted) against the full NCBI NR protein database, collecting sequences from as widespread taxonomic groups as possible and keeping one from each species (except for alignments with more than ~1,500 sequences, in which case one organism per genus was kept). Only sequences that were complete and aligned along at least 75% of the length of the query were selected. All analyses were performed at the protein sequence level. Sequences were aligned by Muscle v. 3.8.31 [89]. Phylogenetic inferences were performed by the maximum likelihood method, using RAxML v. 7.2.8 [90] and employing the WAG amino acid substitution model [91], with four gamma-distributed substitution rate heterogeneity categories and empirically determined residue frequencies (model PROTGAMMAWAGF). Each alignment was submitted to bootstrap analysis with 100 pseudo-replicates. Trees were initially drawn and formatted using TreeGraph2 [92] and Dendroscope [93], with subsequent cosmetic adjustments performed with the Inkscape vector image editor (http://inkscape.org). Phylogenetic conclusions have been displayed as strong in the summary table for phylogenetic results (Additional file 2) if BSV was 80 or greater, and moderate if BSV was between 50 and 80 – with one exception, EC:2.1.1.37, described in the results. Resulting phylogenetic trees are available from TreeBase (accession number S14564), as detailed in “Availability of Supporting Data” below.

## Availability of supporting data

The data sets supporting the results of this article are available in the GenBank and TreeBase repositories, under accession numbers AUXH00000000, AUXI00000000, AUXJ00000000, AUXK00000000, AUXL00000000, AUXM00000000, and AUXN00000000 (genome sequences of *S. culicis*, *C. acanthocephali*, *H. muscarum*, *S. oncopelti*, *A. desouzai*, *A. deanei*, and *S. galati*, respectively), and S14564, for the sequence alignments and phylogenetic trees (http://purl.org/phylo/treebase/phylows/study/TB2:S14564).

## Competing interests

The authors declare that they have no competing interests.

## Authors’ contributions

JMPA generated and analyzed metabolic pathway data, performed phylogenetic analyses, and wrote the manuscript. CCK generated and analyzed metabolic pathway data, and edited the manuscript. FMS and AGCM analyzed metabolic pathway data. MGS and GAB generated and analyzed genomic data. ATRV, MFS, and MMGT, participated in study design and data interpretation. MCM and EPC conceived the study and wrote the manuscript. All authors read and approved the manuscript.

## Supplementary Material

Additional file 1 **Genomic context and GC content for candidate HGT genes in the Trypanosomatidae analyzed in this work.** Arrows show TBLASTN alignments of the genome against UniRef100 and KEGG proteins. Alignment orientation is displayed in blue or red, except for the alignment for the gene currently in focus, which is colored black. Coordinates are in kilobases. Click here for file

Additional file 2 Summary of phylogenetic and genome coverage analyses of the candidate HGT genes in the Trypanosomatidae analyzed in this work and a few other genes of interest. Click here for file

Additional file 3 **Methionine salvage pathway.** Enzymes surrounded by a thick gray box were shown to be horizontally transferred from Bacteria (see main text). Metabolites – I: methionine; II: S-adenosylmethionine; III: S-adenosylmethioninamine; IV: S-methyl-5-thioadenosine; V: S-methyl-5-thioribose; VI: S-methyl-5-thioribose 1-phosphate; VII: S-methyl-5-thioribulose 1-phosphate; VIII: 2,3-diketomethylthiopentyl-1-phosphate; IX: 2-hydroxy-3-keto-5-methylthiopenctenyl-1-phosphate; X: 1,2-dihydroxy3-keto-5-methylthiopentene; XI: 4-methylthio-2-oxobutanoate. Enzymes – 2.5.1.6: methionine adenosyltransferase; 4.1.1.50: adenosylmethionine decarboxylase; 2.5.1.16: spermidine synthase; 3.2.2.9: adenosylhomocysteine nucleosidase; 3.2.2.16: methylthioadenosine nucleosidase; 2.7.1.100: S-methyl-5-thioribose kinase; 2.4.2.28: S-methyl-5’-thioadenosine phosphorylase; 5.3.1.23: S-methyl-5-thioribose-1-phosphate isomerase; 4.2.1.109: methylthioribulose 1-phosphate dehydratase; 3.1.3.77: acireductone synthase; 1.13.11.54: acireductone dioxygenase; 2.6.1.5: tyrosine transaminase; 2.6.1.57: aromatic-amino-acid transaminase. SHT: symbiont-harboring trypanosomatid; RT: regular trypanosomatid; TPE: trypanosomatid proteobacterial endosymbiont. Click here for file

Additional file 4 **Maximum likelihood phylogeny of diaminopimelate epimerase (EC:5.1.1.7).** Overall tree colored according to taxonomic affiliation of sequences. Values on nodes represent bootstrap support (only 50 or greater shown) and distance bar only applies to the overall tree and not to the detailed regions. Click here for file

Additional file 5 **Maximum likelihood phylogeny of diaminopimelate decarboxylase (EC:4.1.1.20).** Overall tree colored according to taxonomic affiliation of sequences. Values on nodes represent bootstrap support (only 50 or greater shown) and distance bar only applies to the overall tree and not to the detailed regions. Click here for file

Additional file 6 **Maximum likelihood phylogeny of serine O-acetyltransferase (EC:2.3.1.30).** Overall tree colored according to taxonomic affiliation of sequences. Values on nodes represent bootstrap support (only 50 or greater shown) and distance bar only applies to the overall tree and not to the detailed regions. Click here for file

Additional file 7 **Maximum likelihood phylogeny of cysteine synthase (EC:2.5.1.47).** Overall tree colored according to taxonomic affiliation of sequences. Values on nodes represent bootstrap support (only 50 or greater shown) and distance bar only applies to the overall tree and not to the detailed regions. Click here for file

Additional file 8 **Maximum likelihood phylogeny of homoserine O-succinyltransferase (EC:2.3.1.46).** Overall tree colored according to taxonomic affiliation of sequences. Values on nodes represent bootstrap support (only 50 or greater shown) and distance bar only applies to the overall tree and not to the detailed regions. Click here for file

Additional file 9 **Maximum likelihood phylogeny of DNA (cytosine-5-)-methyltransferase (EC:2.1.1.37).** Overall tree colored according to taxonomic affiliation of sequences. Values on nodes represent bootstrap support (only 50 or greater shown) and distance bar only applies to the overall tree and not to the detailed regions. Click here for file

Additional file 10 **Maximum likelihood phylogeny of cystathionine gamma-synthase, O-acetylhomoserine aminocarboxypropyltransferase, and cystathionine beta-lyase (EC:2.5.1.48, EC:2.5.1.49, and EC:4.4.1.8).** Overall tree colored according to taxonomic affiliation of sequences. Values on nodes represent bootstrap support (only 50 or greater shown) and distance bar only applies to the overall tree and not to the detailed regions. Click here for file

Additional file 11 **Maximum likelihood phylogeny of homocysteine S-methyltransferase (EC:2.1.1.10).** Overall tree colored according to taxonomic affiliation of sequences. Values on nodes represent bootstrap support (only 50 or greater shown) and distance bar only applies to the overall tree and not to the detailed regions. Click here for file

Additional file 12 **Maximum likelihood phylogeny of 5-methyltetrahydropteroyltriglutamate-homocysteine S-methyltransferase (EC:2.1.1.14).** Overall tree colored according to taxonomic affiliation of sequences. Values on nodes represent bootstrap support (only 50 or greater shown) and distance bar only applies to the overall tree and not to the detailed regions. Click here for file

Additional file 13 **Maximum likelihood phylogeny of S-methyl-5-thioribose kinase (EC:2.7.1.100).** Overall tree colored according to taxonomic affiliation of sequences. Values on nodes represent bootstrap support (only 50 or greater shown) and distance bar only applies to the overall tree and not to the detailed regions. Click here for file

Additional file 14 **Maximum likelihood phylogeny of L-threonine aldolase (EC:4.1.2.5).** Overall tree colored according to taxonomic affiliation of sequences. Values on nodes represent bootstrap support (only 50 or greater shown) and distance bar only applies to the overall tree and not to the detailed regions. Click here for file

Additional file 15 **Maximum likelihood phylogeny of (EC:4.2.1.20).** Overall tree colored according to taxonomic affiliation of sequences. Values on nodes represent bootstrap support (only 50 or greater shown) and distance bar only applies to the overall tree and not to the detailed regions. Click here for file

Additional file 16 **Maximum likelihood phylogeny of aminoacylase (EC:3.5.1.14).** Overall tree colored according to taxonomic affiliation of sequences. Values on nodes represent bootstrap support (only 50 or greater shown) and distance bar only applies to the overall tree and not to the detailed regions. Click here for file

Additional file 17 **Maximum likelihood phylogeny of acetylornithine deacetylase (EC:3.5.1.16).** Overall tree colored according to taxonomic affiliation of sequences. Values on nodes represent bootstrap support (only 50 or greater shown) and distance bar only applies to the overall tree and not to the detailed regions. Click here for file

Additional file 18 **Maximum likelihood phylogeny of argininosuccinate synthase (EC:6.3.4.5).** Overall tree colored according to taxonomic affiliation of sequences. Values on nodes represent bootstrap support (only 50 or greater shown) and distance bar only applies to the overall tree and not to the detailed regions. Click here for file

Additional file 19 **Maximum likelihood phylogeny of argininosuccinate lyase (EC:4.3.2.1).** Overall tree colored according to taxonomic affiliation of sequences. Values on nodes represent bootstrap support (only 50 or greater shown) and distance bar only applies to the overall tree and not to the detailed regions. Click here for file

Additional file 20 **Maximum likelihood phylogeny of arginase (EC:3.5.3.1).** Overall tree colored according to taxonomic affiliation of sequences. Values on nodes represent bootstrap support (only 50 or greater shown) and distance bar only applies to the overall tree and not to the detailed regions. Click here for file

Additional file 21 **Maximum likelihood phylogeny of ornithine cyclodeaminase (EC:4.3.1.12).** Overall tree colored according to taxonomic affiliation of sequences. Values on nodes represent bootstrap support (only 50 or greater shown) and distance bar only applies to the overall tree and not to the detailed regions. Click here for file

Additional file 22 Genbank accession numbers for Trypanosomatidae genes characterized in this study. Click here for file

Additional file 23 **GenBank locus tags for the *Ca.*Kinetoplastibacterium genes analyzed in this study.** Click here for file

## References

[B1] Baumann P Moran NA Baumann L The evolution and genetics of aphid endosymbionts BioScience 1997 47 12 20 10.2307/1313002

[B2] Wernegreen JJ Genome evolution in bacterial endosymbionts of insects Nat Rev Genet 2002 3 850 861 10.1038/nrg931 12415315

[B3] Wernegreen JJ Endosymbiosis: lessons in conflict resolution PLoS Biol 2004 2 E68 10.1371/journal.pbio.0020068 15024418 PMC368163

[B4] Moran NA Symbiosis Curr Biol 2006 16 R866 R871 10.1016/j.cub.2006.09.019 17055966

[B5] Moran NA McCutcheon JP Nakabachi A Genomics and evolution of heritable bacterial symbionts Annu Rev Genet 2008 42 165 190 10.1146/annurev.genet.41.110306.130119 18983256

[B6] Wernegreen JJ Strategies of genomic integration within insect-bacterial mutualisms Biol Bull 2012 223 112 122 22983037 10.1086/BBLv223n1p112 PMC3609409

[B7] McCutcheon JP von Dohlen CD An interdependent metabolic patchwork in the nested symbiosis of mealybugs Curr Biol 2011 21 1366 1372 10.1016/j.cub.2011.06.051 21835622 PMC3169327

[B8] Horn M Wagner M Bacterial endosymbionts of free-living amoebae J Eukaryot Microbiol 2004 51 509 514 10.1111/j.1550-7408.2004.tb00278.x 15537084

[B9] Heinz E Kolarov I Kästner C Toenshoff ER Wagner M Horn M **An*****Acanthamoeba*****sp. containing two phylogenetically different bacterial endosymbionts.** Environ Microbiol 2007 9 1604 1609 10.1111/j.1462-2920.2007.01268.x 17504498 PMC1974821

[B10] Nowack ECM Melkonian M Endosymbiotic associations within protists Philos Trans R Soc Lond B Biol Sci 2010 365 699 712 10.1098/rstb.2009.0188 20124339 PMC2817226

[B11] Chang KP Chang CS Sassa S Heme biosynthesis in bacterium-protozoon symbioses: enzymic defects in host hemoflagellates and complemental role of their intracellular symbiotes Proc Natl Acad Sci USA 1975 72 2979 2983 10.1073/pnas.72.8.2979 810795 PMC432902

[B12] Roitman I Camargo EP Endosymbionts of trypanosomatidae Parasitol Today (Regul. Ed.) 1985 1 143 144 10.1016/0169-4758(85)90060-2 15275586

[B13] Du Y Maslov DA Chang KP Monophyletic origin of beta-division proteobacterial endosymbionts and their coevolution with insect trypanosomatid protozoa *Blastocrithidia culicis* and *Crithidia* spp Proc Natl Acad Sci USA 1994 91 8437 8441 10.1073/pnas.91.18.8437 7521530 PMC44621

[B14] Motta MCM Catta-Preta CMC Schenkman S De Azevedo Martins AC Miranda K De Souza W Elias MC The bacterium endosymbiont of *Crithidia deanei* undergoes coordinated division with the host cell nucleus PLoS ONE 2010 5 e12415 10.1371/journal.pone.0012415 20865129 PMC2932560

[B15] Hoare CA Herpetosoma from man and other mammals The Trypanosomes of Mammals: A Zoological Monograph 1972 Oxford: Blackwell Scientific Publications 288 314

[B16] Wenyon CM Protozoology - A Manual for Medical Men, Veterinarians and Zoologists 1926 London: Bailliere, Tindall and Cox 1

[B17] Wallace FG The trypanosomatid parasites of insects and arachnids Exp Parasitol 1966 18 124 193 10.1016/0014-4894(66)90015-4 5325636

[B18] Vickerman K The evolutionary expansion of the trypanosomatid flagellates Int J Parasitol 1994 24 1317 1331 10.1016/0020-7519(94)90198-8 7729984

[B19] Newton BA A synthetic growth medium for the trypanosomid flagellate *Strigomonas (Herpetomonas) oncopelti* Nature 1956 177 279 280 13297025 10.1038/177279b0

[B20] Newton BS Nutritional requirements and biosynthetic capabilities of the parasitic flagellate *Strigomonas oncopelti* J Gen Microbiol 1957 17 708 717 10.1099/00221287-17-3-708 13491808

[B21] Kidder GW Davis JS Cousens K Citrulline utilization in *Crithidia* Biochem Biophys Res Commun 1966 24 365 369 10.1016/0006-291X(66)90165-3 5967097

[B22] Mundim MH Roitman I Hermans MA Kitajima EW Simple nutrition of *Crithidia deanei*, a reduviid trypanosomatid with an endosymbiont J Protozool 1974 21 518 521 10.1111/j.1550-7408.1974.tb03691.x 4278787

[B23] Menezes MCND Roitman I Nutritional requirements of *Blastocrithidia culicis*, a trypanosomatid with an endosymbiont J Eukaryotic Microbiol 1991 38 122 123

[B24] Teixeira MMG Borghesan TC Ferreira RC Santos MA Takata CSA Campaner M Nunes VLB Milder RV de Souza W Camargo EP Phylogenetic validation of the genera *Angomonas* and *Strigomonas* of trypanosomatids harboring bacterial endosymbionts with the description of New species of trypanosomatids and of proteobacterial symbionts Protist 2011 162 503 524 10.1016/j.protis.2011.01.001 21420905

[B25] Alves JMP Serrano MG Silva FM D Voegtly LJ Matveyev AV Teixeira MMG Camargo EP Buck GA Genome evolution and phylogenomic analysis of *candidatus* kinetoplastibacterium, the betaproteobacterial endosymbionts of *Strigomonas* and *Angomonas* Genome Biol Evol 2013 5 338 350 10.1093/gbe/evt012 23345457 PMC3590767

[B26] Motta MCM Martins AC De A De Souza SS Catta-Preta CMC Silva R Klein CC De Almeida LGP De Lima Cunha O Ciapina LP Brocchi M Colabardini AC De Araujo Lima B Machado CR De Almeida Soares CM Probst CM De Menezes CBA Thompson CE Bartholomeu DC Gradia DF Pavoni DP Grisard EC Fantinatti-Garboggini F Marchini FK Rodrigues-Luiz GF Wagner G Goldman GH Fietto JLR Elias MC Goldman MHS Sagot M-F Pereira M Stoco PH De Mendonça-Neto RP Teixeira SMR Maciel TEF De Oliveira Mendes TA Urményi TP De Souza W Schenkman S De Vasconcelos ATR Predicting the proteins of *Angomonas deanei*, *Strigomonas culicis* and their respective endosymbionts reveals New aspects of the trypanosomatidae family PLoS ONE 2013 8 e60209 10.1371/journal.pone.0060209 23560078 PMC3616161

[B27] Andersson SG Kurland CG Reductive evolution of resident genomes Trends Microbiol 1998 6 263 268 10.1016/S0966-842X(98)01312-2 9717214

[B28] Itoh T Martin W Nei M Acceleration of genomic evolution caused by enhanced mutation rate in endocellular symbionts Proc Natl Acad Sci USA 2002 99 12944 12948 10.1073/pnas.192449699 12235368 PMC130565

[B29] Gómez-Valero L Silva FJ Christophe Simon J Latorre A Genome reduction of the aphid endosymbiont *Buchnera aphidicola* in a recent evolutionary time scale Gene 2007 389 87 95 10.1016/j.gene.2006.10.001 17098378

[B30] Palmié-Peixoto IV Rocha MR Urbina JA de Souza W Einicker-Lamas M Motta MCM Effects of sterol biosynthesis inhibitors on endosymbiont-bearing trypanosomatids FEMS Microbiol Lett 2006 255 33 42 10.1111/j.1574-6968.2005.00056.x 16436059

[B31] De Azevedo-Martins AC Frossard ML de Souza W Einicker-Lamas M Motta MCM Phosphatidylcholine synthesis in *Crithidia deanei*: the influence of the endosymbiont FEMS Microbiol Lett 2007 275 229 236 10.1111/j.1574-6968.2007.00892.x 17714482

[B32] De Freitas-Junior PRG Catta-Preta CMC da Silva Andrade L Cavalcanti DP De Souza W Einicker-Lamas M Motta MCM Effects of miltefosine on the proliferation, ultrastructure, and phospholipid composition of *Angomonas deanei*, a trypanosomatid protozoan that harbors a symbiotic bacterium FEMS Microbiol Lett 2012 333 129 137 10.1111/j.1574-6968.2012.02607.x 22651853

[B33] Alfieri SC Camargo EP Trypanosomatidae: isoleucine requirement and threonine deaminase in species with and without endosymbionts Exp Parasitol 1982 53 371 380 10.1016/0014-4894(82)90080-7 6806116

[B34] Chang KP Trager W Nutritional significance of symbiotic bacteria in two species of hemoflagellates Science 1974 183 531 532 10.1126/science.183.4124.531 4203488

[B35] Fair DS Krassner SM Alanine aminotransferase and aspartate aminotransferase in *Leishmania tarentolae* J Protozool 1971 18 441 444 10.1111/j.1550-7408.1971.tb03352.x 5132319

[B36] Camargo EP Freymuller E Endosymbiont as supplier of ornithine carbamoyltransferase in a trypanosomatid Nature 1977 270 52 53 10.1038/270052a0 927516

[B37] Figueiredo EN Yoshida N Roitman C Camargo EP Enzymes of the ornithine-arginine metabolism of trypanosomatids of the genus *Crithidia* J Eukaryotic Microbiol 1978 25 546 549

[B38] Yoshida N Jankevicius JV Roitman I Camargo EP Enzymes of the ornithine-arginine metabolism of trypanosomatids of the genus ***Herpetomonas*** J Eukaryotic Microbiol 1978 25 550 555

[B39] Camargo EP Coelho JA Moraes G Figueiredo EN Trypanosoma spp., leishmania spp. and leptomonas spp.: enzymes of ornithine-arginine metabolism Exp Parasitol 1978 46 141 144 10.1016/0014-4894(78)90125-X 569593

[B40] Galinari S Camargo EP Trypanosomatid protozoa: survey of acetylornithinase and ornithine acetyltransferase Exp Parasitol 1978 46 277 282 10.1016/0014-4894(78)90141-8 569594

[B41] Galinari S Camargo EP Urea cycle enzymes in wild and aposymbiotic strains of *Blastocrithidia culicis* J Parasitology 1979 65 88 10.2307/3280208

[B42] Cowperthwaite J Weber MM Packer L Hutner SH Nutrition of *Herpetomonas (Strigomonas) culicidarum* Ann N Y Acad Sci 1953 56 972 981 10.1111/j.1749-6632.1953.tb30277.x 13139289

[B43] Kidder GW Dutta BN The growth and nutrition of *Crithidia fasciculata* J Gen Microbiol 1958 18 621 638 10.1099/00221287-18-3-621 13549694

[B44] Guttman HN First defined media for *Leptomonas* spp. from insects J Protozool 1966 13 390 392 10.1111/j.1550-7408.1966.tb01926.x 5969168

[B45] Guttman HN Patterns of methionine and lysine biosynthesis in the trypanosomatidae during growth Journal of Eukaryotic Microbiology 1967 14 267 271 10.1111/j.1550-7408.1967.tb01996.x 6038035

[B46] Gutteridge WE Presence and properties of diaminopimelic acid decarboxylases in the genus *Crithidia* Biochim Biophys Acta 1969 184 366 373 10.1016/0304-4165(69)90039-7 5809721

[B47] Krassner SM Flory B Essential amino acids in the culture of *Leishmania tarentolae* J Parasitol 1971 57 917 920 10.2307/3277829 5568347

[B48] Kidder GW Dewey VC Methionine or folate and phosphoenolpyruvate in the biosynthesis of threonine in *Crithidia fasciculata* J Protozool 1972 19 93 98 10.1111/j.1550-7408.1972.tb03420.x 5008854

[B49] Cross GAM Klein RA Baker JR *Trypanosoma cruzi*: growth, amino acid utilization and drug action in a defined medium Ann Trop Med Parasitology 1975 69 513 514

[B50] Anderson SJ Krassner SM Axenic culture of *Trypanosoma cruzi* in a chemically defined medium J Parasitol 1975 61 144 145 10.2307/3279125 1090715

[B51] Cross GA Klein RA Linstead DJ Utilization of amino acids by *Trypanosoma brucei* in culture: L-threonine as a precursor for acetate Parasitology 1975 71 311 326 10.1017/S0031182000046758 1187188

[B52] Mundim MH Roitman I Extra nutritional requirements of artificially aposymbiotic *Crithidia deanei* J Eukaryotic Microbiol 1977 24 329 331

[B53] Roitman I Mundim MH Azevedo HP Kitajima EW **Growth of*****Crithidia*****at high temperature:*****Crithidia hutneri*****sp. n. and*****Crithidia luciliae thermophila*****s. sp. n.** Journal of Eukaryotic Microbiology 1977 24 553 556

[B54] Yoshida N Camargo EP Ureotelism and ammonotelism in trypanosomatids J Bacteriol 1978 136 1184 1186 721777 10.1128/jb.136.3.1184-1186.1978 PMC218555

[B55] Hutner SH Bacchi CJ Baker H Lumsden WHR, Evans DA Nutrition of the kinetoplastida Biology of the Kinetoplastida. Vol. 2, Volume 2 1979 London & New York: Academic 645 691

[B56] Camargo EP Silva S Roitman I Souza W Jankevicius JV Dollet M Enzymes of ornithine-arginine metabolism in trypanosomatids of the genus *phytomonas* J Eukaryotic Microbiology 1987 34 439 441

[B57] Bono H Ogata H Goto S Kanehisa M Reconstruction of amino acid biosynthesis pathways from the complete genome sequence Genome Res 1998 8 203 210 10.1101/gr.8.3.203 9521924

[B58] Alves JMP Voegtly L Matveyev AV Lara AM da Silva FM Serrano MG Buck GA Teixeira MMG Camargo EP Identification and phylogenetic analysis of heme synthesis genes in trypanosomatids and their bacterial endosymbionts PLoS ONE 2011 6 e23518 10.1371/journal.pone.0023518 21853145 PMC3154472

[B59] Bhattacharjee JK alpha-Aminoadipate pathway for the biosynthesis of lysine in lower eukaryotes Crit Rev Microbiol 1985 12 131 151 10.3109/10408418509104427 3928261

[B60] Nishida H Distribution of genes for lysine biosynthesis through the aminoadipate pathway among prokaryotic genomes Bioinformatics 2001 17 189 191 10.1093/bioinformatics/17.2.189 11238076

[B61] Velasco AM Leguina JI Lazcano A Molecular evolution of the lysine biosynthetic pathways J Mol Evol 2002 55 445 459 10.1007/s00239-002-2340-2 12355264

[B62] Hudson AO Bless C Macedo P Chatterjee SP Singh BK Gilvarg C Leustek T Biosynthesis of lysine in plants: evidence for a variant of the known bacterial pathways Biochim Biophys Acta 2005 1721 27 36 10.1016/j.bbagen.2004.09.008 15652176

[B63] Torruella G Suga H Riutort M Peretó J Ruiz-Trillo I The evolutionary history of lysine biosynthesis pathways within eukaryotes J Mol Evol 2009 69 240 248 10.1007/s00239-009-9266-x 19669682

[B64] Hutner SH Provasoli L Comparative physiology: nutrition Annu Rev Physiol 1965 27 19 50 10.1146/annurev.ph.27.030165.000315 14268870

[B65] Nathan HA Cowperthwaite J Use of the trypanosomid flagellate, *Crithidia fasciculata*, for evaluating antimalarials Proc Soc Exp Biol Med 1954 85 117 119 10.3181/00379727-85-20803 13134307

[B66] Janakidevi K Dewey VC Kidder GW Serotonin in protozoa Arch Biochem Biophys 1966 113 758 759 10.1016/0003-9861(66)90259-1 5296346

[B67] Hutner SH Bacchi CJ Shapiro A Baker H Protozoa as tools for nutrition research Nutr Rev 1980 38 361 364 7454148 10.1111/j.1753-4887.1980.tb05942.x

[B68] Shigenobu S Watanabe H Hattori M Sakaki Y Ishikawa H Genome sequence of the endocellular bacterial symbiont of aphids *Buchnera* sp. APS Nature 2000 407 81 86 10.1038/35024074 10993077

[B69] Macdonald SJ Lin GG Russell CW Thomas GH Douglas AE The central role of the host cell in symbiotic nitrogen metabolism Proc Biol Sci 2012 279 2965 2973 10.1098/rspb.2012.0414 22513857 PMC3385485

[B70] Wilson ACC Ashton PD Calevro F Charles H Colella S Febvay G Jander G Kushlan PF Macdonald SJ Schwartz JF Thomas GH Douglas AE Genomic insight into the amino acid relations of the pea aphid, *Acyrthosiphon pisum*, with its symbiotic bacterium *Buchnera aphidicola* Insect Mol Biol 2010 19 Suppl 2 249 258 20482655 10.1111/j.1365-2583.2009.00942.x

[B71] Hansen AK Moran NA Aphid genome expression reveals host-symbiont cooperation in the production of amino acids Proc Natl Acad Sci USA 2011 108 2849 2854 10.1073/pnas.1013465108 21282658 PMC3041126

[B72] Sandström J Moran N How nutritionally imbalanced is phloem sap for aphids? Entomol Exp Appl 1999 91 203 210 10.1046/j.1570-7458.1999.00485.x

[B73] Dunn MF Niks D Ngo H Barends TRM Schlichting I Tryptophan synthase: the workings of a channeling nanomachine Trends Biochem Sci 2008 33 254 264 10.1016/j.tibs.2008.04.008 18486479

[B74] Meister A Biochemistry of the amino acids 1965 New York: Academic Press Inc.

[B75] Beutin L Eisen H Regulation of enzymes involved in ornithine/arginine metabolism in the parasitic trypanosomatid *Herpetomonas samuelpessoai* Mol Gen Genet 1983 190 278 283 10.1007/BF00330651 6576220

[B76] Frossard ML Seabra SH DaMatta RA de Souza W de Mello FG Machado Motta MC An endosymbiont positively modulates ornithine decarboxylase in host trypanosomatids Biochem Biophys Res Commun 2006 343 443 449 10.1016/j.bbrc.2006.02.168 16546131

[B77] Andersson JO Horizontal gene transfer between microbial eukaryotes Methods Mol Biol 2009 532 473 487 10.1007/978-1-60327-853-9_27 19271202

[B78] Mormann S Lömker A Rückert C Gaigalat L Tauch A Pühler A Kalinowski J Random mutagenesis in *Corynebacterium glutamicum* ATCC 13032 using an IS6100-based transposon vector identified the last unknown gene in the histidine biosynthesis pathway BMC Genomics 2006 7 205 10.1186/1471-2164-7-205 16901339 PMC1590026

[B79] Petersen LN Marineo S Mandalà S Davids F Sewell BT Ingle RA The missing link in plant histidine biosynthesis: *Arabidopsis* myoinositol monophosphatase-like2 encodes a functional histidinol-phosphate phosphatase Plant Physiol 2010 152 1186 1196 10.1104/pp.109.150805 20023146 PMC2832243

[B80] Korený L Lukes J Oborník M Evolution of the haem synthetic pathway in kinetoplastid flagellates: an essential pathway that is not essential after all? Int J Parasitol 2010 40 149 156 10.1016/j.ijpara.2009.11.007 19968994

[B81] Husnik F Nikoh N Koga R Ross L Duncan RP Fujie M Tanaka M Satoh N Bachtrog D Wilson ACC von Dohlen CD Fukatsu T McCutcheon JP Horizontal gene transfer from diverse bacteria to an insect genome enables a tripartite nested mealybug symbiosis Cell 2013 153 1567 1578 10.1016/j.cell.2013.05.040 23791183

[B82] Camargo EP Growth and differentiation in *Trypanosoma cruzi*. I. Origin of metacyclic trypanosomes in liquid media. *Rev. Inst. Med. Trop* Sao Paulo 1964 6 93 100 14177814

[B83] Ozaki LS Czeko YMT Morel CM Genomic DNA cloning and related techniques Genes and Antigens of Parasites. A Laboratory Manual 1984 Rio de Janeiro: Fundação Oswaldo Cruz 165 185

[B84] Alves JMP Buck GA Automated system for gene annotation and metabolic pathway reconstruction using general sequence databases Chem Biodivers 2007 4 2593 2602 10.1002/cbdv.200790212 18027373

[B85] Suzek BE Huang H McGarvey P Mazumder R Wu CH Uniref: comprehensive and non-redundant uniprot reference clusters Bioinformatics 2007 23 1282 1288 10.1093/bioinformatics/btm098 17379688

[B86] Ogata H Goto S Sato K Fujibuchi W Bono H Kanehisa M KEGG: Kyoto encyclopedia of genes and genomes Nucleic Acids Res 1999 27 29 34 10.1093/nar/27.1.29 9847135 PMC148090

[B87] Stein LD Mungall C Shu S Caudy M Mangone M Day A Nickerson E Stajich JE Harris TW Arva A Lewis S The generic genome browser: a building block for a model organism system database Genome Res 2002 12 1599 1610 10.1101/gr.403602 12368253 PMC187535

[B88] Marchler-Bauer A Lu S Anderson JB Chitsaz F Derbyshire MK DeWeese-Scott C Fong JH Geer LY Geer RC Gonzales NR Gwadz M Hurwitz DI Jackson JD Ke Z Lanczycki CJ Lu F Marchler GH Mullokandov M Omelchenko MV Robertson CL Song JS Thanki N Yamashita RA Zhang D Zhang N Zheng C Bryant SH CDD: a conserved domain database for the functional annotation of proteins Nucleic Acids Res 2011 39 D225 D229 10.1093/nar/gkq1189 21109532 PMC3013737

[B89] Edgar RC MUSCLE: a multiple sequence alignment method with reduced time and space complexity BMC Bioinformatics 2004 5 113 10.1186/1471-2105-5-113 15318951 PMC517706

[B90] Stamatakis A RAxML-VI-HPC: maximum likelihood-based phylogenetic analyses with thousands of taxa and mixed models Bioinformatics 2006 22 2688 2690 10.1093/bioinformatics/btl446 16928733

[B91] Whelan S Goldman N A general empirical model of protein evolution derived from multiple protein families using a maximum-likelihood approach Mol Biol Evol 2001 18 691 699 10.1093/oxfordjournals.molbev.a003851 11319253

[B92] Stöver BC Müller KF TreeGraph 2: combining and visualizing evidence from different phylogenetic analyses BMC Bioinformatics 2010 11 7 10.1186/1471-2105-11-7 20051126 PMC2806359

[B93] Huson DH Richter DC Rausch C Dezulian T Franz M Rupp R Dendroscope: an interactive viewer for large phylogenetic trees BMC Bioinformatics 2007 8 460 10.1186/1471-2105-8-460 18034891 PMC2216043

