# Supplementary material for: Endosymbiosis in trypanosomatids: the genomic cooperation between bacterium and host in the synthesis of essential amino acids is heavily influenced by multiple horizontal gene transfers
Source: BMC Evol Biol. 2013 Sep 9;13:190. doi: 10.1186/1471-2148-13-190 (PMC3846528; doi:10.1186/1471-2148-13-190)

1.1.1.3 – Homoserine dehydrogenase, *Angomonas desouzai*. Contig's GC content: 46.15%.

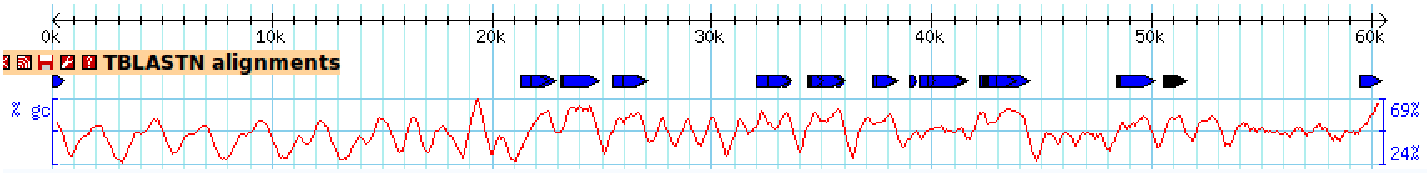

5.1.1.7 – Diaminopimelate epimerase, *Herpetomonas muscarum*. Contig's GC content: 54.84%.

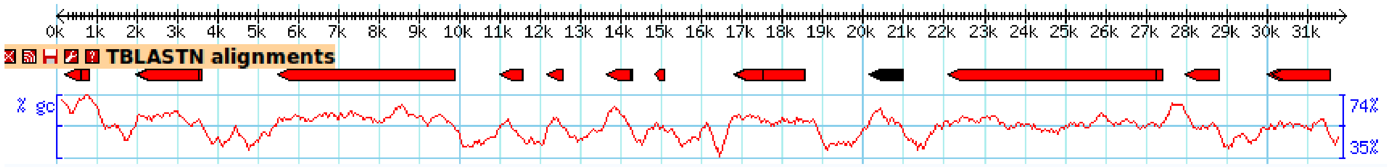

4.1.1.20 – Diaminopimelate decarboxylase, *Strigomonas galati*. Contig's GC content: 53.05%.

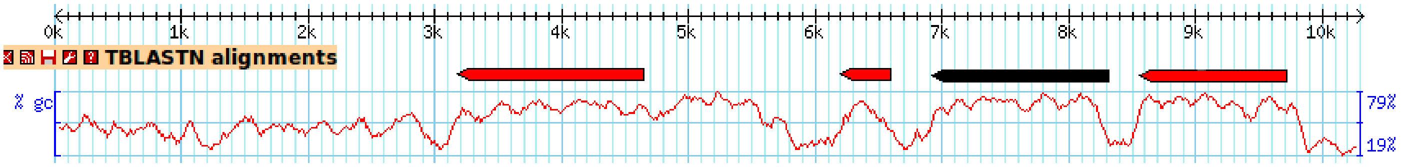

2.3.1.30 – Serine O-acetyltransferase, *Angomonas deanei*. Contig's GC content: 53.20%.

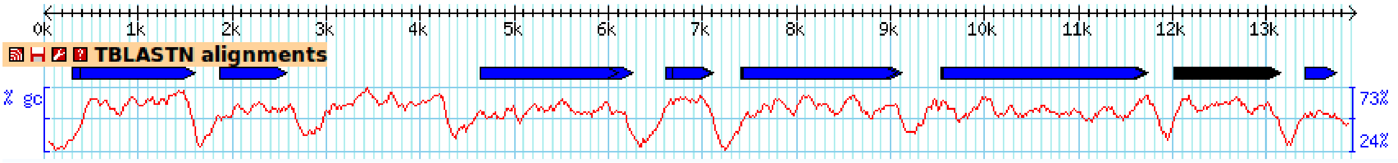

2.5.1.47 – Cysteine synthase, *Strigomonas galati*. Contig's GC content: 55.93%.

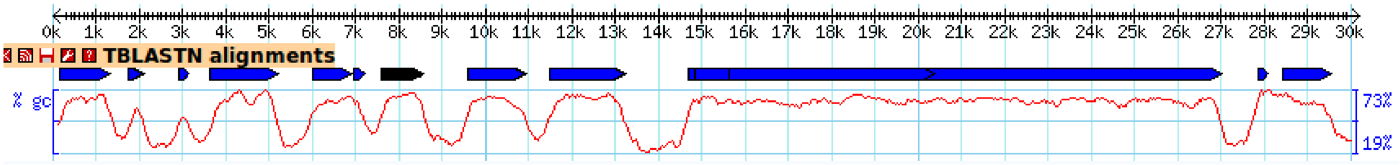

2.3.1.46 – Homoserine O-succinyltransferase, *Strigomonas galati*. Contig's GC content: 46.91%.

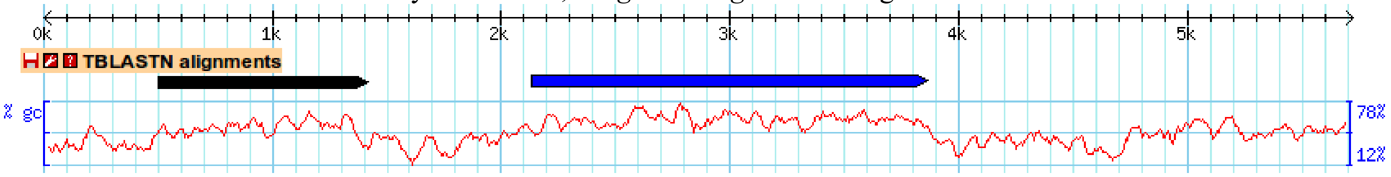

2.1.1.37 – DNA (cytosine-5-)-methyltransferase, *Strigomonas galati*. Contig's GC content: 59.10%.

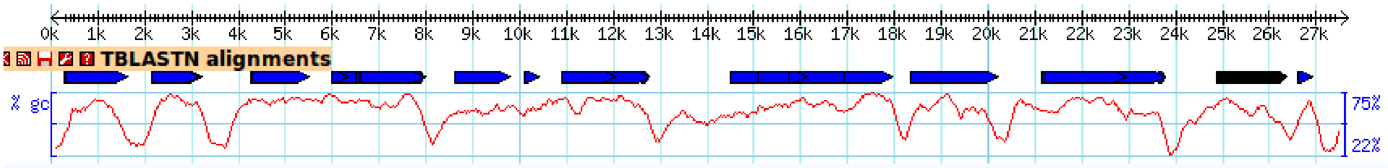

2.5.1.49 – O-acetylhomoserine aminocarboxypropyltransferase, *Angomonas deanei*. Contig's GC content: 54.02%.

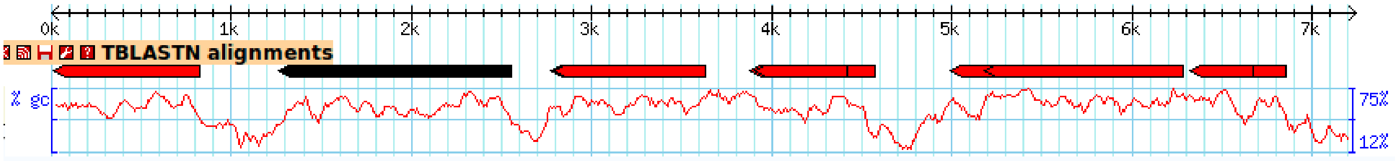

4.4.1.8 – Cystathionine beta-lyase, *Herpetomonas muscarum*. Contig's GC content: 61.10%.

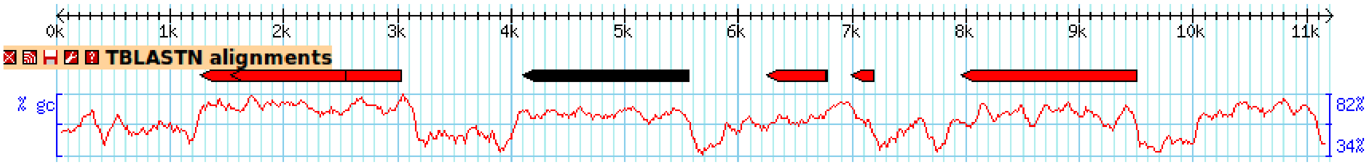

2.1.1.10 – Homocysteine S-methyltransferase, *Crithidia acanthocephali*. Contig's GC content: 62.28%.

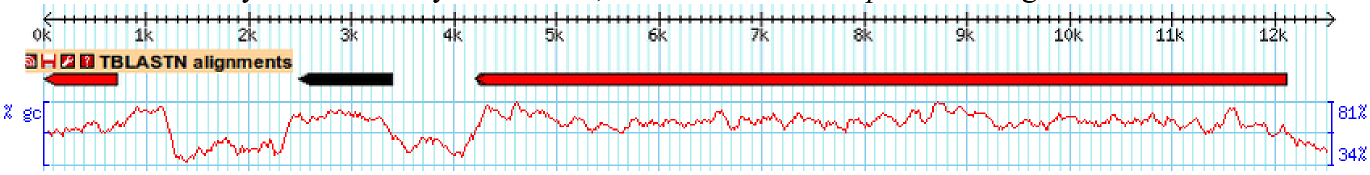

2.1.1.14 – 5-methyltetrahydropteroyltriglutamate-homocysteine S-methyltransferase (left) and **4.2.1.20** – tryptophan synthase (right), *Strigomonas culicis*. Contig's GC content: 48.69%.

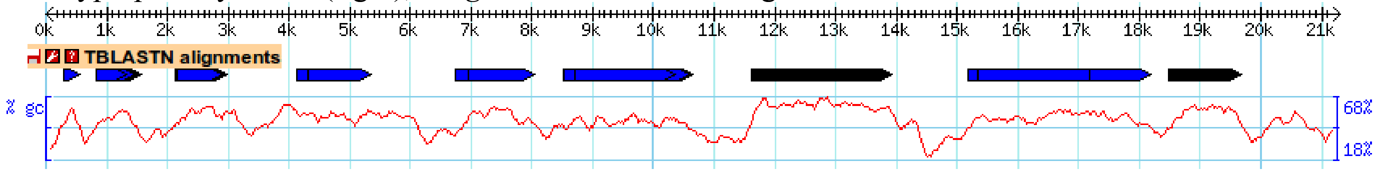

4.1.2.5 – Threonine aldolase, *Strigomonas culicis*. Contig's GC content: 57.13%.

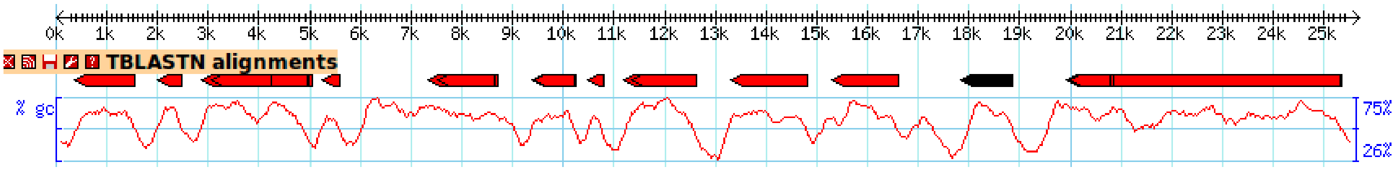

3.5.1.14 – Aminoacylase, *Angomonas deanei*. Contig's GC content: 51.80%.

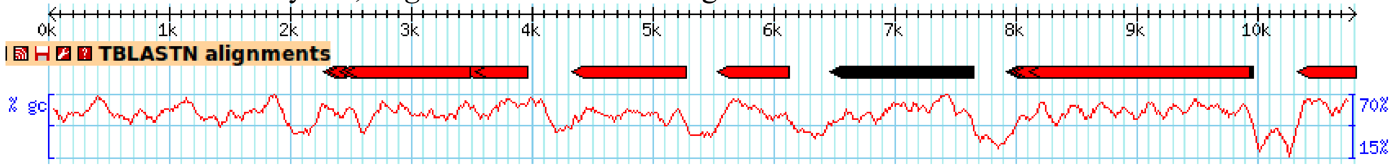

3.5.1.16 – Acetylornithine deacetylase, *Angomonas desouzai*. Contig's GC content: 47.01%.

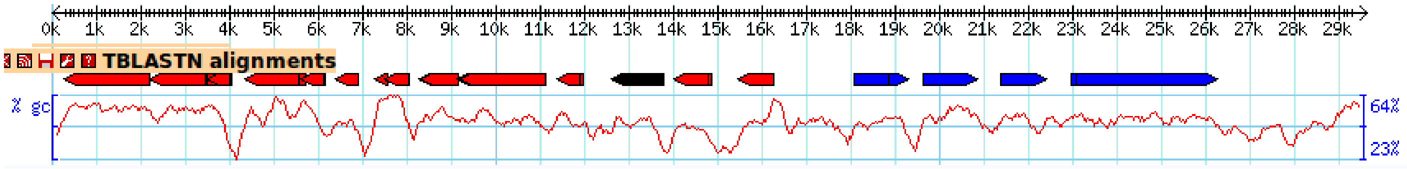

6.3.4.5 – Argininosuccinate synthase (left) and **4.3.2.1** – Argininosuccinate lyase (right), *Angomonas deanei*. Contig's GC content: 51.45%.

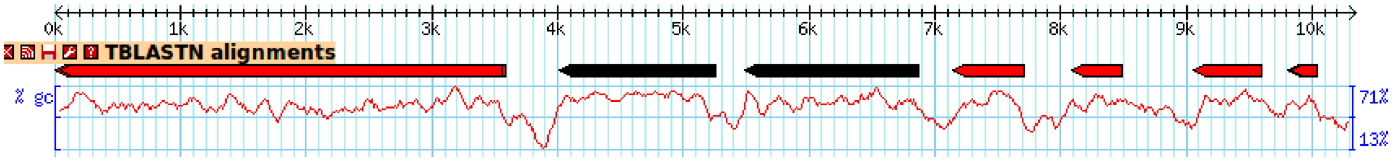

3.5.3.1 – Arginase, *Herpetomonas muscarum*. Contig's GC content: 54.07%.

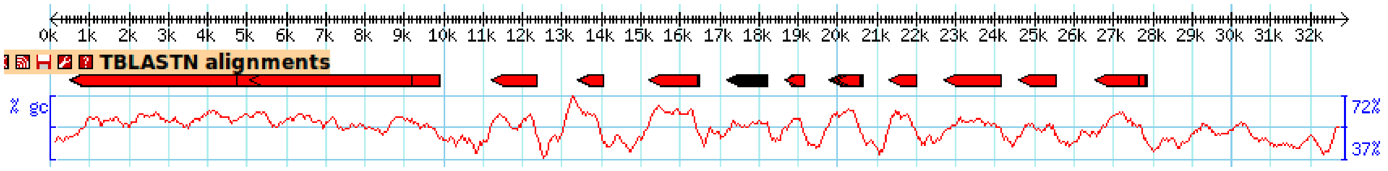

4.3.1.12 – Ornithine cyclodeaminase, *Angomonas deanei*. Contig's GC content: 48.93%.

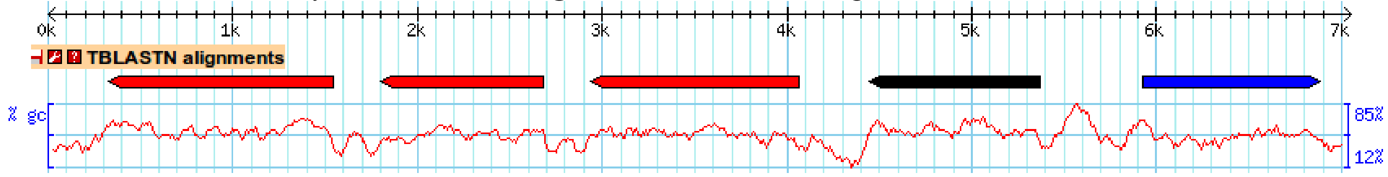

Supplement: Additional file 1 — Genomic context and GC content for candidate HGT genes in the Trypanosomatidae analyzed in this work. Arrows show TBLASTN alignments of the genome against UniRef100 and KEGG proteins. Alignment orientation is displayed in blue or red, except for the alignment for the gene currently in focus, which is colored black. Coordinates are in kilobases. [file 1471-2148-13-190-S1.pdf]
