# Supplementary material for: Endosymbiosis in trypanosomatids: the genomic cooperation between bacterium and host in the synthesis of essential amino acids is heavily influenced by multiple horizontal gene transfers
Source: BMC Evol Biol. 2013 Sep 9;13:190. doi: 10.1186/1471-2148-13-190 (PMC3846528; doi:10.1186/1471-2148-13-190)
Supplement: Additional file 2 — Summary of phylogenetic and genome coverage analyses of the candidate HGT genes in the Trypanosomatidae analyzed in this work and a few other genes of interest. [file 1471-2148-13-190-S2.pdf]

Summary of the phylogenetic and sequencing coverage analyses

EC number	Enzyme name	Pathway	Alignment length	# distinct aligment patterns	Presence	Cluster / sister group of trypanosomatids	Cluster / sister group of TPEs	Figure / Additional file	Genome's average coverage*	Contig's average coverage*	Gene's average coverage*	Genome*
5.1.1.7	diaminopimelate epimerase	Lysine	1661	1409	*Herpetomonas* and TPEs	Group within Bacteroidetes (BSV=99).	Alcaligenaceae family (BS=98).	Additional file 2	18x	17x	21x	*H. muscarum*
4.1.1.20	diaminopimelate decarboxylase	Lysine	2171	1869	SHTs (*A. desouzai* not in the tree, incomplete sequence similar to *A. deanei*), *Herpetomonas* and TPEs.	Group with a few other Eukaryota (Dictyostelium, Polysphondylium, and Capsaspora, BSV=65), all as the sister group of a large group of Actinobacteria (BSV=79).	Alcaligenaceae family (BS=95).	Additional file 3	28x	19x	21x	*S. galati*
2.3.1.30	serine O-acetyltransferase	Cysteine	1500	1248	SHTs and RTs	Group inside a group of diverse bacterial taxa (BS=80), including Bacteroidetes, Betaproteobacteria, Gammaproteobacteria and Firmicutes.	-	Additional file 4	23x	19x	31x	*A. deanei*
2.5.1.47	cysteine synthase	Cysteine	2809	2577	SHTs and RTs	SHTs, RTs (one copy of *T. cruzi* CL Brener) and one *Entamoeba* sp. clade has Actinobacteria as sister group (low BS), and far from the other eukaryotic groups.	-	Additional file 5	28x	23x	28x	*S. galati*
2.3.1.46	homoserine O-succinyltransferase	Cysteine – Methionine	975	808	SHTs and *Herpetomonas*	Trypanosomatid clade (BS=91). Group within the Bacteroidetes (BS=89).	-	Additional file 6	28x	19x	16x	*S. galati*
2.1.1.37	DNA (cytosine-5-)-methyltransferase	Cysteine – Methionine	2123	2095	SHTs and RTs	The few Eukaryota in the tree form a clade (low BS) among the several different phyla of Bacteria (low BS).	-	Additional file 7	28x	23x	22x	*S. galati*
2.5.1.48	cystathionine gamma-synthase	Cysteine – Methionine	2848	2522	SHTs and RTs	Group with Trypanosoma sp. and a few other Eukaryota, mostly Apicomplexa and Stramenopiles, within Acidobacteria (BS=94).	-	Additional file 8	ND	ND	ND	
2.5.1.49	O-acetylhomoserine aminocarboxypropyltransferase	Cysteine – Methionine	2848	2522	SHTs and *Herpetomonas*	Cluster with diverse groups of Bacteria (low BS).	-	Additional file 8	24x	27x	53x	*A. desouzai*
4.4.1.8	cystathionine beta-lyase	Cysteine – Methionine	2848	2522	SHTs and RTs	Two copies: one clusters with eukaryotes (BS=95) and the other seems to be of bacterial descent, grouping mostly with Alphaproteobacteria of the Rhizobiales order (BS=99).	-	Additional file 8	18x	20x	21x	*H. muscarum*
2.1.1.10	homocysteine S-methyltransferase	Methionine	2771	2445	SHTs and RTs	Clade SHTs and RTs (BS=96) grouped next to mostly Gammaproteobacteria (low BS).	-	Additional file 9	18x	14x	11x	*C. acanthocephali*
2.1.1.14	5-methyltetrahydropteroyltriglutamate---homocysteine S-methyltransferase	Methionine	1811	1662	SHTs and RTs (except *Herpetomonas*)	The *Angomonas* species group with *C. acanthocephali* (BS=98) while the *Strigomonas* group with *Leishmania* (BS=100). The trypanosomatids group deep within the Gammaproteobacteria (BS=74).	-	Additional file 10	23x	15x	15x	*S. culicis*
2.7.1.100	S-methyl-5-thioribose kinase	Methionine salvage	1358	1080	*C. acanthocephali* and *Herpetomonas*	Group deep within the Gammaproteobacteria (BS=97).	-	Additional file 11	18x	15x	24x	*H. muscarum*
1.1.1.3	homoserine dehydrogenase	Cysteine – Methionine Threonine	2189	1921	SHTs, RTs and TPEs	Cluster within the Firmicutes, with *Solibacillus silverstris* and *Lysinibacillus fusiformis* and *L. sphaericus* as sister group (BS=100).	Placed in the Alcaligenaceae family (BS=91).	Figure 8	24x	26x	34x	*A. desouzai*
4.1.2.5	L-threonine aldolase	Glycine <=> Threonine	2083	1700	SHTs and RTs (except *Herpetomonas*)	SHTs and RTs group in very distant clades: *Leishmania* and *C. acanthocephali* group within Firmicutes, specially *Clostridium* (BS=63), while the SHT group basally with Eukaryota, interrupted by an assorted group of Bacteria (low BS). SHTs and RTs are in opposite sides of the tree, separated by a realtively long branch.	-	Additional file 12	23x	16x	12x	*S. culicis*
4.2.1.20	tryptophan synthase	Tryptophan	1392	1339	SHTs, *Herpetomonas* and TPEs	SHTs and *Herpetomonas* group together (BS=50) and group robustly with the Bacteroidetes phylum (BS=97).	Group with the Alcaligenaceae family (BS=90).	Additional file 13	23x	15x	21x	*S. culicis*
3.5.1.14	aminoacylase	Ornithine	2397	1920	SHTs and RTs	*Angomonas* has two different copies (in different clades), while *Strigomonas* have one (grouped with *Angomonas*, BS=96). RTs also have multiple different copies. All gene copies group as one clade and have as neasrest siter group a Gammaproteobacterium (BS=98). Group with Bacteria of different phyla (low BS).	-	Additional file 14	23x	36x	36x	*A. deanei*
3.5.1.16	acetylornithine deacetylase	Ornithine	920	831	SHTs and RTs	The gene copies of SHTs and RTs group together (BS=84). Group within mainly Betaproteobacteria (BS= 80), and no other Eukaryota seem to have these orthologs.	-	Additional file 15	24x	17x	13x	*A. desouzai*
6.3.4.5	argininosuccinate synthase	Arginine	2322	1311	SHTs and RTs	Clade trypanosomatid (BS=100) group within mainly Firmicutes (BS=69).	-	Additional file 16	23x	28x	35x	*A. deanei*
4.3.2.1	argininosuccinate lyase	Arginine	1960	1520	SHTs and RTs	Trypanosomatid clade groups within Firmicutes (BS=82).	-	Additional file 17	23x	28x	31x	*A. deanei*
3.5.3.1	arginase	Ornithine	2403	1548	SHTs and RTs	All but the *Herpetomonas* ortholog are of eukaryotic origin (BS=61). *Herpetomonas* gene groups in a distant bacterial clade (BS=79) containing several different assorted phyla.	-	Additional file 18	18x	21x	15x	*H. muscarum*
4.3.1.12	ornithine cyclodeaminase	Ornithine <=> Proline	1108	990	Only in SHTs	Group close to several Alcaligenaceae (BS=63).	-	Additional file 19	23x	15x	19x	*A. deanei*

Cells in red and pale yellow indicate HGT cases with strong and moderate support (bootstrap and tree patterns), respectively, while gray cells indicate cases that are currently indeterminate and can not be assigned to an HGT event.
* Genome, contig, and gene average sequencing coverages were calculated for the organism indicated in the "Genome" column. ND: not determined.
Abbreviations: TPE = trypanosomatid proteobacterial endosymbiont; SHT = symbiont-harboring trypanosomatid; RT = regular trypanosomatid.
