# Supplementary figures and images for: Endosymbiosis in trypanosomatids: the genomic cooperation between bacterium and host in the synthesis of essential amino acids is heavily influenced by multiple horizontal gene transfers
Source: BMC Evol Biol. 2013 Sep 9;13:190. doi: 10.1186/1471-2148-13-190 (PMC3846528; doi:10.1186/1471-2148-13-190)

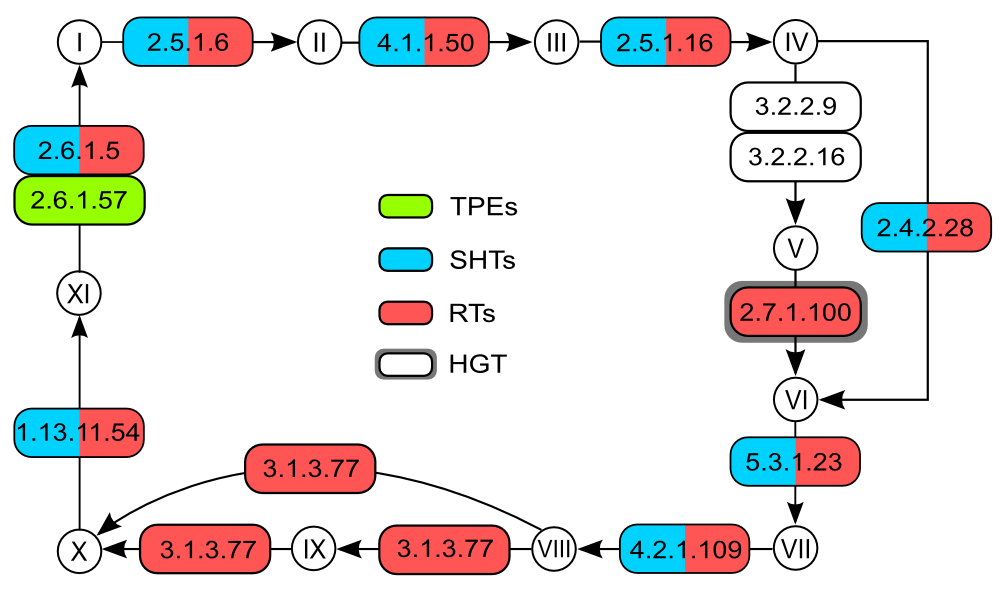

Supplement: Additional file 3 — Methionine salvage pathway. Enzymes surrounded by a thick gray box were shown to be horizontally transferred from Bacteria (see main text). Metabolites – I: methionine; II: S-adenosylmethionine; III: S-adenosylmethioninamine; IV: S-methyl-5-thioadenosine; V: S-methyl-5-thioribose; VI: S-methyl-5-thioribose 1-phosphate; VII: S-methyl-5-thioribulose 1-phosphate; VIII: 2,3-diketomethylthiopentyl-1-phosphate; IX: 2-hydroxy-3-keto-5-methylthiopenctenyl-1-phosphate; X: 1,2-dihydroxy3-keto-5-methylthiopentene; XI: 4-methylthio-2-oxobutanoate. Enzymes – 2.5.1.6: methionine adenosyltransferase; 4.1.1.50: adenosylmethionine decarboxylase; 2.5.1.16: spermidine synthase; 3.2.2.9: adenosylhomocysteine nucleosidase; 3.2.2.16: methylthioadenosine nucleosidase; 2.7.1.100: S-methyl-5-thioribose kinase; 2.4.2.28: S-methyl-5’-thioadenosine phosphorylase; 5.3.1.23: S-methyl-5-thioribose-1-phosphate isomerase; 4.2.1.109: methylthioribulose 1-phosphate dehydratase; 3.1.3.77: acireductone synthase; 1.13.11.54: acireductone dioxygenase; 2.6.1.5: tyrosine transaminase; 2.6.1.57: aromatic-amino-acid transaminase. SHT: symbiont-harboring trypanosomatid; RT: regular trypanosomatid; TPE: trypanosomatid proteobacterial endosymbiont. [file 1471-2148-13-190-S3.png]

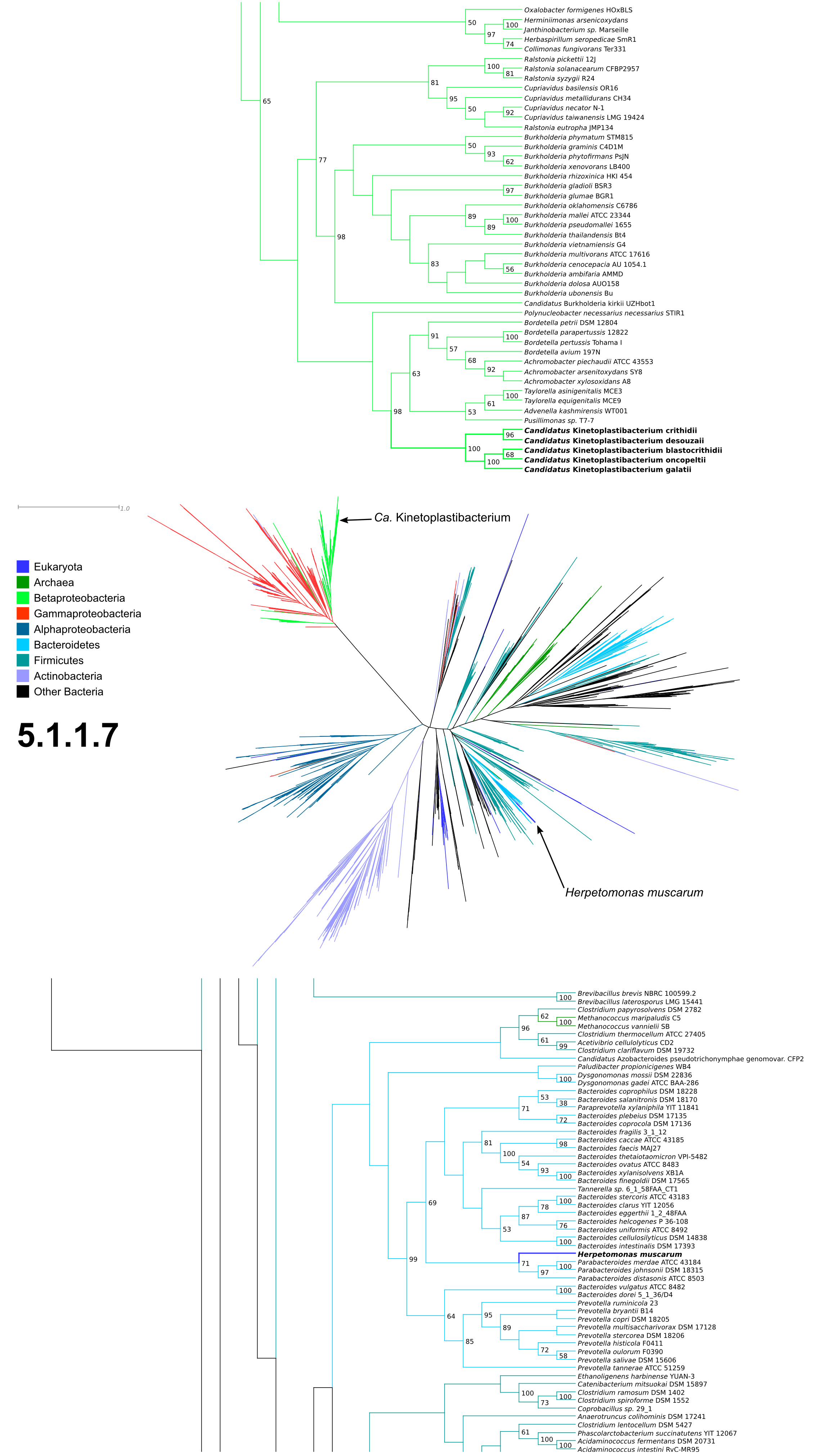

Supplement: Additional file 4 — Maximum likelihood phylogeny of diaminopimelate epimerase (EC:5.1.1.7). Overall tree colored according to taxonomic affiliation of sequences. Values on nodes represent bootstrap support (only 50 or greater shown) and distance bar only applies to the overall tree and not to the detailed regions. [file 1471-2148-13-190-S4.png]

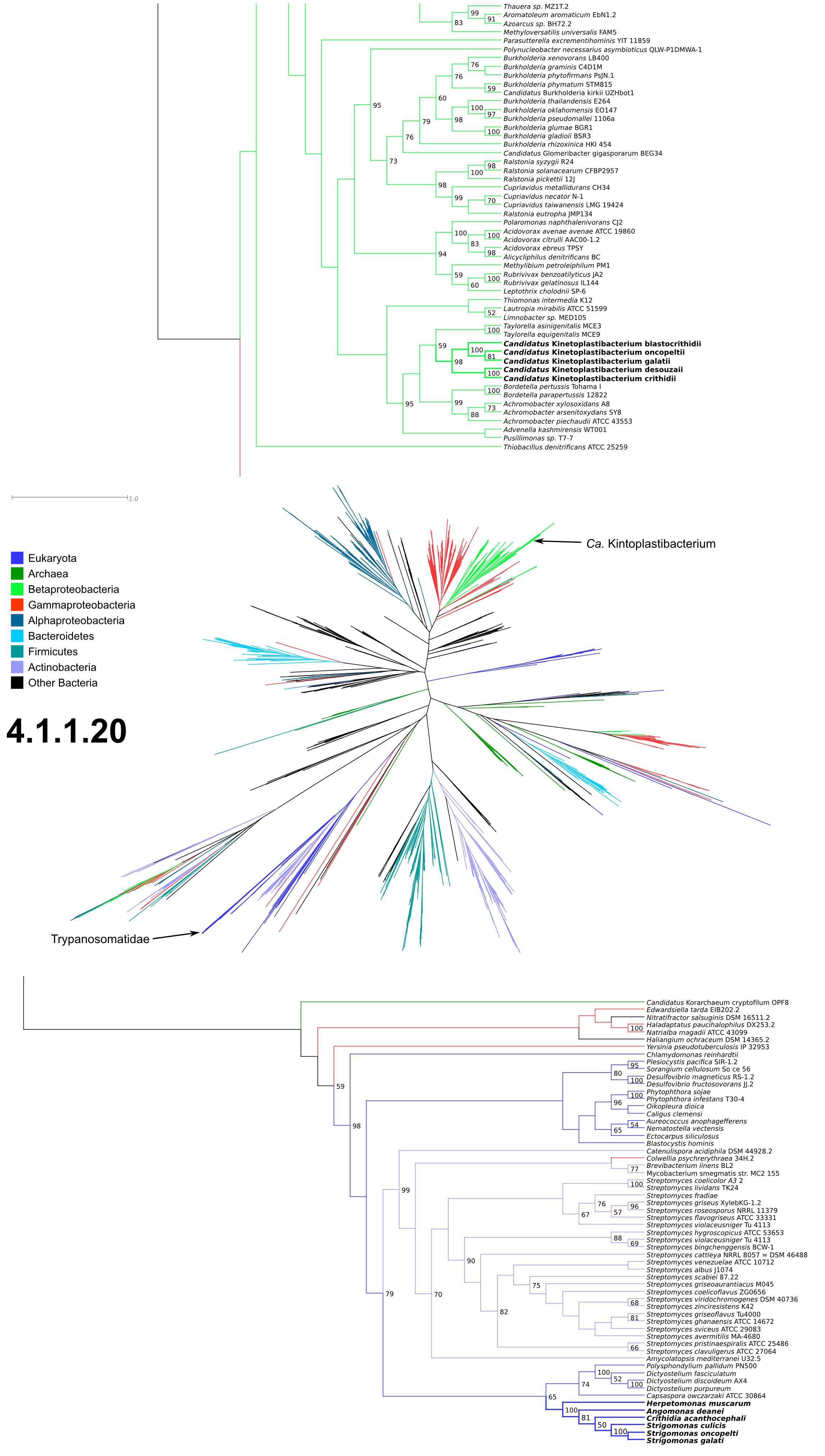

Supplement: Additional file 5 — Maximum likelihood phylogeny of diaminopimelate decarboxylase (EC:4.1.1.20). Overall tree colored according to taxonomic affiliation of sequences. Values on nodes represent bootstrap support (only 50 or greater shown) and distance bar only applies to the overall tree and not to the detailed regions. [file 1471-2148-13-190-S5.png]

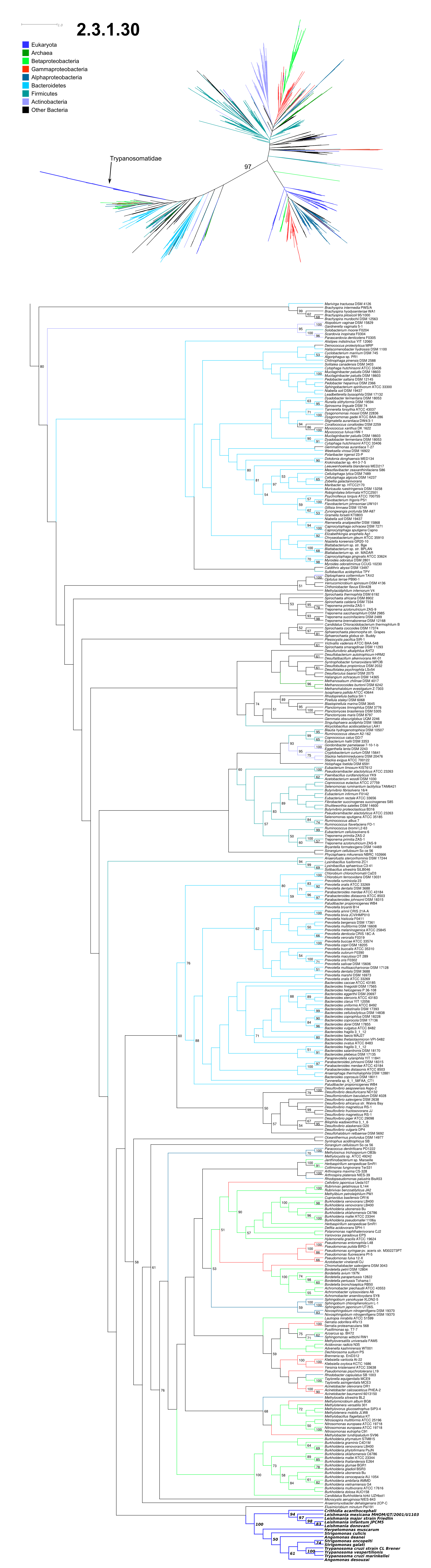

Supplement: Additional file 6 — Maximum likelihood phylogeny of serine O-acetyltransferase (EC:2.3.1.30). Overall tree colored according to taxonomic affiliation of sequences. Values on nodes represent bootstrap support (only 50 or greater shown) and distance bar only applies to the overall tree and not to the detailed regions. [file 1471-2148-13-190-S6.png]

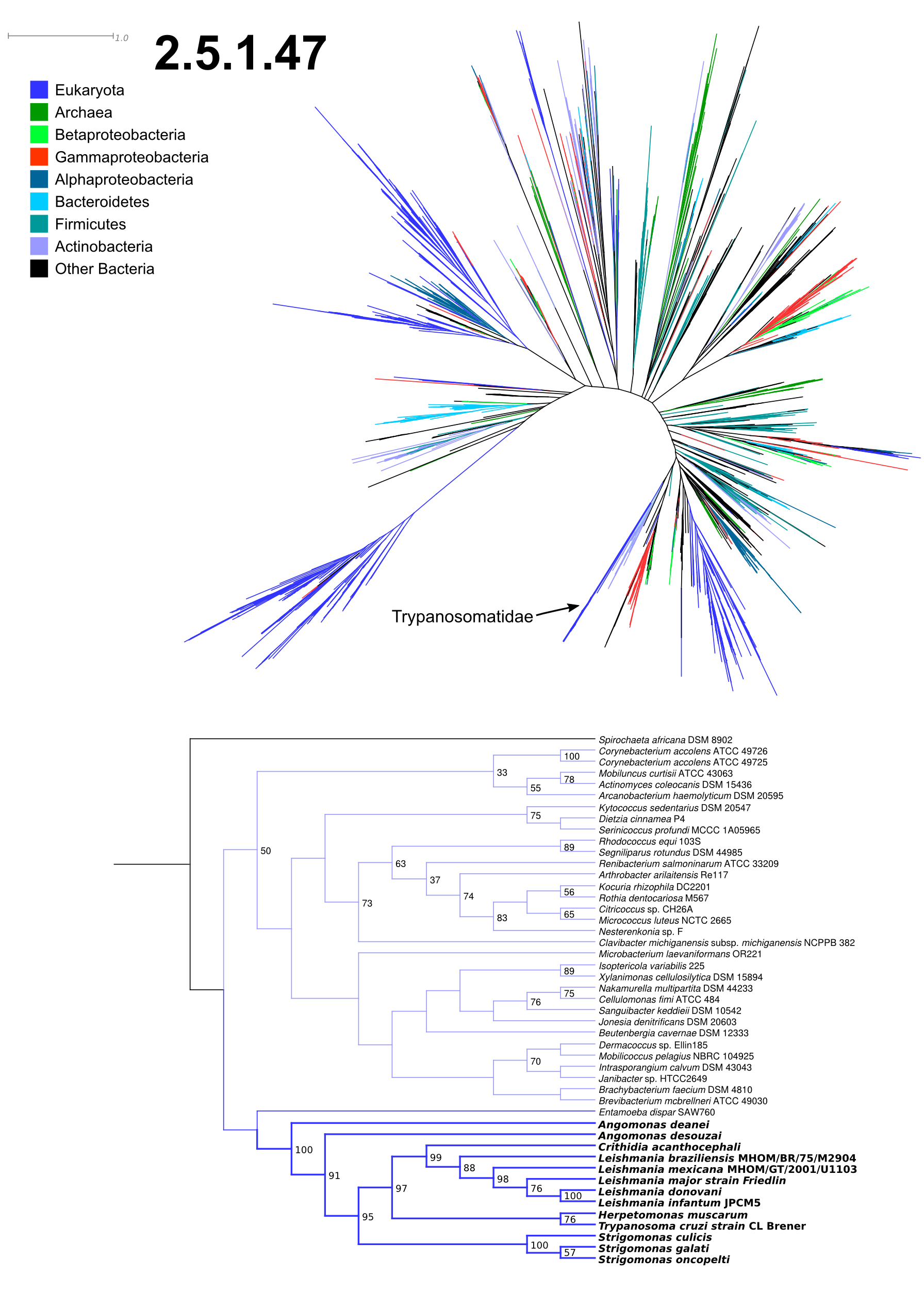

Supplement: Additional file 7 — Maximum likelihood phylogeny of cysteine synthase (EC:2.5.1.47). Overall tree colored according to taxonomic affiliation of sequences. Values on nodes represent bootstrap support (only 50 or greater shown) and distance bar only applies to the overall tree and not to the detailed regions. [file 1471-2148-13-190-S7.png]

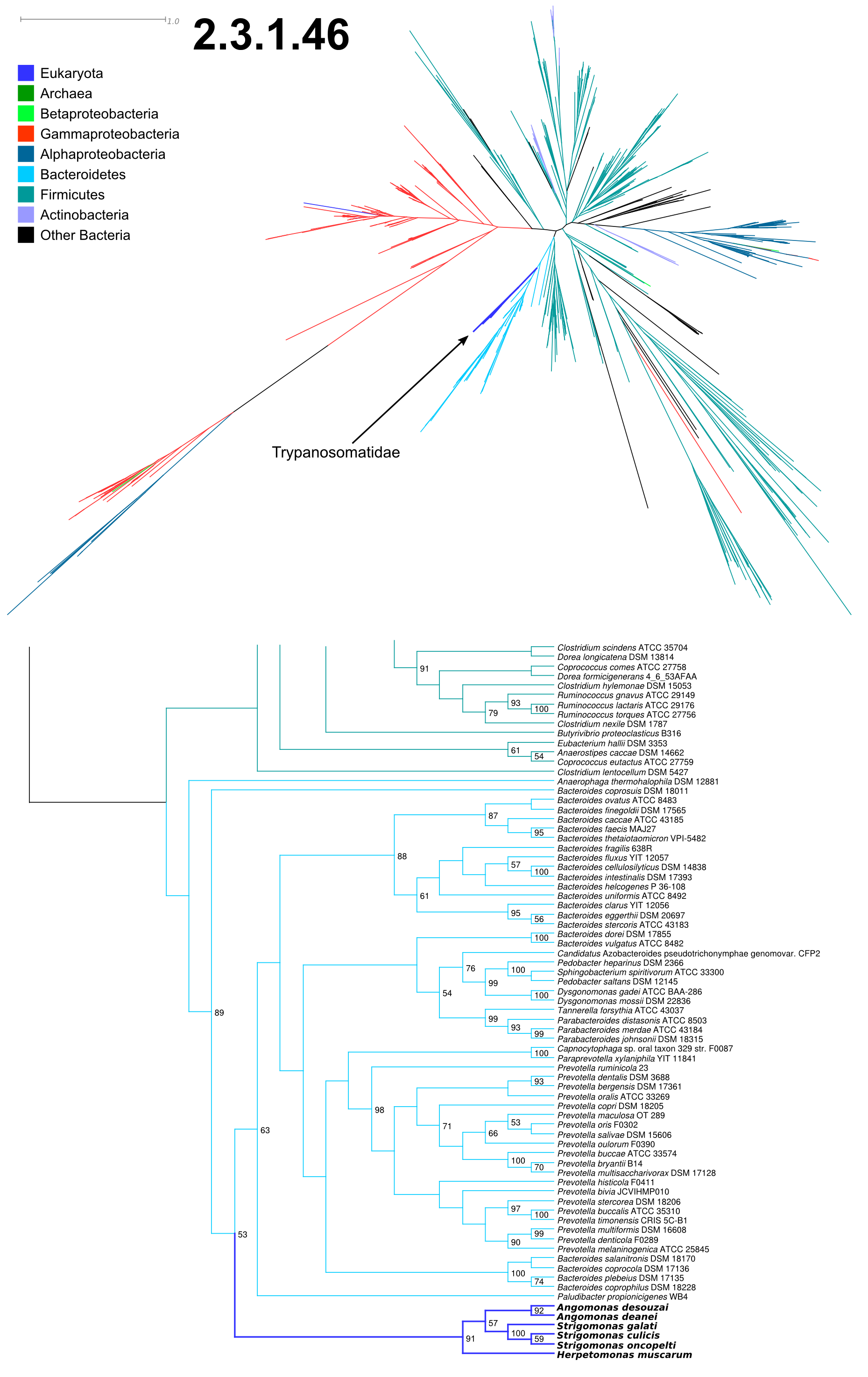

Supplement: Additional file 8 — Maximum likelihood phylogeny of homoserine O-succinyltransferase (EC:2.3.1.46). Overall tree colored according to taxonomic affiliation of sequences. Values on nodes represent bootstrap support (only 50 or greater shown) and distance bar only applies to the overall tree and not to the detailed regions. [file 1471-2148-13-190-S8.png]

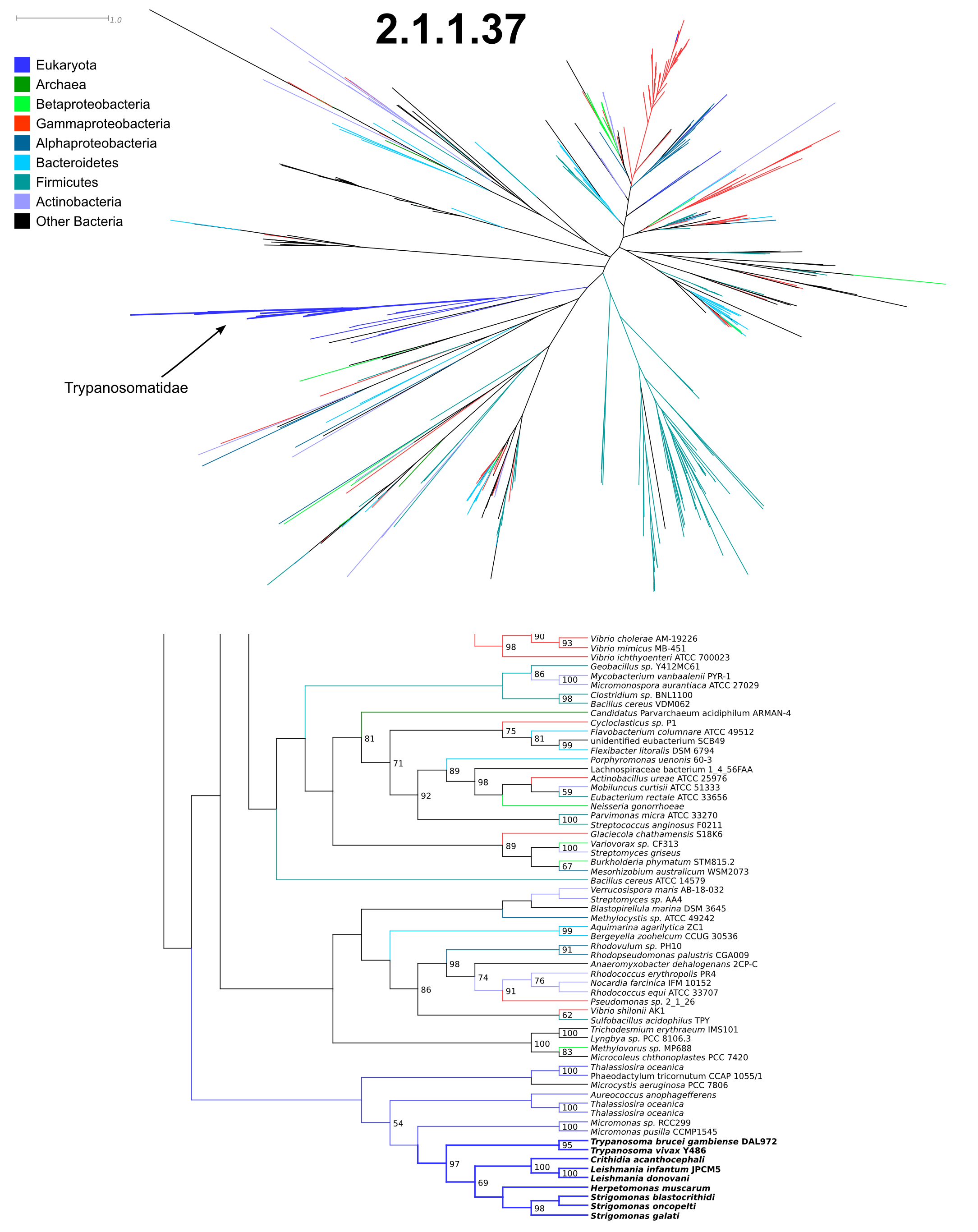

Supplement: Additional file 9 — Maximum likelihood phylogeny of DNA (cytosine-5-)-methyltransferase (EC:2.1.1.37). Overall tree colored according to taxonomic affiliation of sequences. Values on nodes represent bootstrap support (only 50 or greater shown) and distance bar only applies to the overall tree and not to the detailed regions. [file 1471-2148-13-190-S9.png]

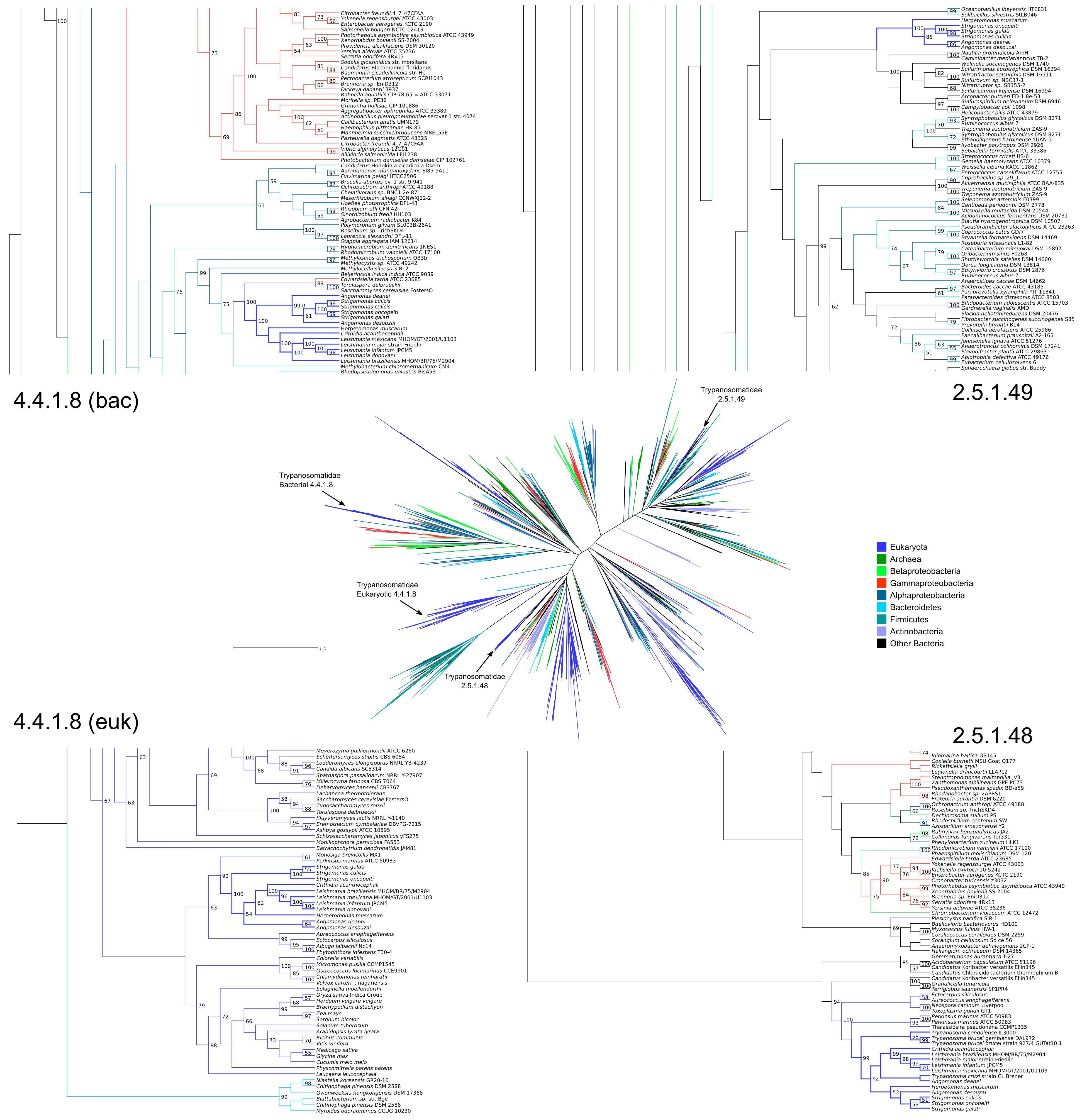

Supplement: Additional file 10 — Maximum likelihood phylogeny of cystathionine gamma-synthase, O-acetylhomoserine aminocarboxypropyltransferase, and cystathionine beta-lyase (EC:2.5.1.48, EC:2.5.1.49, and EC:4.4.1.8). Overall tree colored according to taxonomic affiliation of sequences. Values on nodes represent bootstrap support (only 50 or greater shown) and distance bar only applies to the overall tree and not to the detailed regions. [file 1471-2148-13-190-S10.png]

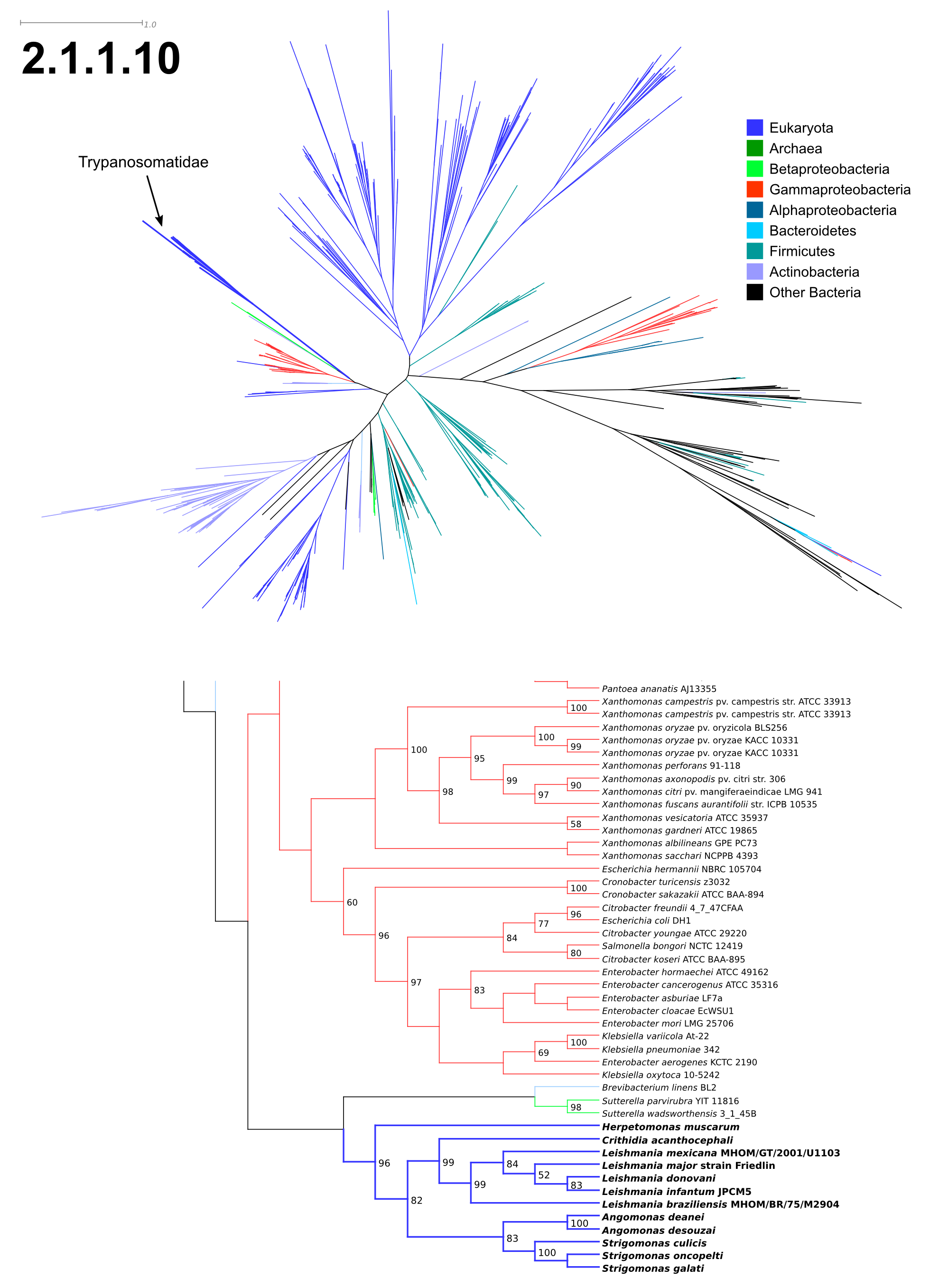

Supplement: Additional file 11 — Maximum likelihood phylogeny of homocysteine S-methyltransferase (EC:2.1.1.10). Overall tree colored according to taxonomic affiliation of sequences. Values on nodes represent bootstrap support (only 50 or greater shown) and distance bar only applies to the overall tree and not to the detailed regions. [file 1471-2148-13-190-S11.png]

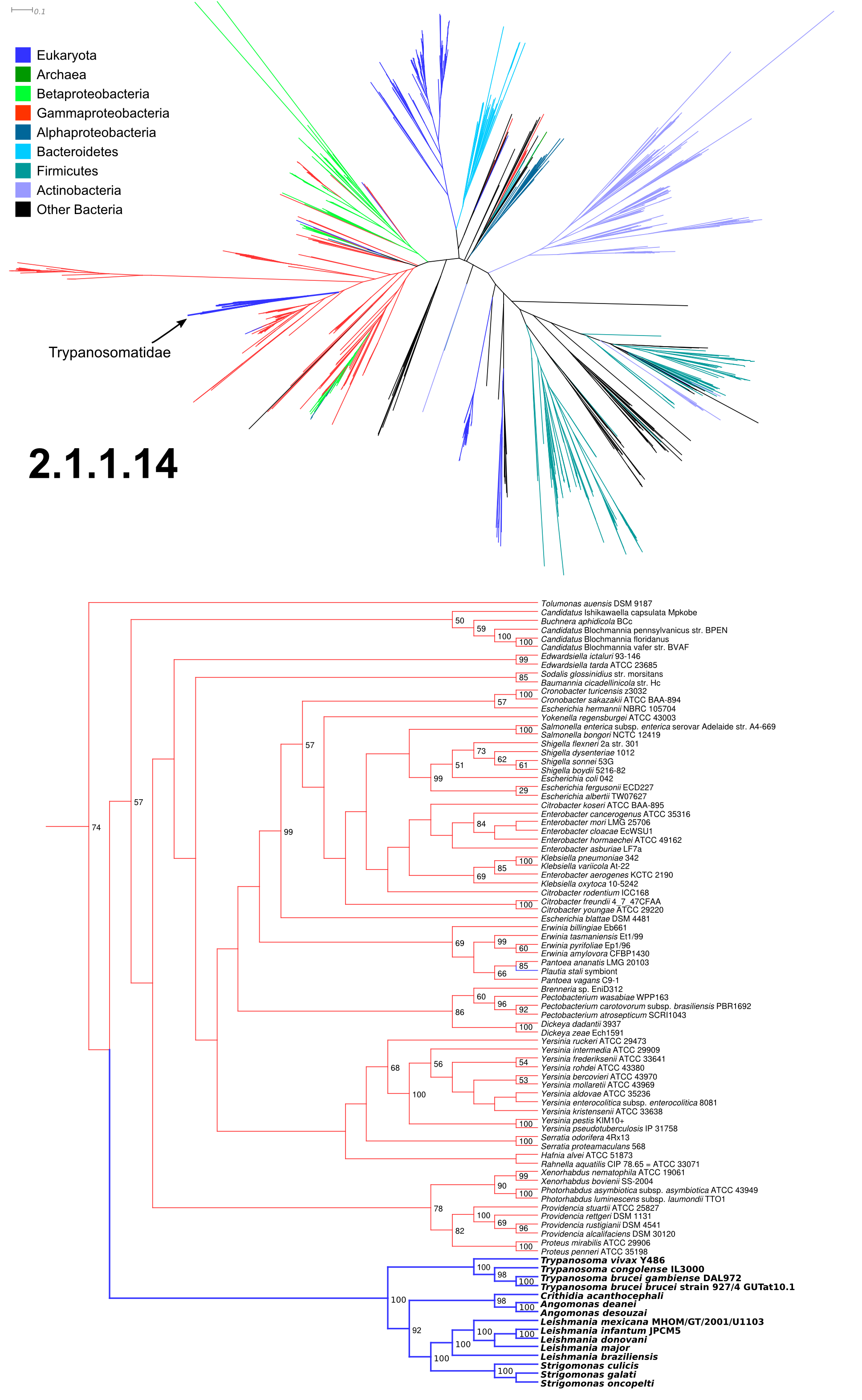

Supplement: Additional file 12 — Maximum likelihood phylogeny of 5-methyltetrahydropteroyltriglutamate-homocysteine S-methyltransferase (EC:2.1.1.14). Overall tree colored according to taxonomic affiliation of sequences. Values on nodes represent bootstrap support (only 50 or greater shown) and distance bar only applies to the overall tree and not to the detailed regions. [file 1471-2148-13-190-S12.png]

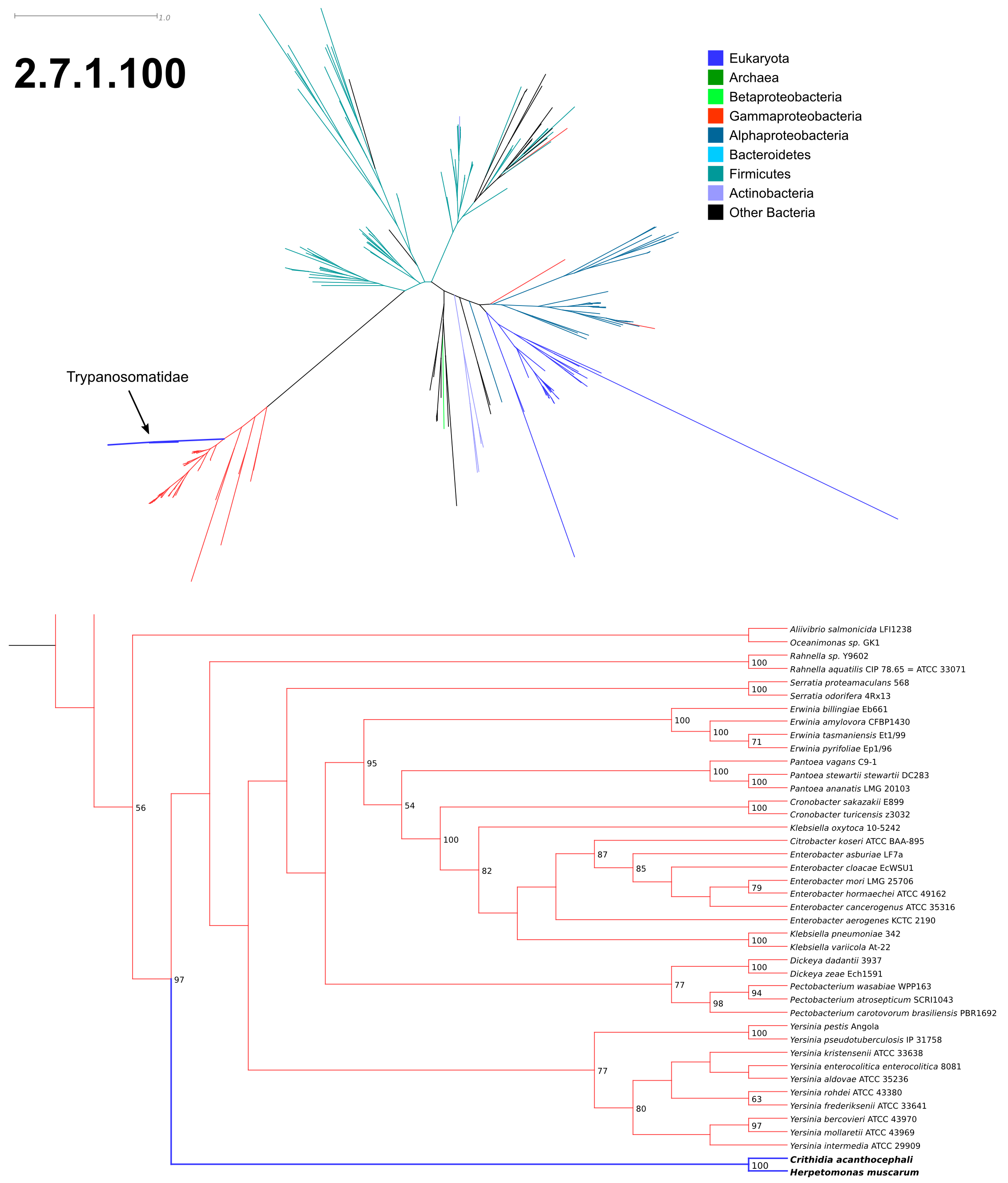

Supplement: Additional file 13 — Maximum likelihood phylogeny of S-methyl-5-thioribose kinase (EC:2.7.1.100). Overall tree colored according to taxonomic affiliation of sequences. Values on nodes represent bootstrap support (only 50 or greater shown) and distance bar only applies to the overall tree and not to the detailed regions. [file 1471-2148-13-190-S13.png]

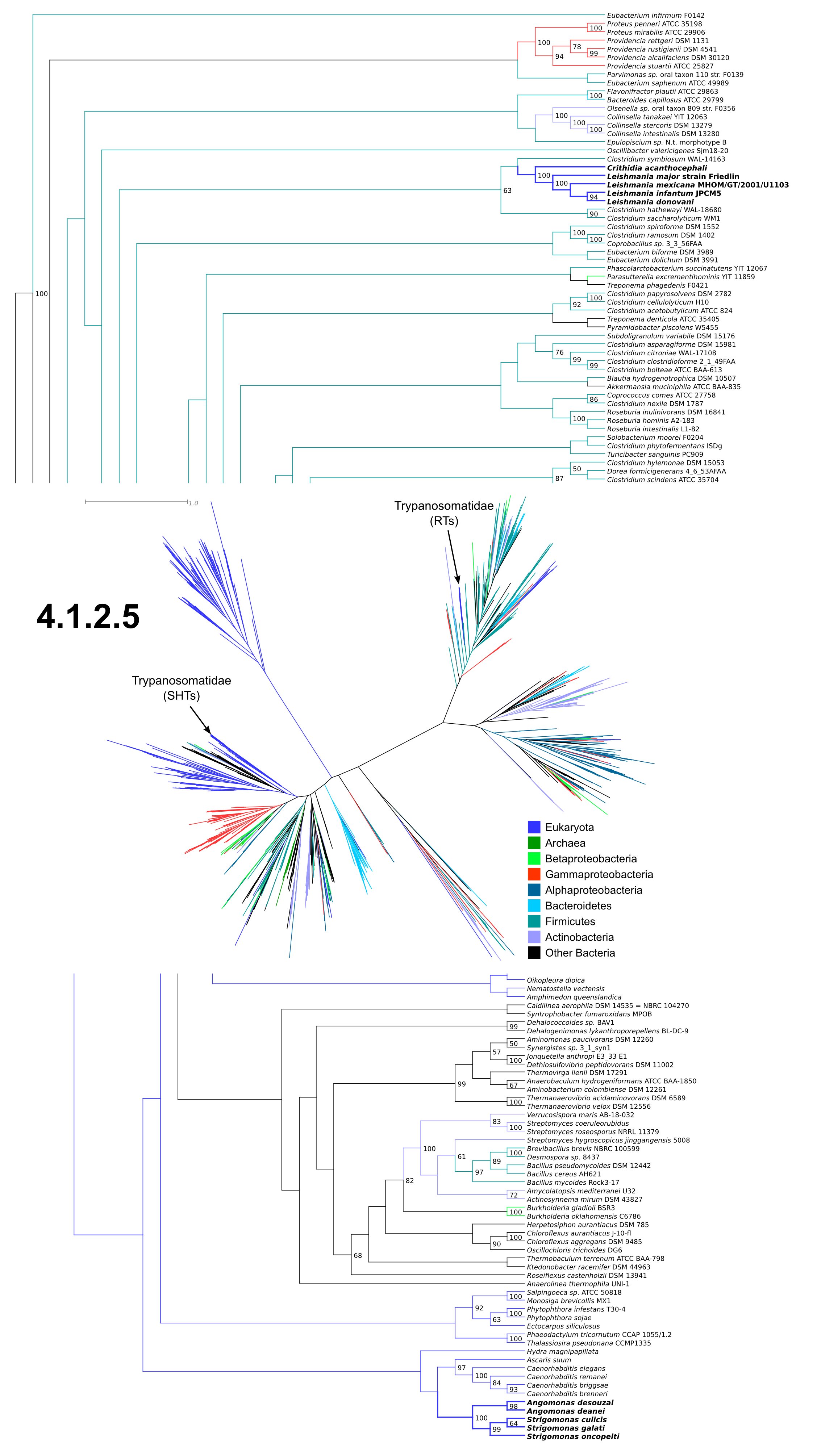

Supplement: Additional file 14 — Maximum likelihood phylogeny of L-threonine aldolase (EC:4.1.2.5). Overall tree colored according to taxonomic affiliation of sequences. Values on nodes represent bootstrap support (only 50 or greater shown) and distance bar only applies to the overall tree and not to the detailed regions. [file 1471-2148-13-190-S14.png]

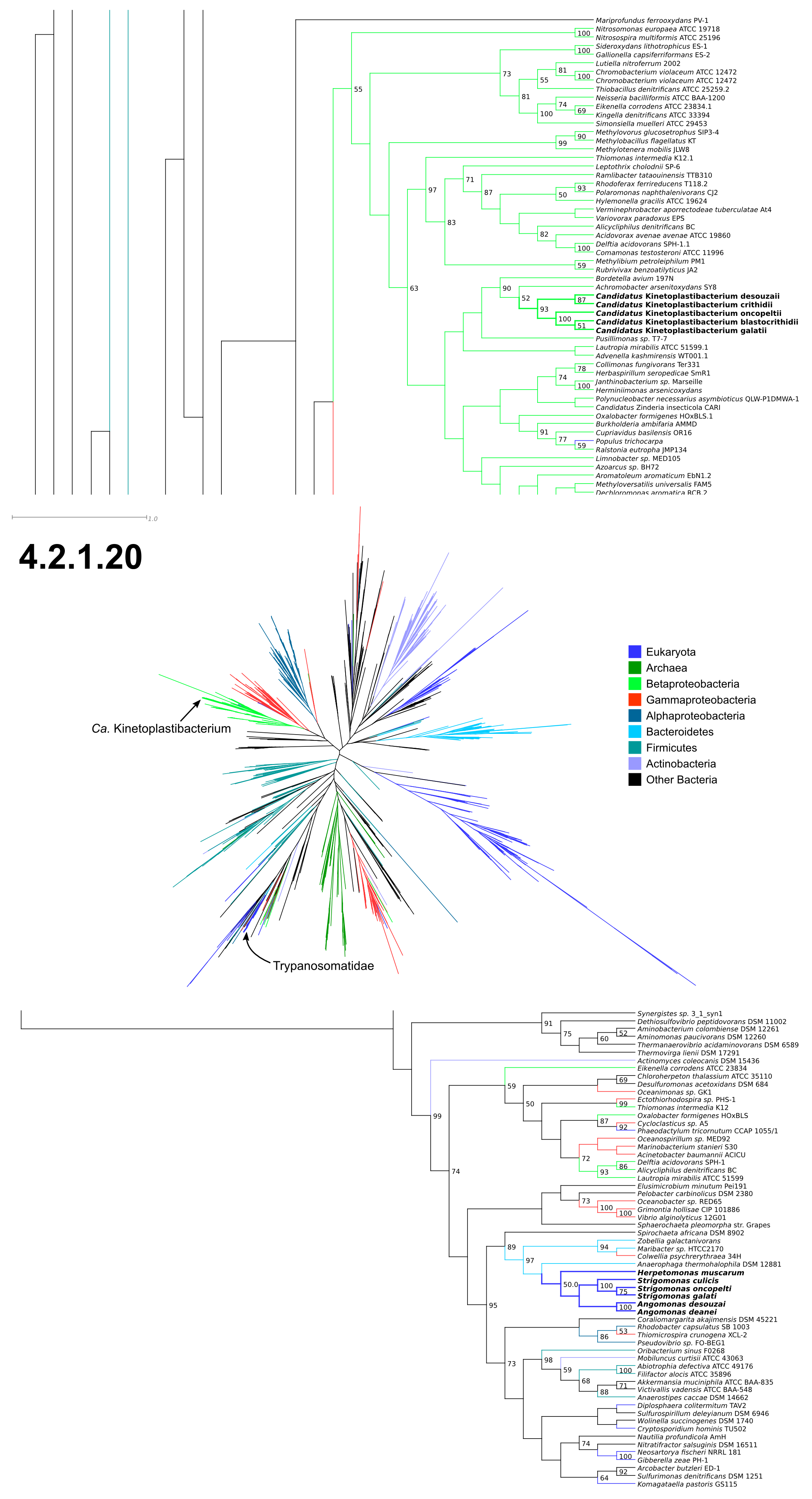

Supplement: Additional file 15 — Maximum likelihood phylogeny of (EC:4.2.1.20). Overall tree colored according to taxonomic affiliation of sequences. Values on nodes represent bootstrap support (only 50 or greater shown) and distance bar only applies to the overall tree and not to the detailed regions. [file 1471-2148-13-190-S15.png]

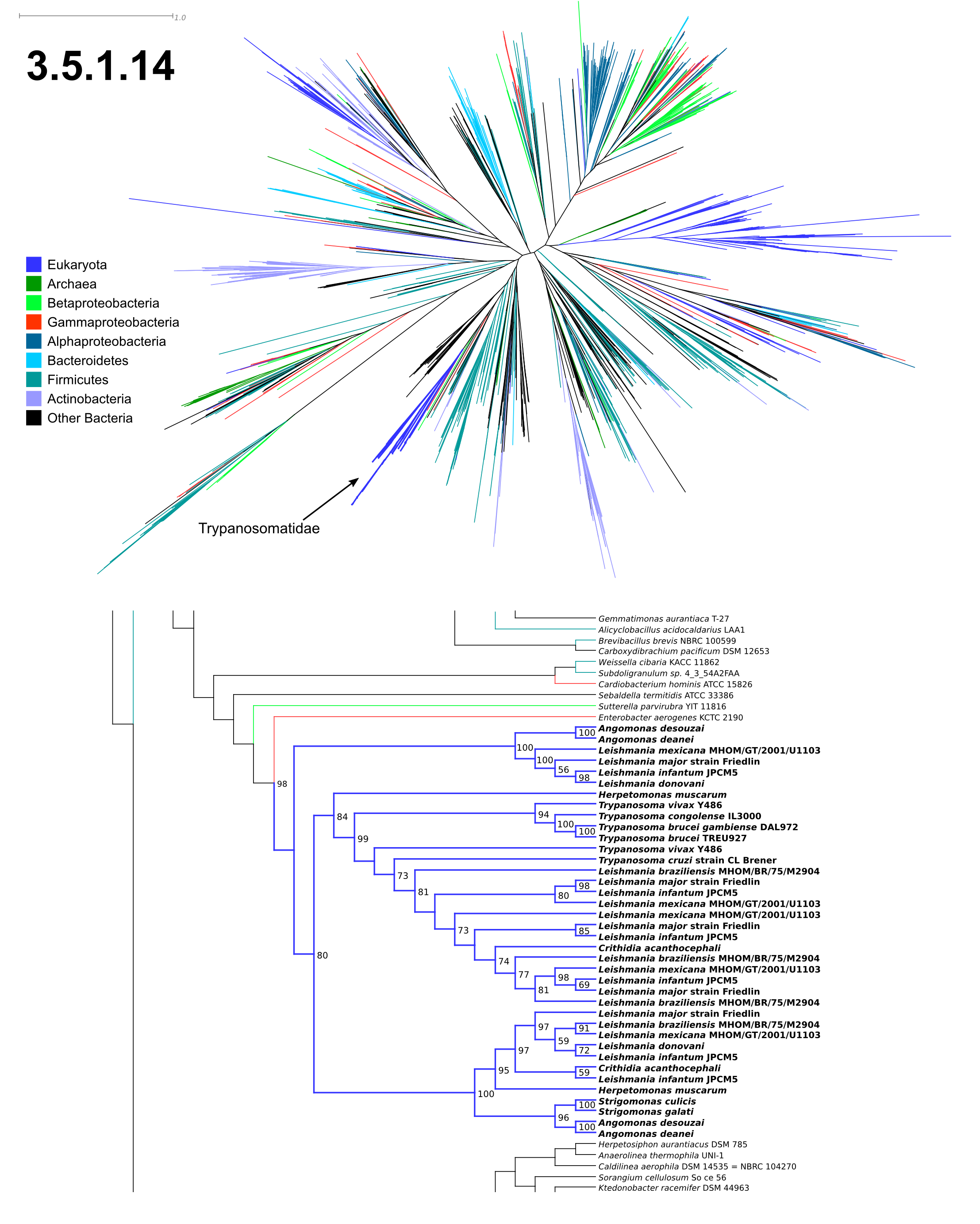

Supplement: Additional file 16 — Maximum likelihood phylogeny of aminoacylase (EC:3.5.1.14). Overall tree colored according to taxonomic affiliation of sequences. Values on nodes represent bootstrap support (only 50 or greater shown) and distance bar only applies to the overall tree and not to the detailed regions. [file 1471-2148-13-190-S16.png]

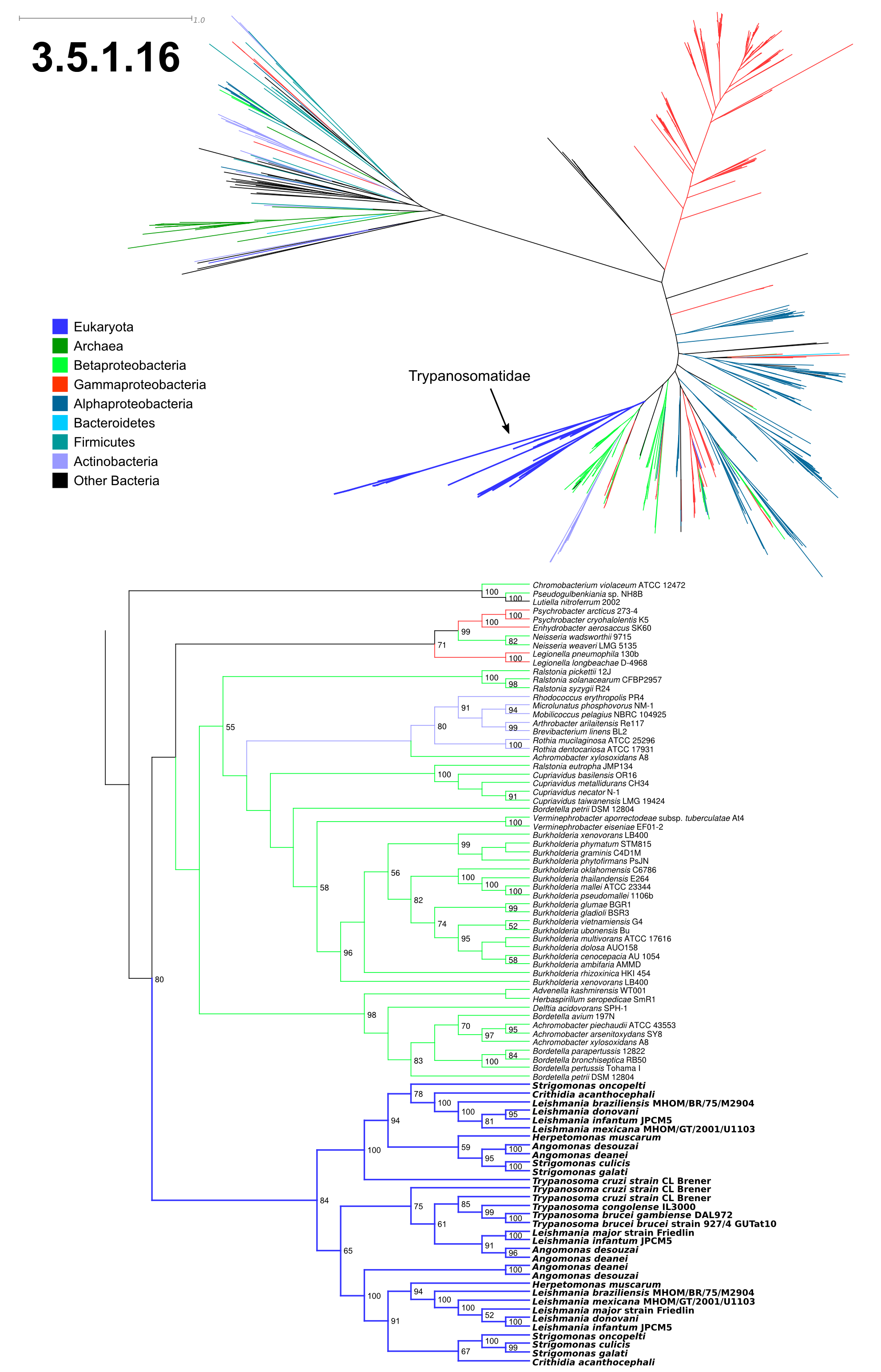

Supplement: Additional file 17 — Maximum likelihood phylogeny of acetylornithine deacetylase (EC:3.5.1.16). Overall tree colored according to taxonomic affiliation of sequences. Values on nodes represent bootstrap support (only 50 or greater shown) and distance bar only applies to the overall tree and not to the detailed regions. [file 1471-2148-13-190-S17.png]

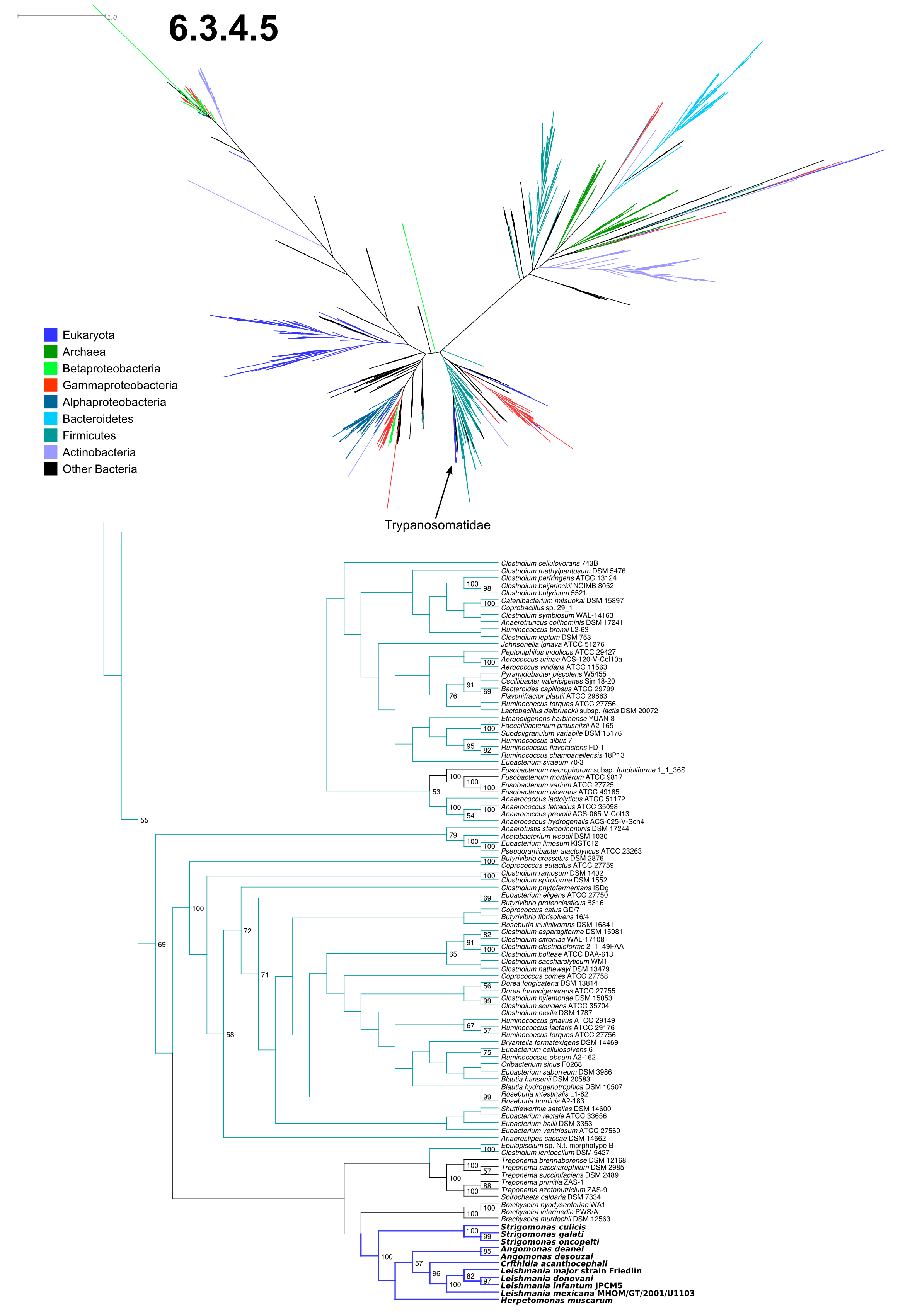

Supplement: Additional file 18 — Maximum likelihood phylogeny of argininosuccinate synthase (EC:6.3.4.5). Overall tree colored according to taxonomic affiliation of sequences. Values on nodes represent bootstrap support (only 50 or greater shown) and distance bar only applies to the overall tree and not to the detailed regions. [file 1471-2148-13-190-S18.png]

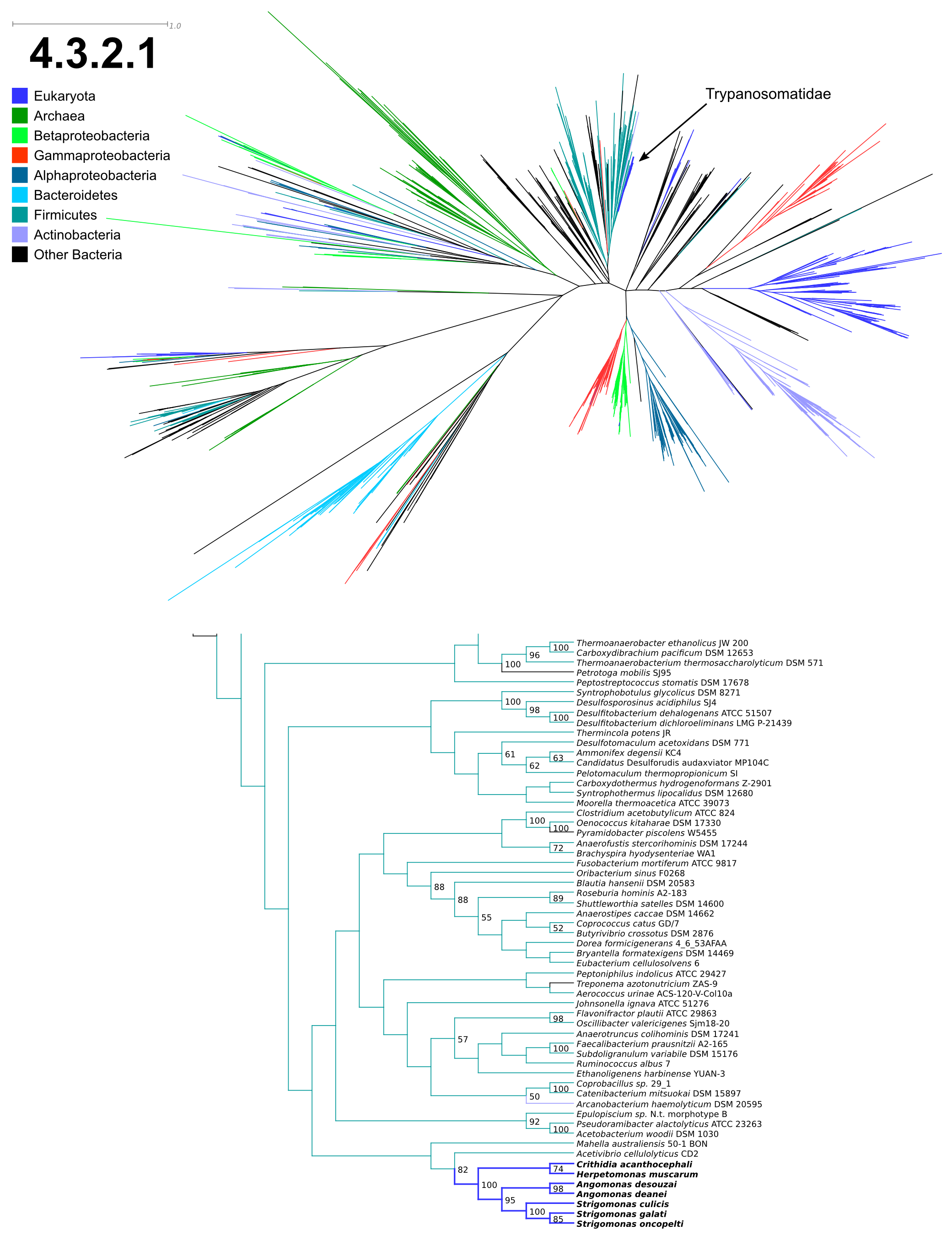

Supplement: Additional file 19 — Maximum likelihood phylogeny of argininosuccinate lyase (EC:4.3.2.1). Overall tree colored according to taxonomic affiliation of sequences. Values on nodes represent bootstrap support (only 50 or greater shown) and distance bar only applies to the overall tree and not to the detailed regions. [file 1471-2148-13-190-S19.png]

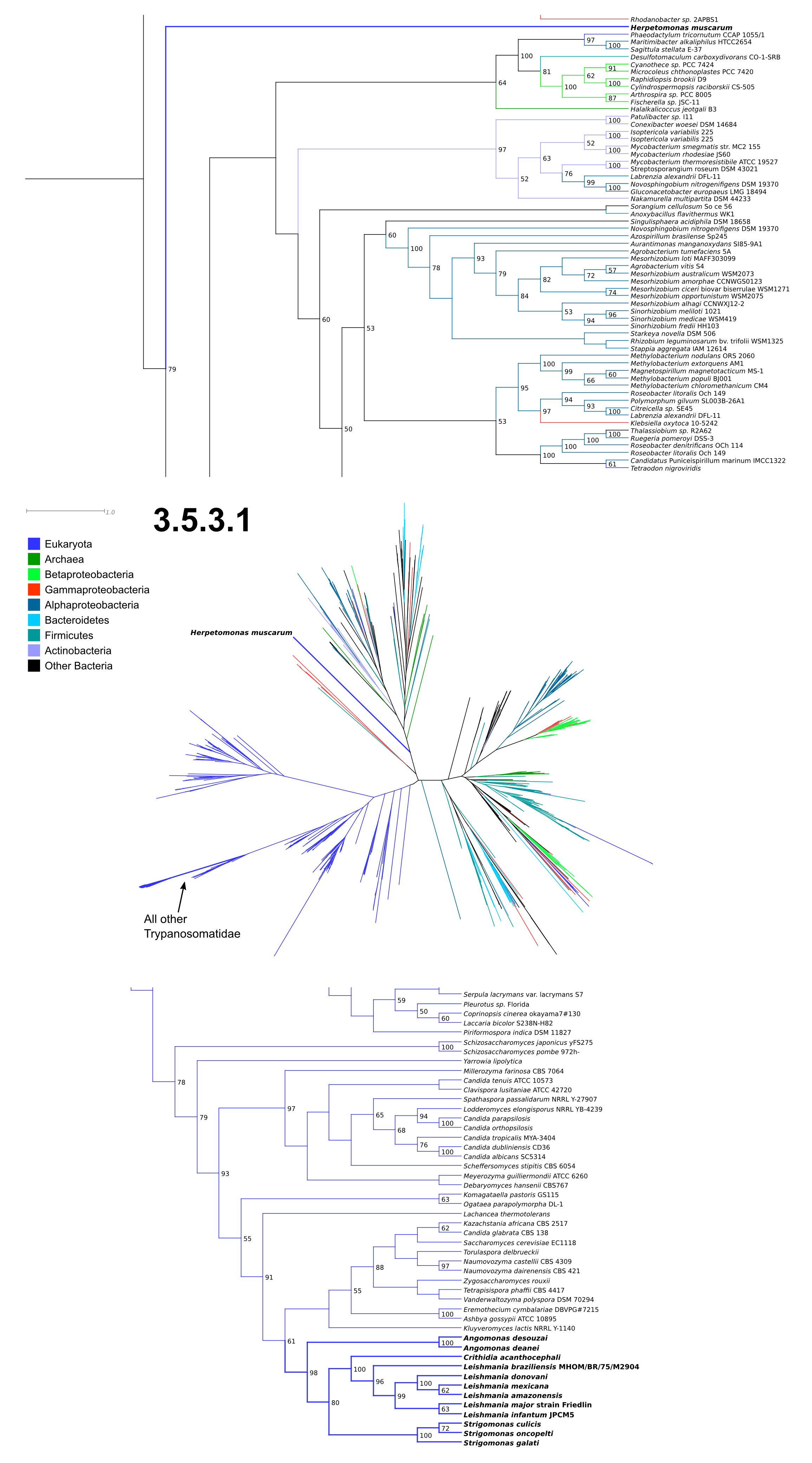

Supplement: Additional file 20 — Maximum likelihood phylogeny of arginase (EC:3.5.3.1). Overall tree colored according to taxonomic affiliation of sequences. Values on nodes represent bootstrap support (only 50 or greater shown) and distance bar only applies to the overall tree and not to the detailed regions. [file 1471-2148-13-190-S20.png]

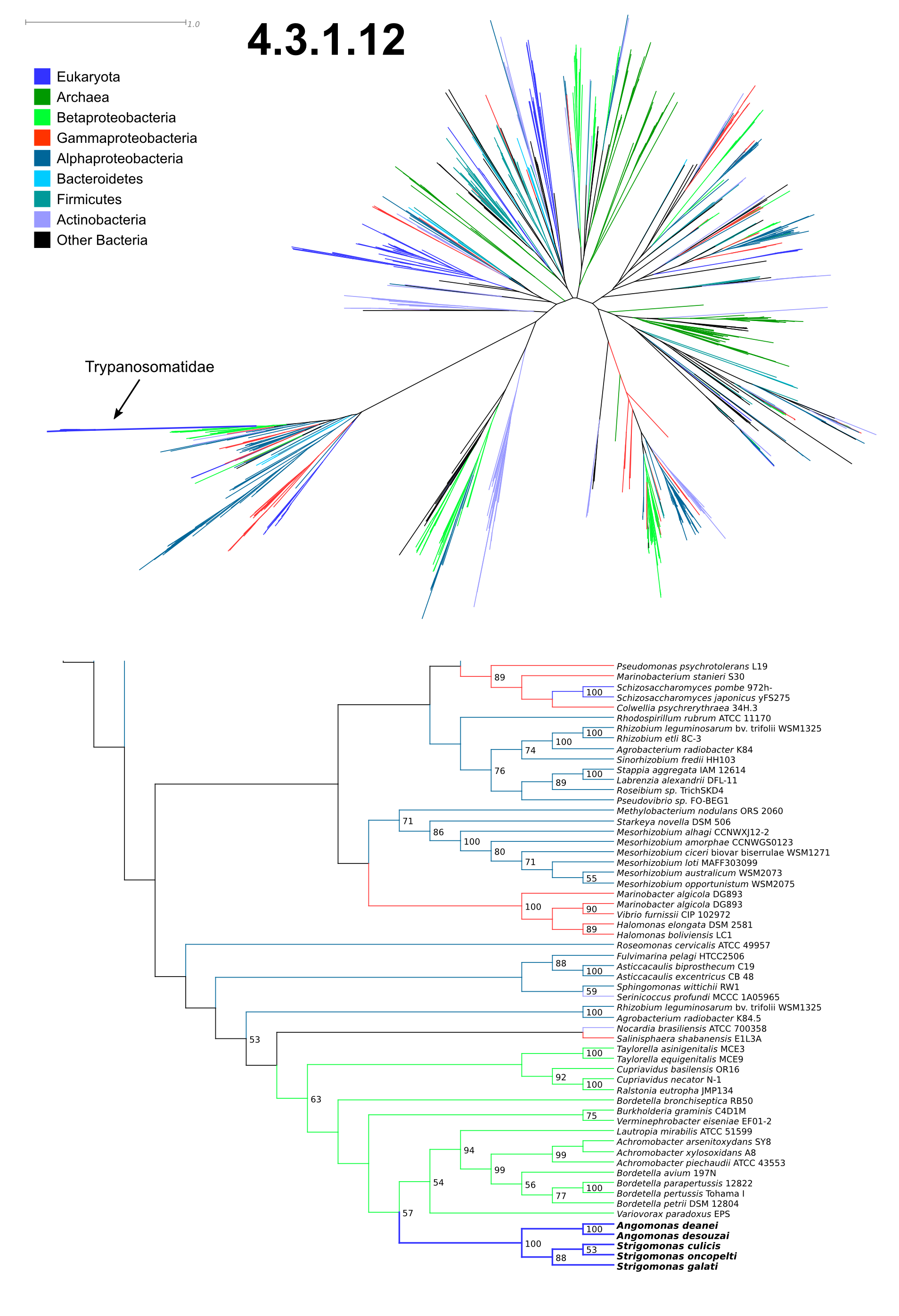

Supplement: Additional file 21 — Maximum likelihood phylogeny of ornithine cyclodeaminase (EC:4.3.1.12). Overall tree colored according to taxonomic affiliation of sequences. Values on nodes represent bootstrap support (only 50 or greater shown) and distance bar only applies to the overall tree and not to the detailed regions. [file 1471-2148-13-190-S21.png]
